# Nanometre to micrometre length-scale techniques for characterising environmentally-assisted cracking: An appraisal

**DOI:** 10.1016/j.heliyon.2020.e03448

**Published:** 2020-03-11

**Authors:** Ronald N. Clark, Robert Burrows, Rajesh Patel, Stacy Moore, Keith R. Hallam, Peter E.J. Flewitt

**Affiliations:** a National Nuclear Laboratory Limited, 102B, Stonehouse Park, Sperry Way, Stonehouse, Gloucestershire, GL10 3UT, United Kingdom; b University of Bristol, Interface Analysis Centre, HH Wills Physics Laboratory, Tyndall Avenue, Bristol, BS8 1TL, United Kingdom; c University of Bristol, School of Physics, HH Wills Physics Laboratory, Tyndall Avenue, Bristol, BS8 1TL, United Kingdom

**Keywords:** Measurement techniques, Environmentally assisted cracking, Steel, Length scale, Nuclear reactor, Materials characterization, Nuclear engineering, Materials science, Materials property, Corrosion, Materials structure

## Abstract

The appraisal is strongly focussed on challenges associated with the nuclear sector, however these are representative of what is generally encountered by a range of engineering applications. Ensuring structural integrity of key nuclear plant components is essential for both safe and economic operation. Structural integrity assessments require knowledge of the mechanical and physical properties of materials, together with an understanding of mechanisms that can limit the overall operating life. With improved mechanistic understanding comes the ability to develop predictive models of the service life of components. Such models often require parameters which can be provided only by characterisation of processes occurring *in situ* over a range of scales, with the sub-micrometre-scale being particularly important, but also challenging. This appraisal reviews the techniques currently available to characterise microstructural features at the nanometre to micrometre length-scale that can be used to elucidate mechanisms that lead to the early stages of environmentally-assisted crack formation and subsequent growth. Following an appraisal of the techniques and their application, there is a short discussion and consideration for future opportunities.

## 1 Introduction

**Figure 1:**
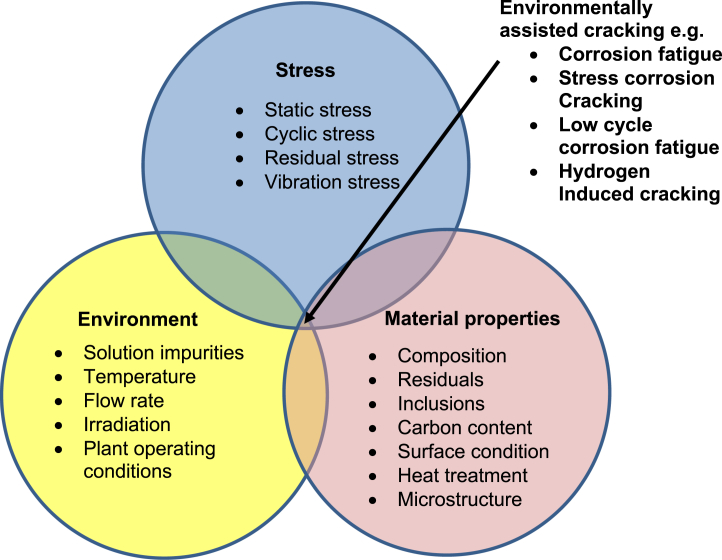
Venn diagram showing the main contributions to EAC^1^.

Environmentally-assisted cracking (EAC) spans several forms, including intergranular attack, stress corrosion cracking (SCC) and corrosion-fatigue [Doig and Flewitt, 1977; Doig and Flewitt, 1983; Speidel, 1980; Tice, 1985; Tice, 2013]. Several major disasters have involved SCC, including the rupture of high-pressure gas transmission pipes, failure of boilers, and destruction of power stations and oil refineries [Cottis, 2000]. It is widely recognised and, indeed, well established that there are three pre-requisites for EAC to occur (Figure 1): (i) a susceptible material; (ii) an appropriate environment; and (iii) a sufficient tensile stress, either applied or residual. In general, SCC occurs under either static or slowly varying loads, whereas corrosion-fatigue is associated with cyclic loads.

**Figure 2:**
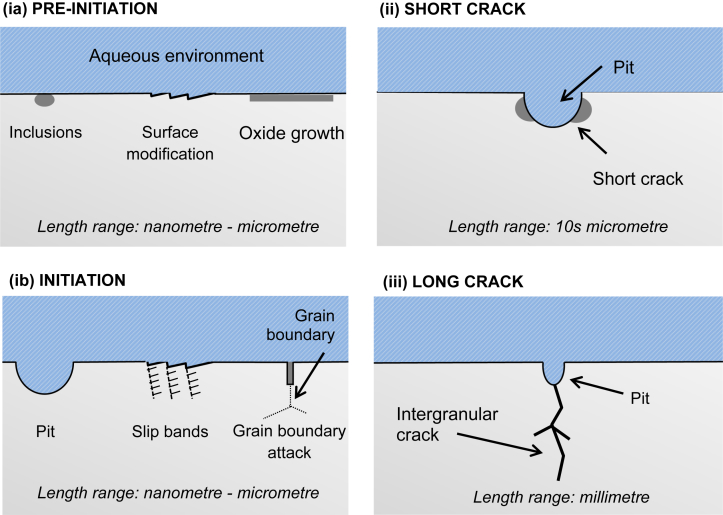
Summary of typical processes at stages associated with the development of cracks in aqueous environments: (ia) crack precursors; (ib) initiation; (ii) short cracks; (iii) long cracks.

In engineering components and structures, the development of cracks in aqueous environments can be characterised into three stages, as illustrated in Figure 2:

(i) crack pre-initiation and initiation
(ii) small cracks
(iii) long cracks

As pointed out by Turnbull [2017], length-scale is an important challenge since there are large data sets related to long cracks, whereas at the shorter length-scales both qualitative and quantitative characterisation of damage and crack growth need to be resolved. Certainly, in the first stage there is degradation of a smooth surface as a precursor to cracking. As shown by Doig and Flewitt [1977], this stage can involve localised corrosion at preferential sites, leading to the development of occluded cells that fill with corrosion products. As a consequence, local corrosion pits are frequently formed. The next stage combines the local stresses and strains associated with these pits to initiate SCC. Turnbull [2017], using X-ray computed tomography (XCT), showed that the transition from a pit to a crack does not necessarily occur at the base of such a pit. Rather, it can occur at the side of a pit, where finite element (FE) analysis revealed localised dynamic strain is sufficient to initiate a stress corrosion crack. The final stage is crack propagation, where the growth rate is controlled by the material microstructure and composition along the crack path, the applied stress intensity and the solution environment. At that stage, the crack can follow either a transgranular or intergranular path. However, as shown by the Venn diagram in Figure 1, the complexity of these many contributions makes it difficult to ensure SCC will not be encountered in plant components subject to long-term operation, because of the challenges of potentially changing material properties, environment and service load.

**Figure 3:**
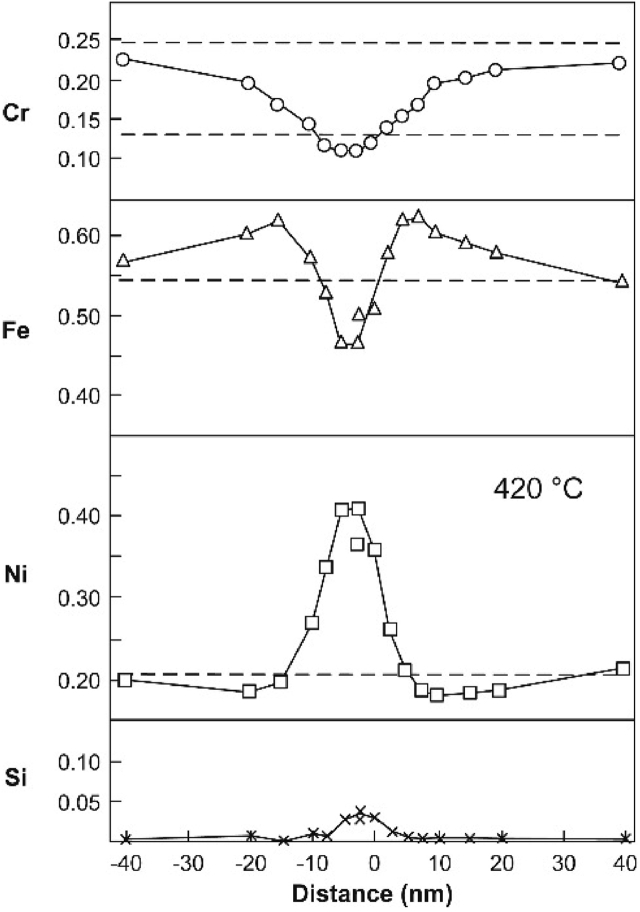
Line profiles across a grain boundary of 20Cr–25Ni–Nb AGR reactor fuel cladding from an RIS-affected lower fuel element (stringer B4286). Zero is the position of the grain boundary and the irradiation temperature is given in the inset. From Whillock et al. [2018].^2^

Thermal degradation of stainless steels over extended periods of operation can lead to precipitation of chromium carbides at grain boundaries. The formation of these precipitates causes an adjacent region at the grain boundary precipitates to be locally depleted of chromium [Flewitt and Wild, 2001]. As chromium is the primary passivating element in stainless steels, once the boundary regions are sufficently below a chromium threshold (commonly ~12% for a stainless steel [Jain et al., 2010]) depassivation occurs, and these sites are susceptible to enhanced anodic dissolution. (For 20Cr–25Ni–Nb stainless steel advanced gas-cooled reactor (AGR) fuel cladding such regions have typical widths of 5–15 nm produced through the radiation-induced segregation (RIS) mechanism; typical depleted zone widths from thermal sensitisation mechanism are wider, >30 nm [Norris et al., 1990]). This process is known as thermal sensitisation and, in the presence of a corrosive environment, leads to intergranular corrosion (IGC). Typically, sensitisation occurs at heat-affected zones of welds in high-carbon-content stainless steels, those not stabilised by niobium or titanium, or following poor post-weld heat treatment. In the presence of stresses, SCC can propagate along sensitised grain boundaries, *i.e.* intergranular stress corrosion cracking (IGSCC). Similarly, extended exposure to a combination of neutron irradiation and high temperature which, through a point defect diffusion mechanism (RIS) [Bruemmer et al., 1999; Norris et al., 1990; Whillock et al., 2018, Clark et al., 2020]), can lead to local chromium depletion at grain boundaries. Figure 3 shows line composition profiles taken across a sensitised grain boundary of an ex-service 20Cr–25Ni–Nb AGR fuel cladding specimen, highlighting the extent of chromium and iron depletion and enrichment of nickel and silicon through the RIS process. The locally-depleted chromium at the grain boundary again provides a susceptible material to IGC and, in the presence of stresses, can lead to irradiation-assisted stress corrosion cracking (IASCC).

It is recognised that, in service, EAC initiates from several surface features, including pits, de-alloyed layers, inclusions and precipitates, physical defects, such as laps, and slip bands. These control early stages of formation but, in addition, the rates of growth of subsequent cracks are complex, so it is important to understand the mechanisms that control at the nanometre-to-micrometre length-scale. In this context, the factors of local chemistry and electrochemistry, near-surface microstructure, local mechanical properties and both short- and long-range residual stresses are important. Moreover, as pointed out by Turnbull [2012], crack size effects are very significant in low-conductivity solutions because of the differences in potential drop associated with these small or short cracks compared with long macro-cracks. Indeed, this is a key feature of the work of Turnbull and Ferriss [1986].

For civil nuclear electrical power generation in the UK, the structural integrity of key components has been recognised to be an essential consideration for both safety reasons and economic operation. In addition to extending the operating life of the fleet of AGRs to about forty years, both new-build plant and designs for future, fourth generation (Gen IV) fission reactors will be required to operate for periods of sixty years or more. Existing materials and methodologies face challenges to achieve this ambition. An essential input for demonstrating the necessary assurance is structural integrity assessment [Flewitt, 1995]. SCC and corrosion-fatigue, in both BWR and PWR plants has resulted in coolant leakage rather than catastrophic failure [Tice, 2013]. In many cases, in-service inspection has identified such cracking, and examples are shown in Table 1 [Flewitt, 2016].

**Table 1:** Examples of typical materials and associated components identified to be subjected to EAC [Flewitt 2016].

Material	Alloy	Application
Ferritic steels	Low-alloy steels, such as A533B and A508-III	Main pressure vessels
	Carbon steels	PWR steam generator tubesheets, piping in some BWRs and various other piping applications, and high-strength quench and tempered steels used for bolting applications
Stainless steels	Austenitic (300 series)	Use as AGR fuel cladding material, widely used in both BWRs and PWRs for piping, pump and valve bodies, and a variety of other applications
	Martensitic stainless steels	Employed where higher strengths are required, such as in valve stems and fastener applications
	Precipitation-hardened alloys, such as A-286 and 17-4PH	High-strength applications
Nickel base alloys (NBA)	The weld metal alloys 182, 82 and, in Japan, 132, Alloy 690 and weld metal alloys 52, 152 and variants are being widely used for replacement and new-build due to the relatively poor service experience of Alloy 600	Steam generator tubing, control rod drive penetrations, bottom head penetrations, steam divider plates
	Alloy 690	Used as replacement for alloy 600 and in new build
	Alloy 52, 82, 132 (Japan), 152, 182	NBA weld metals
	Precipitation-hardened alloys such as X-750 and 718	High strength requirements, such as internal bolting, fasteners and springs

There is clearly a requirement to develop mechanistically-based models to predict the service lives of components with improved confidence. To achieve the required periods of operation such models invoke parameters which can be provided only by characterisation of processes occurring *in situ* over a range of length-scales, with the sub-micrometre-scale being particularly important, but also challenging [Scully, 2015]. This appraisal seeks to review the techniques currently available at the nanometre-to-micrometre length-scale and that can be assembled into appropriate toolboxes to facilitate establishing mechanisms leading to the early stages of EAC formation and subsequent growth. In Section 2, we summarise the local mechanisms that have been invoked to describe crack extension. In Section 3, we consider the various experimental methods available to undertake key measurements at the nano-to micro-scale to enable understanding of the controlling mechanisms. These are further explored in Section 4 and considered in the context of opportunities for application or development.

## 2 Local mechanisms

The local mechanisms described within this section are a result of the interaction of corrosion, material properties and stress that produce failure by EAC. There are three main parameters that influence the susceptibility of a system to EAC, or specifically, SCC (Figure 1). However, within each of these are a large range of variables that make the interactions complex and, indeed, prediction of susceptibility difficult. Examples of the range of potential variables are summarised in Table 2. It is important to address these contributions if a complete understanding of the underlying mechanisms that control the initiation and growth of cracks in aqueous environments is to be established. This is clearly a complex issue, but essential as an input to establishing simplified, predictive models that are required to provide high confidence in evaluating the service life of nuclear plant components.

**Table 2:** Parameters known to influence SCC, categorised into three main areas.

Environment *It should be noted that the crack tip environment can differ significantly from the bulk solution*	Temperature
	Pressure
	Solute species
	Solute concentration and activity
	pH
	Electrode potential
	Solution viscosity
	Stirring or mixing
	Radiation
Stress	Primary loading (applied stress)
	Secondary loading (residual stress)
	Stress intensity factor
	Mode of loading at the crack tip (*e.g.* tension or torsion)
	Plane stress or plane strain conditions
	Length, width, aspect ratio of the crack
	Crack opening and crack tip closure
Microstructure properties	Mechanical properties (yield stress, ultimate tensile strength, fracture toughness)
	Second phases present in the matrix and at the grain boundaries
	Composition of phases
	Grain size
	Surface finish
	Irradiation effects (e.g. grain boundary segregation, radiation swelling, radiation hardening, creep relaxation)

An additional EAC process, IASCC, is of relevance to reactor core component materials during reactor operation, which is a demanding environment, as materials are subject to not only high temperatures and pressures, but also to extreme radiation fields [Was and Andresen, 2012; Bruemmer et al., 1999]. The general processes are the same as with SCC, although the effect of irradiation can cause a change to the microstructure, which must be taken into consideration, as this can assist the SCC process. As mentioned prior, irradiation can lead to segregation along grain boundaries (RIS), but can also lead to radiation hardening, helium/ hydrogen embrittlement, swelling, and creep relaxation [Was and Andresen, 2012]. This radiation damage has a significant effect on both the material microchemistry and microstructure. The environment itself is also affected by radiation (radiolysis of water), which causes the formation of molecular and ionic products of water, including the formation of free radicals, which can react with stable species. Such reactions occur within the order of micro-seconds; with the most stable molecular products being O_2_, H_2_, and H_2_O_2_. It is the presence of oxidising species such as O_2_ and H_2_O_2_ (which serve to raise the electrode potential), combined with the radiation-induced changes to the microstructure that can significantly enhance SCC. The IASCC phenomenon has been observed in both boiling water reactors (BWR) and pressurised water reactors (PWR), and indeed all types of water-cooled reactors. Formation of oxidising species (which can be formed through radiolysis of water) can be effectively supressed by an H_2_ overpressure in the coolant water (>500 ppb [H_2_]) which leads to a reducing environment [Was and Andresen, 2012]. Due to the nature of BWRs, whereby the coolant partitions from the liquid to the gaseous phase, BWRs cannot achieve the same additions of H_2_ in-core compared to PWRs, therefore the effects of radiolysis are greater relevance to BWRs [Scott, 1994]. More detailed information on the IASCC mechanism can be obtained in [Andresen and Was, 2019; Zinkle and Was, 2013; Was and Andresen, 2012; Was and Bruemmer, 1994; Scott, 1994].

There are numerous local mechanisms that have been developed over the years to describe SCC, and the main ones are considered within this document. These are: hydrogen embrittlement; adsorption-induced cleavage; surface mobility; film rupture; film-induced cleavage; and localised surface plasticity. Associated with these mechanisms is the formation of a crack, or a multiple-crack system, which can be categorised into pre-initiation, initiation and crack growth stages. Pre-initiation arises from, in general, local time-dependant modificaitons to the surface condition, such as oxide film formation, initiation includes pitting and intergranular attack, and growth includes both short cracks and the transition to long cracks, as shown in Figure 2(iii). It is important that any mechanism explains crack propagation rate, fractographic evidence, crack path and the means of formation or initiation of cracks. The mechanisms usually assume that interatomic bonds at the crack tip break by either chemical solvation and dissolution or mechanical fracture (ductile or brittle). Mechanical fracture includes processes that are assumed to be either stimulated or induced by one of the following interactions between the material and the environment: adsorption of environmental species; surface reactions; reactions in the metal ahead of the crack tip; or formation of surface films. All of the proposed mechanical fracture mechanisms contain one or more of these processes as an essential step in the SCC process. Specific mechanisms differ in the processes assumed to be responsible for crack propagation and the way that environmental reactions combine to result in fracture [Jones, 1992].

As discussed by Lynch [2011a], at the atomic level, there are four underlying mechanisms for SCC crack growth:

• Dissolution of atoms at the crack tip into solution
• Shear forces acting upon atoms at crack tips
• Decohesion of atoms at crack tips (tensile separation of atoms)
• Diffusion of atoms on the surface of crack tips to behind the crack tip

**Figure 4:**
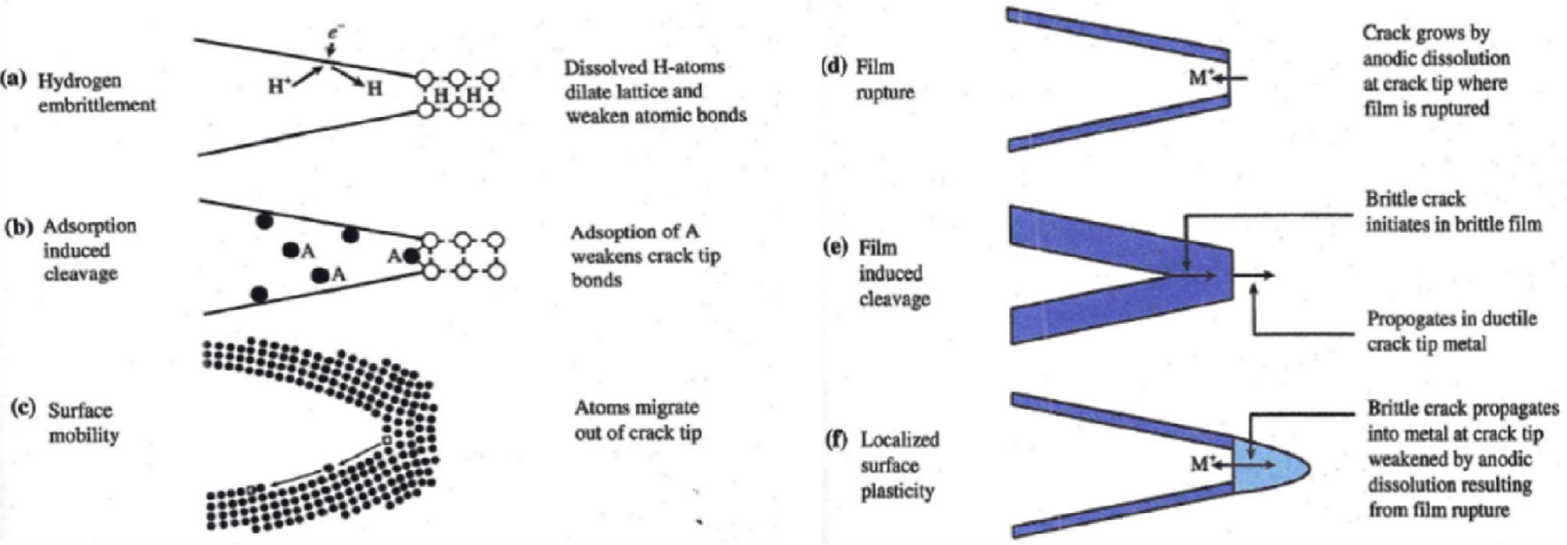
Schematic of crack tip processes that may occur during environmentally-assisted crack propagation Adapted from Was [2007].^3^

Whilst Figure 2 identifies the typical stages of SCC, Figure 4 illustrates the processes which occur at the crack tip. These mechanisms are summarised in Figure 4 and described below.

Figure 4(a) shows the hydrogen embrittlement mechanism. Hydrogen dissolves in all metals to a moderate extent as the hydrogen atom is very small (~106 pm) and fits in the interstitial positions in the atomic lattice of crystals. Moreover, since it is a small atom and is readily absorbed it can diffuse much more rapidly than larger atoms [Kim et al., 2014; Kiuchi and McLellan, 1983]. Hydrogen tends to be attracted to regions of high-triaxial tensile stress, where the metal structure is dilated, and, hence, is attracted to regions ahead of cracks or notches that are under stress [Raub and Bullough, 1985]. The dissolved hydrogen leads to embrittlement of the metal, and cracking may be promoted by either intergranular or transgranular processes. Crack growth rates are relatively rapid, up to 1 mm s^−1^ in the most extreme cases. The main parameters to consider for hydrogen embrittlement are related to material properties in terms of the size and relative position of secondary phase precipitates, grain geometry and grain boundaries. The availability of hydrogen at the crack tip is a fundamental parameter for this mechanism, and may be affected by material pre-treatment (hydrogen charging or discharging), local chemistry and electrochemistry [Burns et al., 2016]. As with most mechanisms, it is necessary to measure mechanical properties at the nano-scale, for example by evaluating K_I_ and K_scc_ values.

The adsorption-induced cleavage model (Figure 4(b)) is based on the concept that adsorption of environmental species lowers the interatomic bond strength and stress required to propagate a cleavage crack [Lynch 1988, 2009; 2011a]. This model can satisfactorily explain the susceptibility of some alloys for cleavage crack propagation when the alloying elements are bonded to certain ions. One of the important factors in support of this mechanism is the existence of critical electrode potentials below which SCC does not occur. This model of cracking underlines the relationship between the value for the potential and the capacity of adsorption of the aggressive ion. This approach provides an explanation for the prevention of SCC by cathodic protection.

The surface-mobility mechanism (Figure 4(c)) for SCC assumes that stress concentration at the tip of a crack generates a very localised vacancy-deficient region. The capture of vacancies by the tip of the crack leads to propagation. The systems studied so far indicate that surface contaminants act by increasing surface mobility and by supplying the vacancies required by the SCC process [Galvele, 1996]. The postulates for this mechanism are: (i) the environment affects the metal by changing surface self-diffusivity; (ii) the temperature at which SCC takes place is lower than 0.5 T_m_; (iii) only elastic stresses are relevant in the SCC process; and (iv) SCC occurs by capture of vacancies by the tip of the crack. If the temperature is known, crack growth rates can be predicted. Very slow crack propagation rates, *e.g.* 1 x 10^−12^ m s^−1^, or even lower, are relevant to the nuclear industry, which typically has long project time-scales, such as in steam generators, pressure vessels and waste repositories. Galvele [1996] states that these particularly-low crack growth rates were very difficult to measure with the techniques available at the time that the model was proposed, some twenty years ago.

The film rupture model (Figure 4(d)) considers that crack growth occurs by extremely-localised anodic dissolution. The sides of the crack are protected by a film, usually an oxide, which is fractured as a result of plastic strain in the metal at the crack tip [Andresen and Ford, 1988]. Crack growth develops by the cyclic process of film rupture, dissolution and repair. In general, there is good correlation between the average oxidation current density on a straining surface and the crack propagation rate for a number of systems. It has been argued by Sieradzki and Newman [1987] that pre-existing active path mechanisms cannot work unless corrosion is directional, *e.g.* along a grain boundary [Lozano-Perez, 2002]. Hence, within this mechanism cracking is intergranular. Other parameters, such as visualisation of surface topography, pitting, surface dissolution and formation of a passive film, can be measured directly, for example by electrochemical scanning tunnelling microscopy (EC-STM) [Seyeux et al., 2008]. Also, an indication of galvanic activity and corrosion can be obtained by measuring local gradients in potential and chemical current flow using, for example, the direct scanning vibrating electrode technique (SVET) [Clark et al., 2020; Laferrere et al., 2017; Williams and McMurray, 2006].

The film-induced cleavage model (Figure 4(e)) gives an alternative mechanistic understanding when pre-existing active paths are apparently non-existent or inoperative [Lozano-Perez, 2002]. If a normally-ductile material is coated with a brittle film, then a crack initiated in that film can propagate in the ductile material for a small distance (~1 μm) before being arrested by ductile blunting. If the brittle film has been formed by a corrosion process then it can reform on the blunted crack tip and the process can be repeated. This film-induced cleavage process would normally be expected to give transgranular fracture [Cottis, 2000]. The active path along which the crack propagates is cyclically-generated as alternately disruptive strain and film build-up, or the propagation is related to the slip characteristics of the underlying metal. This strain-generated active path mechanism would be expected to result in transgranular stress corrosion cracking (TGSCC). Support to these theories is given by observations that the crack propagation rates for many systems are in direct proportion to the experimentally-determined dissolution rates under the mechanical and chemical conditions expected at the crack tip. This assumes that the potential at the tip of the tight crack is the same as at the specimen surface [Lozano-Perez, 2002]. The important parameters to be measured for this mechanism are: (i) surface topography; (ii) pitting; (iii) surface dissolution; and (iv) formation of passive films.

The localised surface plasticity mechanism (Figure 4(f)) proposes that SCC results from the effect of the local microstructure ahead of the crack tip. This mechanism assumes that galvanic corrosion occurs between active sites and surrounding passive surfaces. Repassivation of the active sites is prevented by the presence of a weakened surface passive film at these locations, possibly due to the local electrochemical conditions remaining below the critical passivation potential. The locally-high anodic current densities arising from the galvanic couple cause the formation of a defect structure, which becomes a rupture site. The softened structure of the rupture sites plastically deforms in the microscopic volume ahead of the crack, and this is enhanced by the presence of surrounding passive material with higher strength. A weakened and constrained crack tip volume creates a triaxial stress state that results in the propagation of brittle cracks [Popov, 2015].

There are various reviews which address the underlying mechanisms of EAC in greater detail, such as those by Lynch [2011a, 2011b]. For further information relating to nickel base alloys specifically, the reader is directed to the recent monograph by Scott [2019].

Certainly, there is a need to develop a toolbox of measurement techniques across the nanometre-to-micrometre length-scale, to allow the appropriate environment, electrochemical, material, microstructural and stress measurements to be made that would develop understanding of the mechanisms that lead to initiation and growth of environmentally-assisted cracks in austenitic and ferritic steels, and nickel-based alloy components. In Section 3, we explore the considerable progress that has been made in advanced characterisation techniques that could enable the required parameters to be quantitatively measured.

## 3 Nano-to micro-scale techniques and applications

### 3.1 Introduction

As described in Section 2, the formation of macroscopic, environmentally-assisted growing cracks can be divided into several stages, with each requiring measurement of key parameters of the type summarised in Figure 3 and Table 3. There have been significant advances in techniques that have the potential to make such measurements of environment, stress and microstructure across the atomic-to-millimetre length-scale (*i.e.* pre-initiation to long crack scale). These are now considered and, for this, we divide the techniques into direct and indirect. We define direct as those techniques where all three parameters act conjointly, and progress of the failure mechanism can be monitored continuously. In comparison, the indirect methods provide a measure of contributions from individual parameters and intermittent evaluation by removal from the test environment. An overview of all techniques discussed within this document is given in Table 3 (direct/indirect; parameter measured; scale resolution; constraints). This table can be used to understand how various techniques may be used to elucidate understanding of EAC crack processes across the length-scales (nm; μm; mm).

**Table 3:** Summary of parameters measured, and indicative length- and time-scales, for each group of techniques.^4^

Technique	Abreviation	Parameter measured	Direct?	Scale dimension	Data acquisition time resolution	Constraints
***Surface***						

Optical microscopy		Imaging, displacement, event timing and location	Y	10^−6^ m *in situ*	10^−3^ s	
Scanning and transmission electron microscopy	SEM, TEM	Imaging, displacement, chemical distribution, texture	N	10^−9^ m (SEM) 10^−11^ m (TEM)	10^2^–10^4^ s	High vacuum (HV), <10^−4^ Pa (SEM) Ultra high vacuum (UHV), 10^−7^ - 10^−9^ Pa
Environmental scanning and transmission electron microscopy	ESEM, ETEM	Imaging, chemical distribution, texture, residual stress	Y	10^−9^ m	10^2^–10^4^ s	Resolution reduced by environmental conditions; water opaque to electrons.
Focused ion beam sectioning/ imaging	FIB	Imaging, texture	N	10^−9^ m	10^2^–10^4^ s	HV, <10^−4^ Pa
Scanning probe microscopy/ atomic force microscopy	SPM, AFM	Topography	N	10^−9^ m	10^3^–10^4^ s	Surface preparation
High speed atomic force microscopy	HS-AFM	Topography, micro-stiffness, electrical/thermal conductivity	Y	10^−9^ nm (XY resolution) ±15 x10^−12^ m (Z resolution)	10^−3^ – 10^−1^ s	Surface preparation
Electrochemical scanning tunnelling microscopy	ESTM	Topography, local electrochemistry	Y	10^−10^ m	10^3^–10^4^ s	
Optical spectroscopy		Elemental and chemical structure	Y (some)	10^−9^ m	10^2^–10^4^ s	Some techniques are HV; some require idealised surfaces
Secondary ion mass spectrometry (including NanoSIMS)	SIMS, NanoSIMS	Elemental mapping (including isotopic)	N	10^−6^ m (SIMS) 10^−8^ m (NanoSIMS)	10^5^ s	HV, 10^−4^ Pa (SIMS) UHV, <10^−5^ Pa (NanoSIMS)
***Volume***						

X-ray tomography	XCT	Internal structure, development, density	Y	10^−7^ m	10^2^–10^4^ s	
High-resolution, small-scale focused ion beam tomography		Internal structure, texture, residual stress, chemical mapping	N	10^−9^ m	10^4^–10^5^ s	
Electron tomography		Structure, texture, residual stress, chemical mapping	N	10^−10^ m	10^4^–10^5^ s	
Atom probe tomography	APT	Elemental distribution	N	10^−11^ m	10^5^–10^6^ s	Very small sub-sampling, UHV, ~10^−8^ Pa
***Reaction sensing***						

Macro-scale electrochemistry		Global electrochemical current and potential	Y	10^−5^ -10^0^ m	10^−4^ - 10^−1^ s	
Scanning vibrating electrode technique	SVET	Localised cathodic and anodic reaction mapping	Y	10^−4^ m	10^3^ s	
Scanning Kelvin probe	SKP	Metallic work functions, Volta potential	N	10^−6^ m	10^3^ s	Limited lateral resolution
Scanning Kelvin probe force microscopy	SKPFM	Metallic work functions, Volta potential	N	10^−9^ m	10^3^–10^4^ s	
Electrochemical micro-capillary		Phase electrochemistry, micro-galvanic coupling	Y	10^−5^ - 10^−3^ m	10^−2^ - 10^3^ s	
Electrochemical noise	EN	Localised reaction transients	Y	10^−4^ - 10^−1^ m	10^−3^ - 10^5^ s	
Scanning electrochemical microscopy	SECM	Localised cathodic and anodic reaction mapping	Y	10^−9^ m	10^−2^ s	
Acoustic emission	AE	Energetic rupture event timing	Y	10^−3^ - 10^−1^ m	10^−3^ - 10^5^ s	
Beamline spectroscopy and diffraction		Structure, composition, imaging	Y	10^−11^ - 10^−1^ m	10^−6^ - 10^4^ s	
Small-scale tensile		Mechanical properties, event timing and progression	Y	10^−3^ - 10^−1^ m	10^3^ s	
Small disc/small punch		Mechanical properties, event timing and progression	Y	10^−4^ -10^−2^ m	10^3^–10^5^ s	
Micro-scale mechanical		Mechanical properties, event timing and location	Y	10^−6^ - 10^−5^ m	10^0^–10^5^ s	

**Figure 5:**
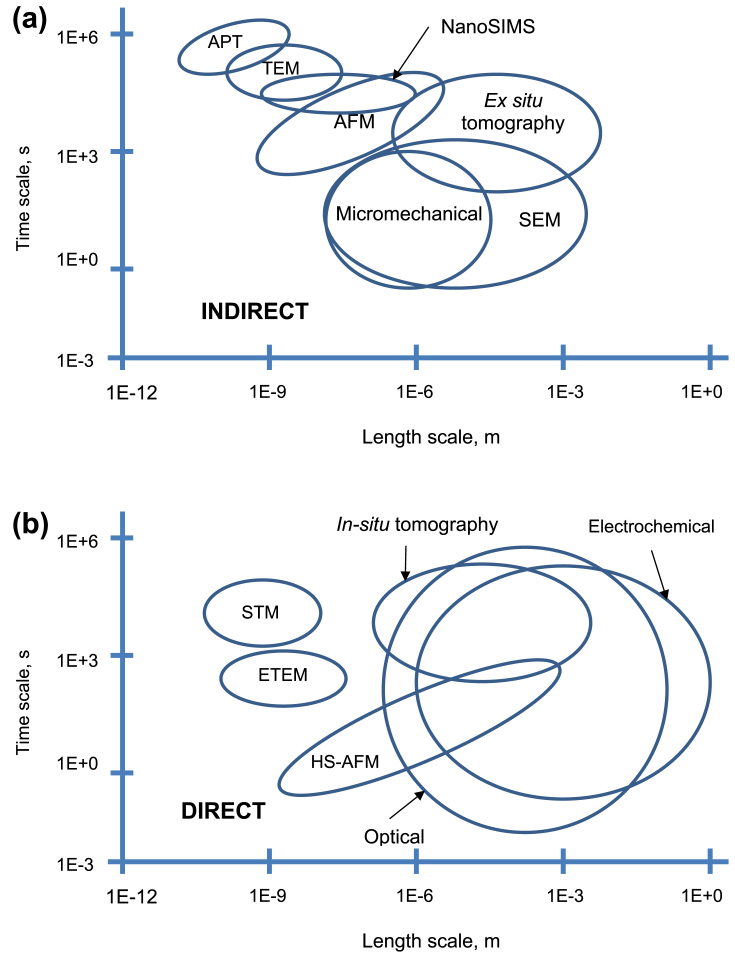
Schematic overview of selected techniques^5^ relevant to the study of SCC: (a) indirect; (b) direct.

**Figure 6:**
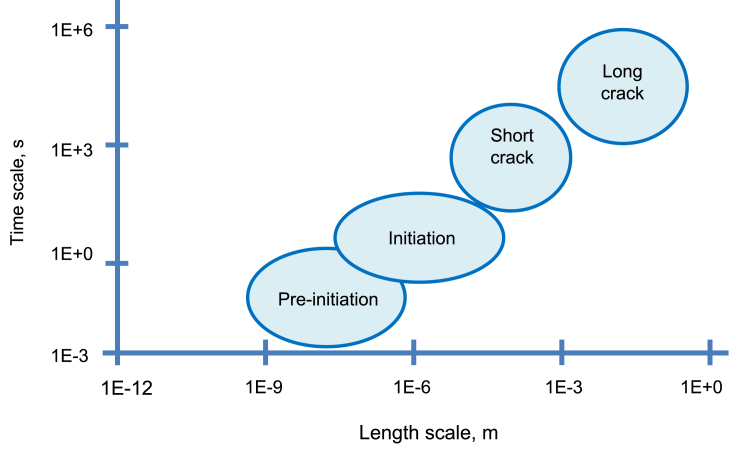
Representation of EAC/SCC stages according to approximate length- and time-scales.

**Figure 7:**
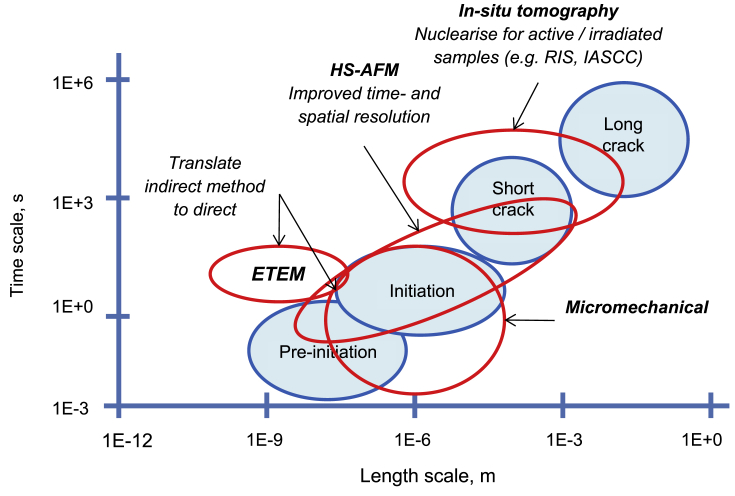
Opportunities where selected techniques^6^ could be applied in micro-scale testing of EAC/SCC.

Figure 5 shows a selection of the techniques listed in this paper as indirect (Figure 5a) and direct methods (Figure 5b), and the corresponding time and length resolution for each. Within indirect techniques, atom probe tomography (APT) and transmission electron microscopy (TEM) allow evaluation of the smallest features, while (*ex situ*) tomography and scanning electron microscopy (SEM) allow larger features to be observed. Nanoscale secondary ion mass spectrometry (NanoSIMS) offers high spatial resolution (<100 nm) chemical composition with high sensitivity (ppm). Micro-mechanical techniques allow the study of features somewhere inbetween the large and small. Some direct techniques can also measure to the small length-scale that is offered by indirect methods, such as environmental TEM (ETEM). High-speed atomic force microscopy (HS-AFM) allows small features to be measured directly, whilst electrochemical and optical techniques allow only the largest features to be evaluated. Figure 6 shows the applicability of these techniques to provide detail on the different EAC processes. As summarised in Figure 7, only using a combination of multiple techniques across multiple length-scales allows pre-initiation, initiation, short crack and long crack EAC to be evaluated. Serial sectioning and XCT are capable of providing information on short cracks, HS-AFM/micro-mechanical approaches yield information on initiation and pre-initiation, and ETEM gives pre-initiation information.

### 3.2 Surface methods

Methods that can be applied to free surfaces may be applied in general to specimens in one of two forms: (i) the surface, subject to an environment and stress, to provide a correlation between local microstructure (location) and electrochemical activity leading to crack initiation; and (ii) a section normal to a surface, to give sub-surface detail about initiation, short crack formation and crack growth in relation to microstructure and local corrosion within a nucleating site, such as a pit or crack.

#### 3.2.1 Optical microscopy

Optical techniques can give visual information about changes on the surface of a material during EAC tests to provide a multifaceted perspective of the process. This microscopy can be conducted either *in situ*, or *ex situ* following EAC. Thus, the technique can be classed as being capable of both direct and indirect measurement. Ultimately, the length resolution of optical microscopy is constrained by the wavelength of light to be ~0.3 μm. Furthermore, optical techniques can have issues with depth-of-field, which decreases at higher magnifications. Some of the limits, *e.g.* depth-of-field, can be overcome by using confocal techniques. Confocal microscopes use a focused laser to raster scan the surface, with the laser spot size tailored to meet the required resolution, and, therefore, can acheive increased resolution and depth-of-field [Alford et al., 1982; Wilson, 1994].

**Figure 8:**
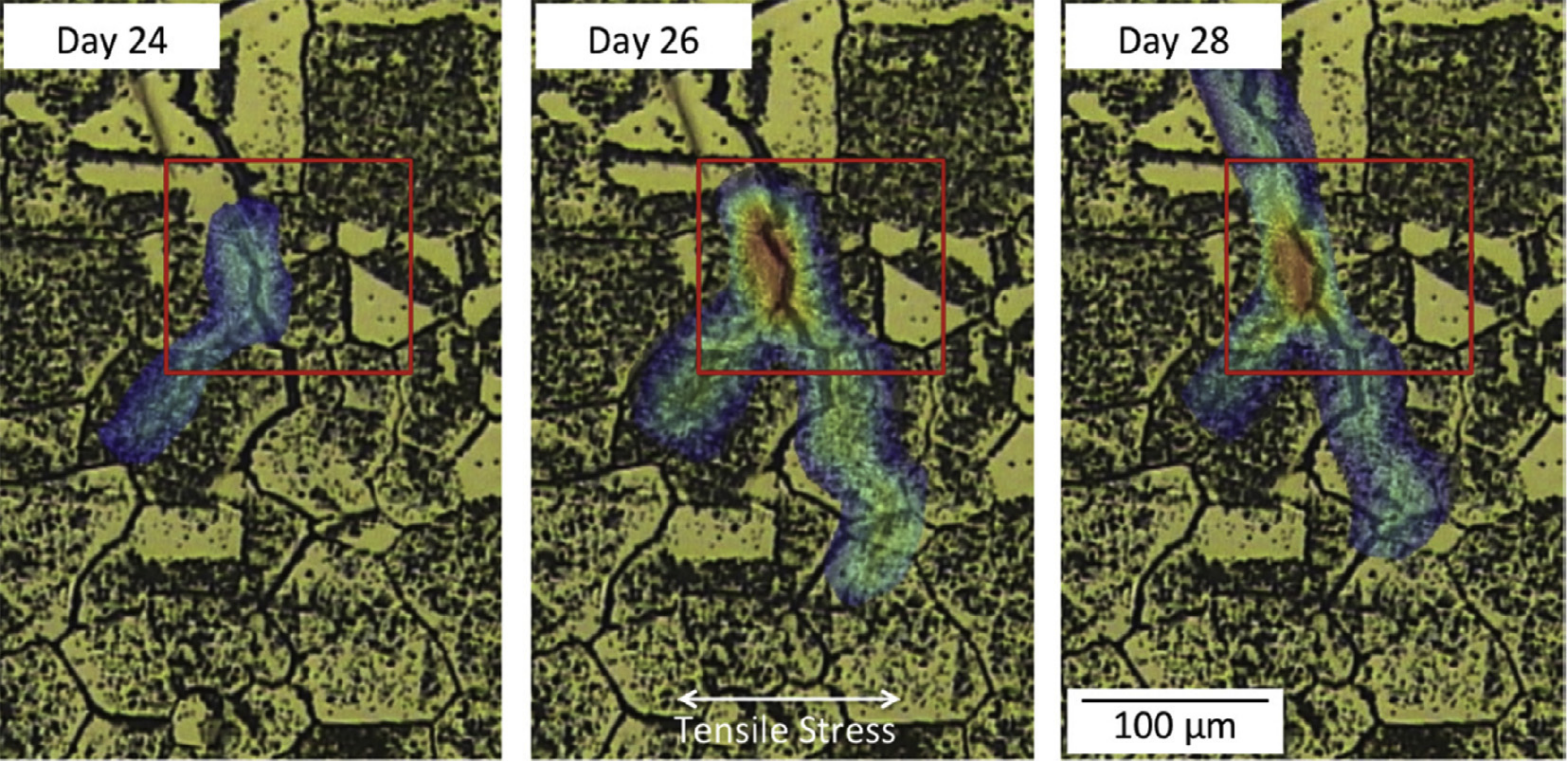
*In situ* observation of sensitised 304 stainless steel in a windowed autoclave, exposed to high-temperature, high-purity, oxygenated water (250 °C; 1 ppm O_2_; 50 atm). The colour overlay relates to the level of strain obtained by digital image corellation, from the crack opening displacement, with high-strain represented by red, and low strain dark blue. From Stratulat et al. [2014].^7^

For *in situ* time-lapse microscopy measurement, either a liquid flow cell [Sullivan et al., 2011] or a simple waterproof shroud can be used [Sullivan et al., 2015]. These allow microstructural corrosion mechanisms to be observed within the corrosive liquid environment. For *ex situ* observation, following optical visualisation of a system undergoing a specific test, the captured images can be analysed using digital image correlation (DIC) [Kovac et al., 2010] to track changes in physical features, such as surface defects. Stratulat et al., [2014] used a windowed autoclave and DIC to track sub-micrometre displacements (and, hence, strain), allowing IGSCC propagation to be monitored in a hot water environment as a result of crack opening displacement (Figure 8).

**Figure 9:**
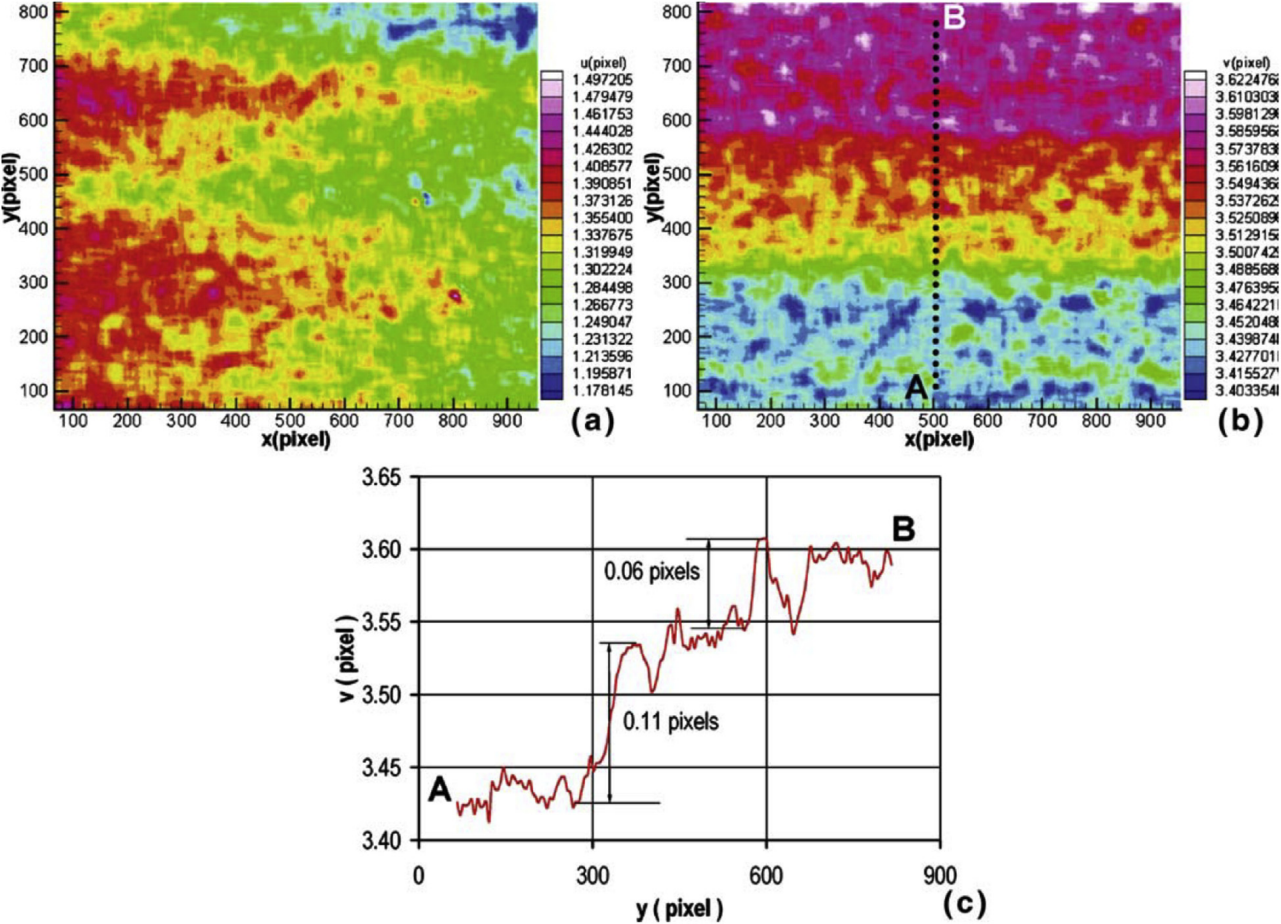
Displacement fields at 5000x for gold coated on aluminium using a FEI Quanta-200 SEM: (a) horizontal; (b) vertical. The scale bar shows the change in displacement in pixels. From Sutton et al. [2007].^8^

**Figure 10:**
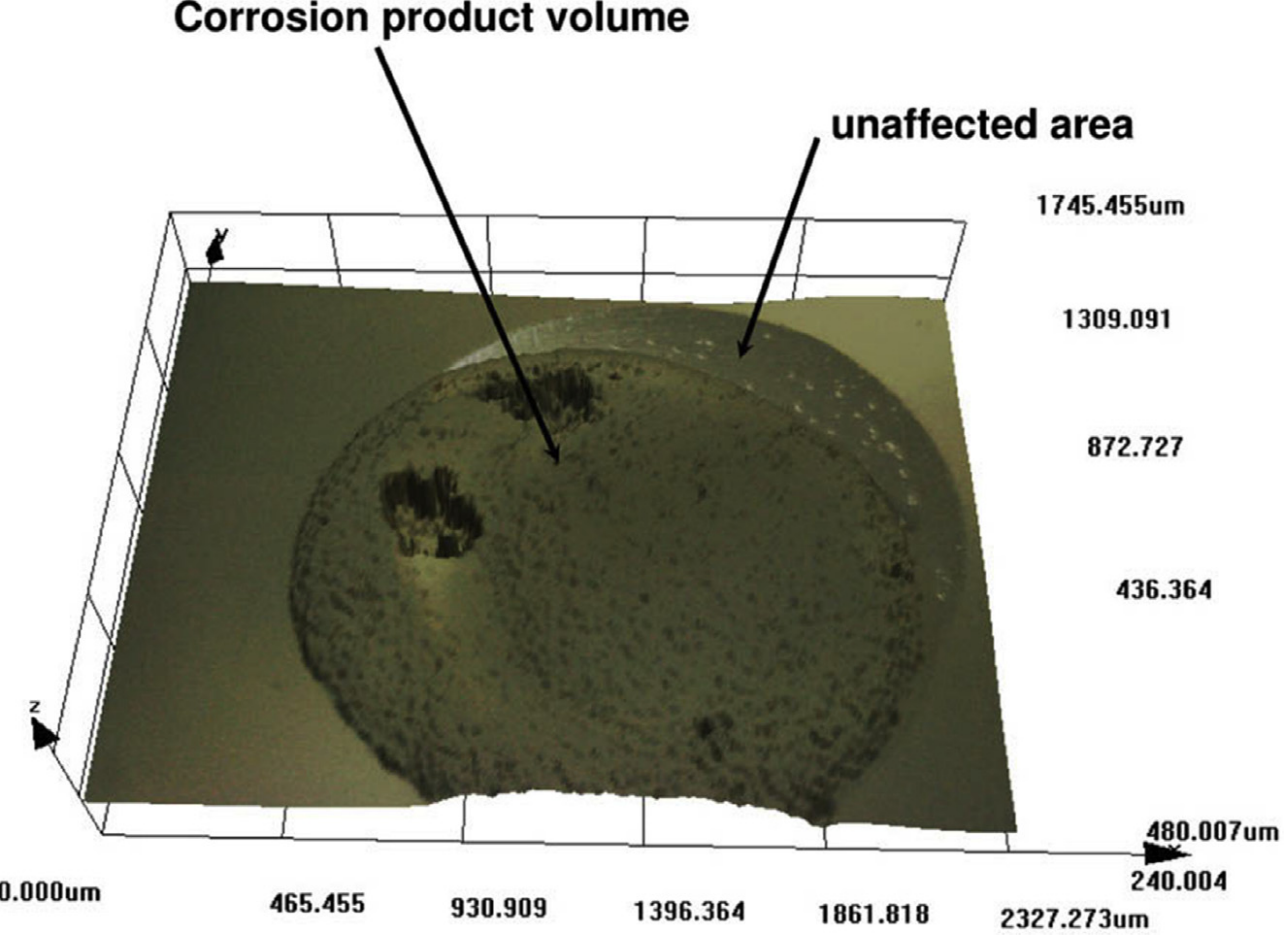
Duplex stainless steel under galvanodynamic control studied *in situ*, using confocal imaging. The x, y, z axes represent length in units of micrometres. From Leiva-Garcia et al. [2010].^9^

Sutton et al. [2007] showed that, if a pattern is present on the specimen, DIC can be conducted on SEM images. Successful DIC for such images is caveated by the fact that distortion and instability can cause small shifts in image position due to the beam raster scan. These shifts are more pronounced at higher magnifications (>5000x), and start to introduce error in regard to local strain measurement. The displacement in pixels within the horizontal and vertical directions for gold coated on aluminium (which created a random speckle pattern) and subsequently imaged within an SEM at 5000x magnification is shown in Figure 9. Here a 0.1 pixel step change in the vertical direction at 5000x was equivalent to a 5 nm displacement. The use of confocal techniques can help alleviate depth-of-field and resolution issues. Imaging throughout the micrometre range can be achieved with this technique and high-density, charge-coupled device cameras allow multiple frames-per-second capture rates. There are practical considerations affecting the use of such high-magnification apparatus in conjunction with a liquid cell and loaded specimen that must first be considered. Leiva-Garcia et al. [2010] used confocal microscopy (Figure 10), in conjunction with a novel minicell, to study *in situ* corrosion initiation on a duplex stainless steel under galvanodynamic control. Use of the confocal technique allowed details, such as the corrosion product, to be resolved in the z-direction.

Optical microscopy can help evaluation of EAC *in situ*. However, the technique is limited by its resolution, and the depth-of-field at high magnifications. Depth-of-field is particularly important if the material and the crack are to be in focus when stressed at magnifications which can give meaningful information.

#### 3.2.2 Electron microscopy

##### 3.2.2.1 Ex situ electron microscopy

SEM is ubiquitously employed for nano-scale resolution imaging in almost every area of materials science, not least corrosion studies (Breimesser et al., 2012; King et al., 2008; Kovac et al., 2010]. Imaging is typically undertaken post-exposure to observe final surface features, such as failed grain boundaries, as well as prior to corrosion, so that the surface may be characterised by this indirect measurement. These scanning instruments are usually fitted with a range of detectors so that, for example, chemical information can be obtained from energy dispersive X-ray spectroscopy (EDS) and quantitative crystallographic data *via* electron backscatter diffraction (EBSD) instrumentation. A considerable amount of information about a surface can be obtained: grain orientation distribution; grain boundary misorientation; surface texture (the average preferred orientation); grain boundary and phase distribution; residual strain variations; and state of strain within the material.

**Figure 11:**
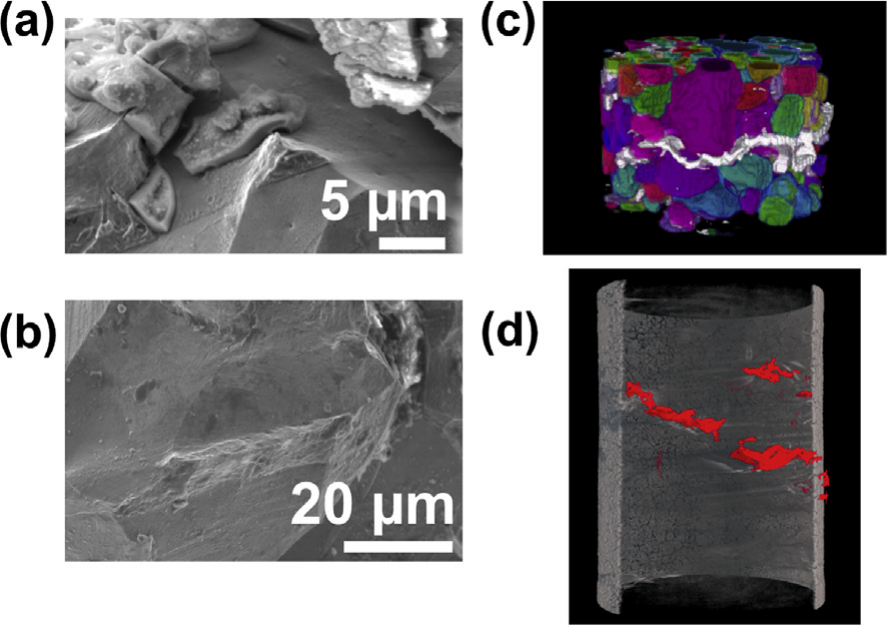
(a, b) SEM of the fracture surface following the experiment, showing what is likely ductile failure of the twinned regions; (c) X-ray computed tomography combined with diffraction computed tomography (DCT), the white regions show the crack paths obtained from computed tomography (CT), as compared to the DCT data showing the grain shapes (coloured); (d) *In situ* CT of a stainless steel wire undergoing EAC, the red areas denote the crack path.^10^

EBSD can identify plastic zones and determine residual plastic strain in a specimen [Humphreys, 2004; King et al., 2008; Maitland and Sitzman, 2007]. This information is valuable when analysing SCC crack initiation [Othon and Morra, 2006]. Although SEM analysis is straightforward for electrically-conductive specimens, materials which oxidise can prove challenging to image due to charge accumulation, with the same issue applying to corrosion products. For non-conductive specimens, sputter-coating can be undertaken with a conductive metal, such as gold. Alternatively, the charging problem can be overcome with the use of variable pressure modes, as found in environmental SEMs (ESEMs), whereby both vacuum and gas are used in the chamber (see Section 3.2.2.2). Figure 11 shows work by King et al. [2008] on the IGSCC of a susceptible specimen, sensitised stainless steel. SEM was utilised for post-exposure characterisation of the fracture surface, alongside *in situ* XCT and more advanced X-ray diffraction contrast tomography (DCT) to identify the types of grain boundaries resistant to IGSCC.

TEM is capable of nano-scale crystallographic characterisation of materials [Wang, 2000] and, as such, can be used to provide insight into SCC [Huang and Titchmarsh, 2006], including the *in situ* reaction of a specimen exposed to an electric field, mechanical stress or annealing [Wang, 2000]. Specimen preparation is substantially more time consuming than SEM as very thin sections must be produced, often by focused ion beam (FIB) milling or electropolishing. As with SEM, TEM is an indirect measurement, given that imaging and analysis is conducted *ex situ*. From Flewitt and Wild [2017].

**Figure 12:**
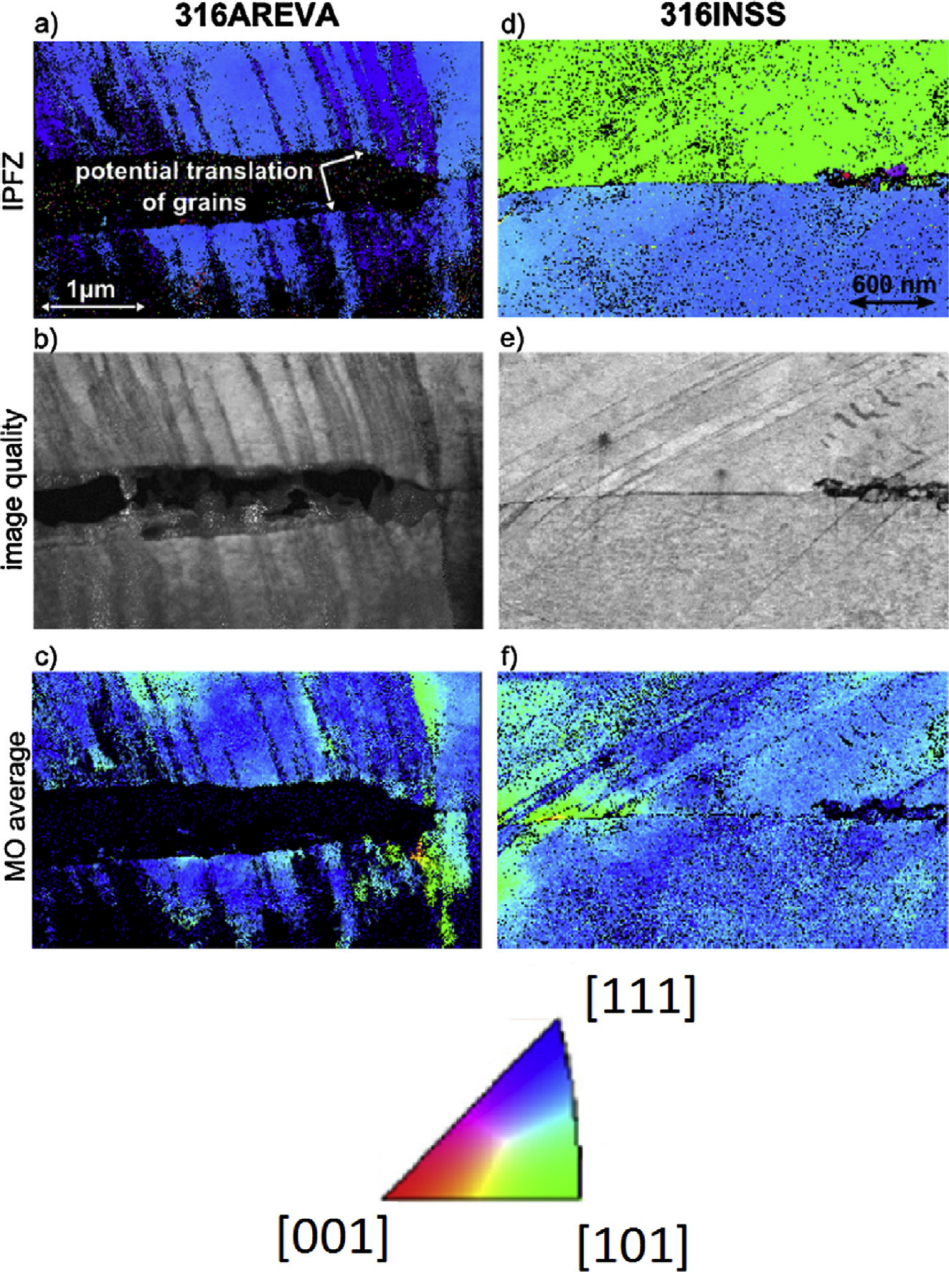
Transmission EBSD (t-EBSD)/ transmission Kikuchi diffraction (TKD) maps of stainless steels exposed to PWR chemistry conditions: 316AREVA (a) to (c) relates to hydrogenated conditions and 316INSS (d) to (f) to shutdown (oxygenated) PWR conditions. Different crystal orientations are shown at the top of the figure (inverse pole figure, z-direction), relating false colour maps (a) and (d). In (b) and (e), the differences in contrast relate to differences in crystal orientation or crystallographic defects. (c) and (f) show the average misorientation (MO) with respect to surrounding pixels; dark blue represents low MO, and green, yellow and finally red represent successively higher average MO. From Meisnar et al. [2015a].^11^

EBSD has been employed with thin-section TEM specimens to study SCC crack tips in two type 316 stainless steels [Meisnar et al., 2015a]. One exposed to hydrogenated PWR operating conditions (316INSS) and the other to oxygenated PWR shutdown chemistry conditions (316AREVA), both under constant load within an autoclave, and exhibiting SCC. Specimens were removed from the autoclave, cross-sectioned, ground, polished, crack tips located, crack tip removed using FIB, mounted onto TEM grids, thinned to the required transparency and, finally, plasma cleaned. In the example of Figure 12, the results indicated that for the specimen in oxygenated conditions (316AREVA) shear strain may have been involved in the opening of the crack. The average misorientation (MO) maps provided evidence of this claim. In image Figure 12(c), the lower grain had a higher average MO compared to the upper grain, which would cause a shear stain between the two boundaries. In the example shown in Figure 12(f) (hydrogenated conditions), the average MO is constrained to areas where defects were present, such as the slip bands. The main constraint on conventional electron microscopy is that it does not allow for *in situ* characterisation under the high-vacuum conditions normally employed.

It should be recognised that these techniques adopt specimens that are unloaded, and therefore there is potential for crack closure. In addition, thin TEM foils used for evaluating cracks will have relaxed any residual stresses/strains.

##### 3.2.2.2 In situ environmental electron microscopy

**Figure 13:**
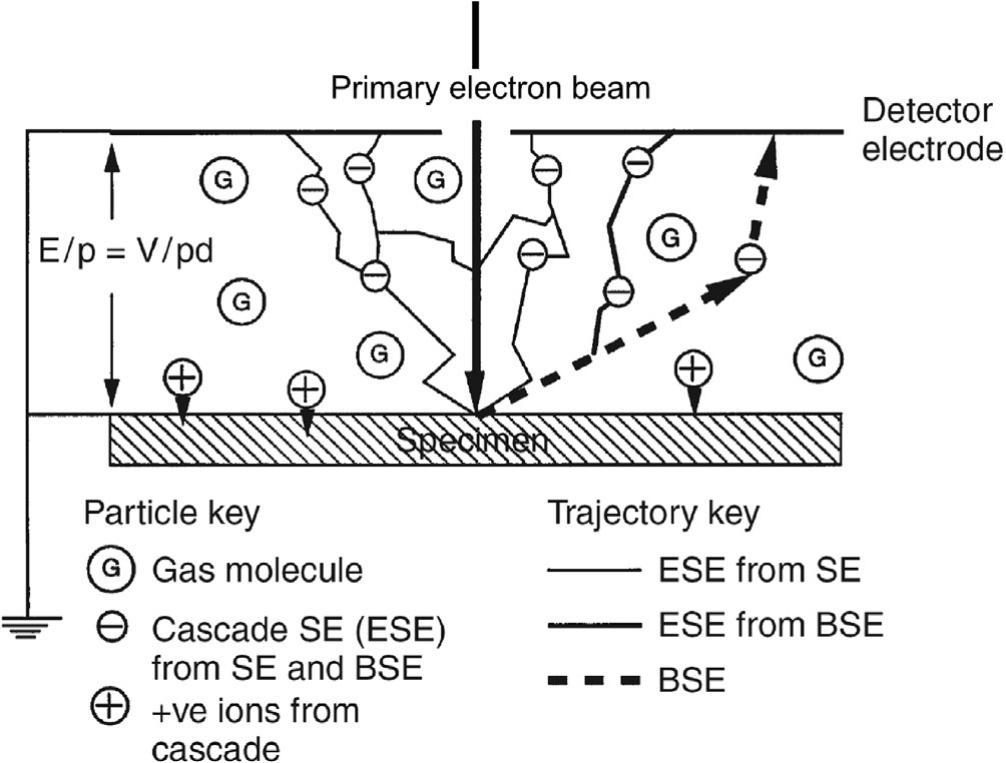
An illustration of how ESEM uses a detector electrode, earthed specimen and positive electrode bias, which together act as a capacitor. The gas between the parallel plates acts as a dielectric. From Meredith et al. [1996].^12^

ESEM allows imaging with a nominal gas pressure within the instrument chamber [Meredith et al., 1996; Moncrieff et al., 1978; Moncrieff et al., 1979; Neal and Mills, 1980], including water vapour, which may be controlled and increased to saturation. Figure 13 shows a schematic of an ESEM's gaseous parallel plate capacitor arrangement. By earthing the specimen, bringing the scanning electron beam through an aperture in the top plate and imposing a positive potential bias, the gas acts as a dielectric. This causes secondary and backscattered electrons to be attracted to the positively-charged top plate. Subject to the inter-plate separation being smaller than the mean free path of the ejected electrons, these electrons may cause subsequent cascade amplification [Meredith et al., 1996]. This facilitates characterisation of hydrated specimens, and humidity cycling to allow reactions to occur within a liquid film on the specimen surface under evaluation, although the technique is constrained by the opacity of liquid water to electrons.

**Figure 14:**
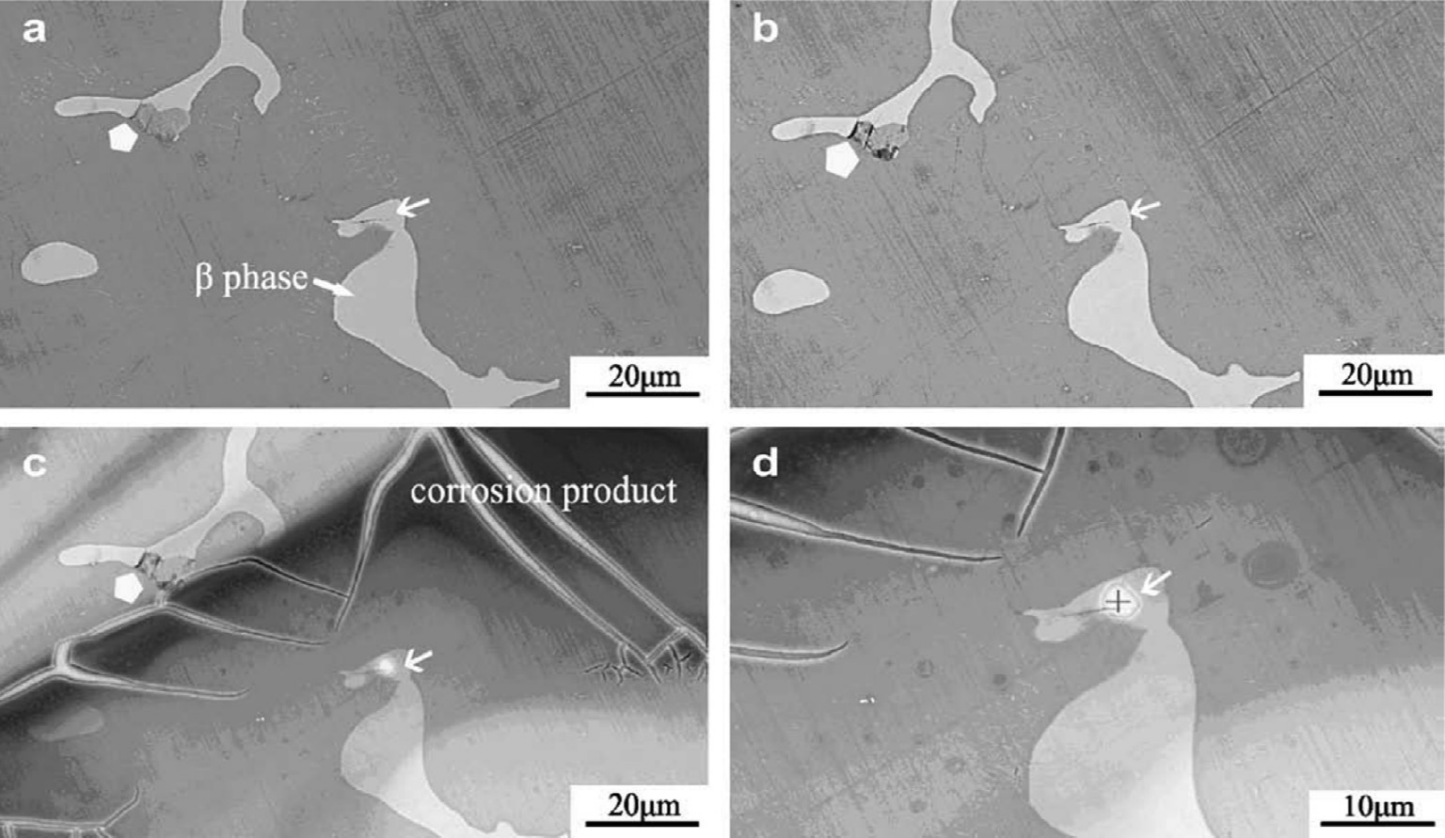
Observation of SCC propagation in AZ91 cast magnesium alloy exposed to a salt solution using backscattered electrons within an ESEM: (a) 0 humidity cycles; (b) 3 cycles; (c) 6 cycles; (d) higher magnification image of (c). From Chen et al. [2008].^13^

The opacity of liquid water requires humidity cycling for incremental reaction observations [Chen et al., 2008]. In the example by Chen et al. [2008], deionised water vapour was unsuitable to initiate SCC on (cast) AZ91 magnesium alloy, so salt particulates were introduced onto the specimen surface prior to insertion into the ESEM chamber. As the humidity inside the chamber increased, the salt deliquesced, forming aqueous brine and providing an environment sufficiently aggressive for SCC to initiate, as shown in Figure 14. The humidity was cycled, with 1 h of wetting time (6.5 Torr) and 10 min of drying time (2.1 Torr). ESEM provides a direct measurement as it has similar fast imaging to SEM, but experiments can be conducted *in situ*.

A high-voltage ETEM (1 MeV) with an environmental cell was used by Tanaka et al. [2014] for imaging atomic arrangements during oxidation of a metal surface exposed to oxygen and the fracture of multicomponent specimens under hydrogen. Typically, medium acceleration voltages are up to ~400 keV for conventional systems [Flewitt and Wild, 2017]. Often what is required is techniques (such as TEM) to characterise specific regions susceptible to EAC (grain boundaries, precepitates, regions ahead of crack tips); FIB allows such site specific regions to be prepared. However it is also important to characterise representative areas hence a general issue which applies to TEM, as with most very-small-scale characterisation techniques, is if the specimens are representative of the bulk material microstructure and reaction kinetics. One approach adopted to explore these limitations is to sample a large number of individual sites to provide statistically-representative data. Since the number of specimens has to be large, this comes at a significant cost in terms of machine time and specimen preparation, so is not always achieved. However, the aim of a particular technique is often to locate a specific feature of interest (such as a crack tip) and characterise the local conditions. However, even for such a selection it is possible to overlook variations or, conversely, attribute significance to spurious features.

SEM typically provides maximum resolution in the region of tens of nanometres. Imaging times are relatively fast, with even high-quality images requiring less than a minute. TEM can provide images with atomic resolution, but has a somewhat slower imaging acquisition rate. Specimen preparation for TEM is very time-consuming. One attractive aspect of ETEM is that the course of a reaction may be tracked dynamically, compared to the requirement to prepare a sequence of specimens showing different levels of process progression.

In summary, SEM offers high-throughput, high-magnification imaging with relatively straightforward preparation, making it a valuable tool for evaluating EAC. ESEM provides the ability to characterise the specimen *in situ,* providing the possibility to image cracks propagating under SEM-resolution conditions (~1–20 nm). This is akin to time-lapse microscopy, but at higher magnification and with a far better depth-of-field. Since ETEMs are not commonplace, it is primarily *ex situ* TEM that is adopted to allow high-resolution imaging and analysis of EAC crack tips, post-testing.

#### 3.2.3 Focused ion beam techniques

**Figure 15:**
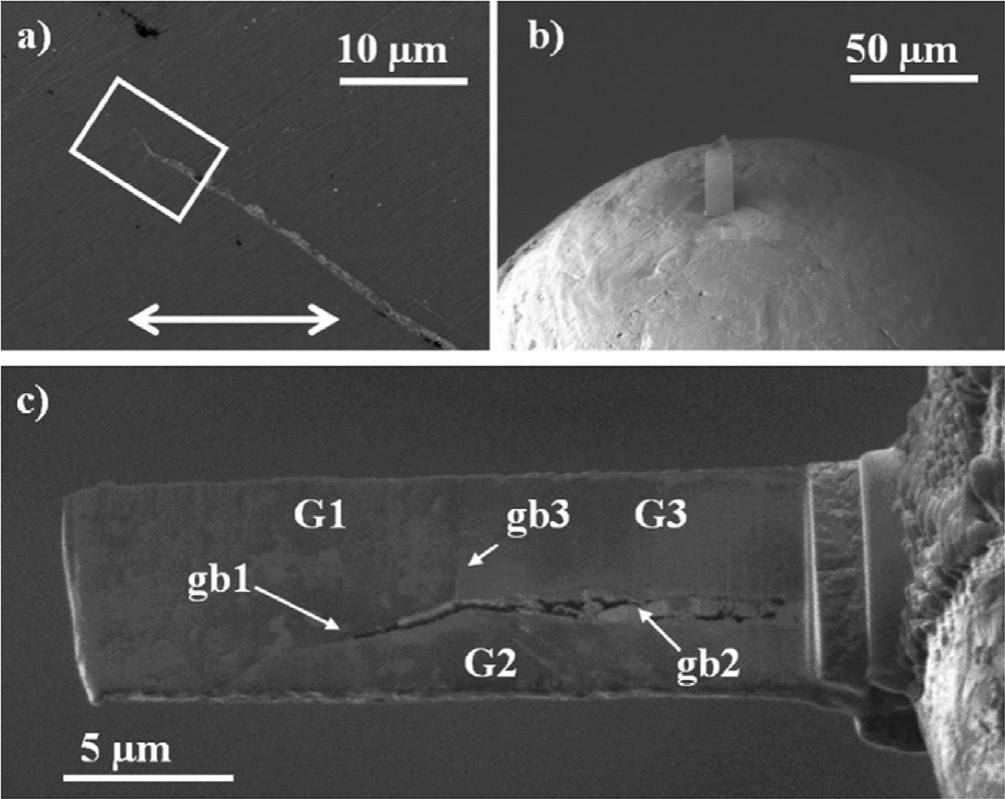
FIB-SEM images of stainless steel (304) exposed to simulated PWR conditions, from Lozano-Perez et al. [2011]^14^: (a) cross-sectional view of SCC crack (the white box represents the area where tomography was undertaken); (b) sample of the crack tip fabricated through FIB in plan-view; (c) plan view of the crack sample prior to thinning for TEM showing grains, grain boundaries and oxidised grain boundaries.

**Figure 16:**
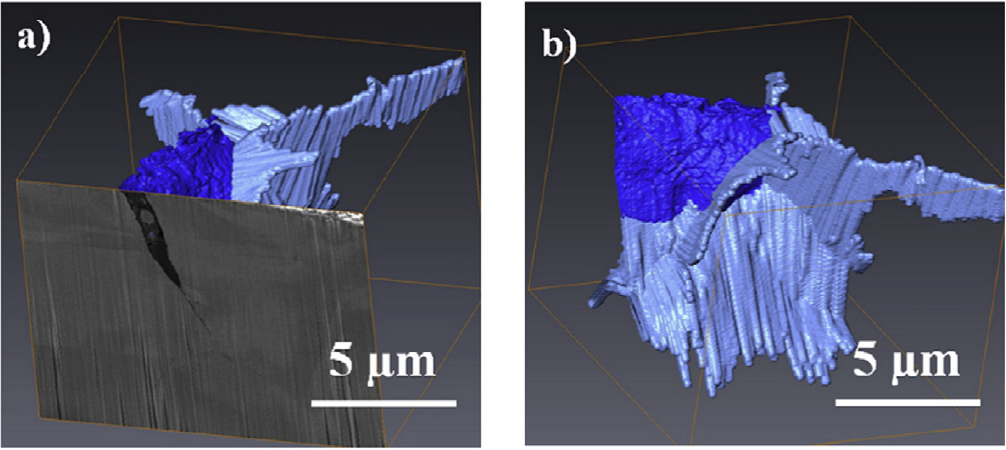
Example of 3D-FIB serial sectioning undertaken on nickel base Alloy 600, exposed to PWR conditions, from Lozano-Perez et al. [2011]^15^: (a) SEM image of the crack, together with a 3D reconstruction model; (b) 3D-FIB model; dark blue represents the open crack while light blue represents oxidised grain boundaries.

A FIB workstation is a versatile instrument, can be used to fabricate test specimens (such as micro-cantilevers, as discussed in Section 3.6.3), and can utilise high-resolution, serial section tomography to provide 3D images (Figures 15 and 16) [Lozano-Perez et al., 2011].

In general, systems use gallium ions to mill specimens and provide images of surfaces. In current generation dualbeam instruments, the ion beam capability is paired with electron imaging, allowing complementary use of techniques. This can be extended by incorporation of EDS to provide elemental characterisation. SCC cracks can be sectioned at specific locations on the specimen surface, without disturbing either the rest of the crack or any adjacent cracks that may be present [Wang et al., 1999]. Typically, for ion milling a beam current of ~90 nA can be used to form a trench with accuracy down to tens of nanometres [Wang et al., 1999]. By tilting the specimen with respect to the incident ion beam, the trench can be imaged to allow for crack depth measurements and observation of the crack profile. High-resolution tomography involves iterative sectioning combined with image reconstruction to characterise specific nano length-scale features (Section 3.3.2.1).

Using a combination of these approaches, it is possible to identify dominant cracking modes (IGSCC or TGSCC), obtain information on the morphology of crack tips and map associated microstructural features, the positions of inclusions and corrosion products. Grain boundaries are imaged clearly with FIB from the enhanced grain contrast. FIB has been utilised in a number of SCC investigations to evaluate crack profiles, plastic deformation and crack development in various materials, such as stainless steels and nickel alloys [Li et al., 2008; Lozano-Perez et al., 2011; Wang et al., 1999]. The resolution of the technique for imaging is generally comparable to high-resolution SEM. Imaging times are rapid, although milling (*e.g.* to undertake FIB tomography) can be time-consuming because the milling response is dependent on the specific material. Another consideration is that high-resolution serial sectioning by FIB is not without error, for example, the milling and spacing is not always uniform, and in some alloys phase transformation can occur. As such, these uncertainties should be considered during image reconstruction.

#### 3.2.4 Scanning probe microscopy

##### 3.2.4.1 Atomic force microscopy

**Figure 17:**
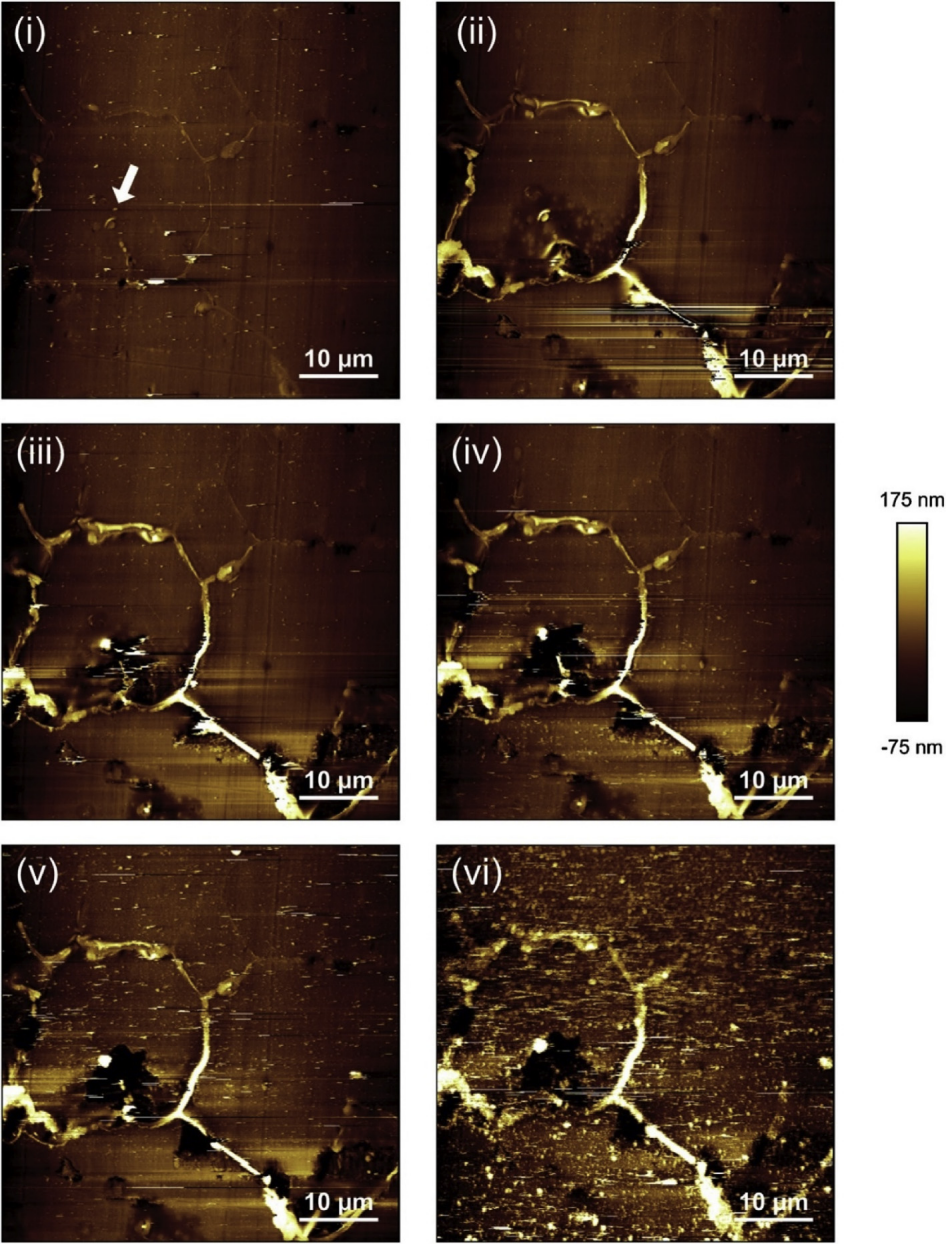
*In situ* electrochemical AFM images of a thermally-sensitised austenitic stainless steel exposed to 1 mol dm^−3^ NaCl (aq): (i) 30 min; (ii) 1 h; (iii) 1 h 30 min, (iv) 2 h, (v) 2 h 30 min, (vi) 4 h. The arrow in (i) shows the location of what is potentially a niobium carbide precipitate. The scale bar shows relative height in nm. From Clark et al. [2020].^16^

Atomic force microscopy (AFM) is one of a group of techniques known collectively as scanning probe microscopy (SPM) [Flewitt and Wild, 2017; Payton et al., 2016]. In comparison to other visualisation techniques, AFM has the capability to provide high-resolution topographic images of surfaces and a measure of mechanical properties at nanometre-scales in various gaseous, liquid and vacuum environments [Liao et al., 2013; Payton et al., 2012; Schitter et al., 2007]. AFM may be undertaken as an *in situ* technique, as shown in the research by Clark et al. [2020]. These workers adopted contact mode AFM to study the corroding surface of a thermally-sensitised austenitic stainless steel (20Cr–25Ni–Nb) used as nuclear fuel cladding in the UK's AGRs. It was possible to image corrosion processes on the micrometre length-scale using contact mode. The corrosion was highly localised, which the authors referred to as ‘intergranular pitting corrosion’ since pits formed at distinct grain boundary locations, as can be seen in Figure 17. Due to the high resolution offered by the technique, it was possible to gain insight to the role that niobium carbide precipitates play in the corrosion process. Here, a niobium carbide precipitate is largely unaffected by corrosion, with the outer matrix and grain boundary areas affected by intergranular pitting corrosion in preference. This *in situ* electrochemical AFM technique does have drawbacks, however, largely due to the time taken for the instrument to collect data. Particular developments of the AFM method are described in the following Sections. Typically, as the conventional technique has a relatively slow imaging time this method achieves best results when used for post-exposure characterisation. Therefore, for the purposes of this appraisal, the undeveloped method is classed as an indirect observation.

Typically, AFM probe tip radii are between 10 - 60 nm; and sharp AFM tips are one factor which is required to provide high-resolution images. However, AFM tips are fragile and mis-shaped tips lead to errors in measurement. The AFM tip may be blunted or damaged during measurement which can lead to artefacts such as blurred images and ghosting [Eaton and West, 2010]. The fact that AFM is not limited to low-pressure, or vacuum environments offers advantages over other techniques [Payton et al., 2016], leading to a multitude of applications in materials, surface and biological sciences. Moreover, it is a versatile and significant tool at the nano length-scale [Humphris et al., 2005; Payton et al., 2012].

**Figure 18:**
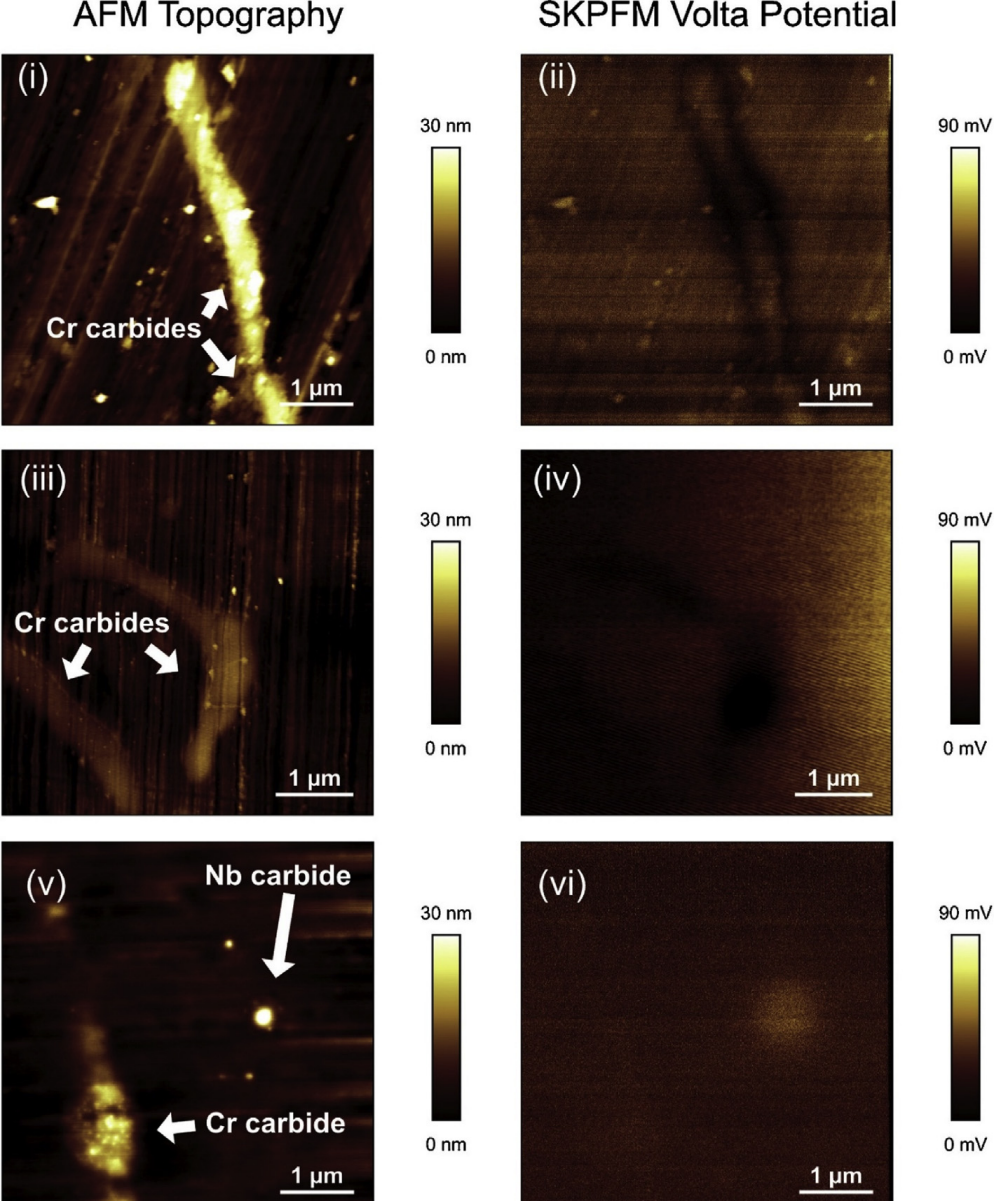
AFM topography and SKPFM Volta potential maps of 20Cr–25Ni–Nb austenitic stainless steel. (i) sensitised grain boundary Cr carbide topography map, (ii) corresponding Volta potential map, (iii) magnified topography image of grain boundary Cr carbide, (iv) corresponding Volta potential map, (v) topography map showing Cr and niobium carbide inclusions, (vi) corresponding Volta potential map. Scale bars show relative height for AFM images (i, ii, iii) and Volta potential for SKPFM maps (ii, iv, vi). Volta potential colours are inverted (darker = less noble). From Clark et al. [2020].^17^

A specific type of *ex situ* SPM which has been utilised in various corrosion studies is the micro-scale scanning Kelvin probe force microscope (SKPFM), which has similarities to macro-scale scanning Kelvin probe (SKP); comparisons between the two have been discussed [Cook et al., 2012b; Rohwerder and Turcu, 2007]. SKP is discussed further (Section 3.4.1.2). The oscillating AFM tip performs a topography measurement, is subsequently lifted by a user set distance (typically 100 nm) and then rescanned across the same surface. This provides an indirect measurement and cannot be conducted with an electrolyte present. SKPFM measures the Volta potential difference, which is related to the work function of the surface and precipitates interrogated [Riva et al., 2016]. The Volta potential difference gives information on the nobility of different metals and/or phases; for instance, Clark et al., [2020] used SKPFM to evaluate the thermodynamic susceptibility for niobium carbide inclusions and chromium carbide precipitates formed at grain boundaries to undergo localised corrosion with respect to an austenite stainless steel matrix (Figure 18). The lighter colours in the AFM maps correspond to peaks, whereas in the SKPFM maps these correspond to areas of lower thermodynamic corrosion susceptibility. Darker colours represent troughs in the AFM maps and areas of less noble in the SKPFM maps (*i.e.* as per convention polarity has been reversed). An AFM topography map (Figure 18(i)) shows grain boundaries decorated with chromium carbides following a thermal sensitisation process; the corresponding AFM Volta potential map is shown in Figure 18(ii). Figure 18(iii, iv) show the same region, but at higher magnification. Figure 18(v) shows the AFM topography map of a niobium carbide precipitate near a grain boundary for the same alloy but without the thermal senitisation and Figure 18(vi) presents the corresponding Volta potential map. Clark et al. [2020] showed that chromium carbide precipitates which decorated the grain boundaries after a thermal sensitisation treatment were less noble with respect to the austenite matrix. On the other hand, niobium carbide precipitates (Figure 18(vi) image shown for non-sensitised sample, same alloy) were noble compared to the matrix; intergranular dissolution would be expected to occur in the sensitised alloy, and in regions adjacent to the niobium carbide precipitates. Therefore, this technique informs the user if particular phases are susceptible to corrosion on the basis of thermodynamics.

Conventional AFM techniques generally have spatial resolutions in the nanometre range, though with relatively small imaging areas (typically <100 μm^2^) and slow imaging speeds (<1 Hz). Thus, for SCC AFM is best reserved for post-exposure analysis (topography). Also SKPFM is an indirect technique and provides a measure of Volta potential differences, for example between the matrix, a crack tip and precipitates.

##### 3.2.4.2 High-speed atomic force microscopy

HS-AFM is a recent development which significantly reduces the imaging time compared to conventional AFM, whilst maintaining nanometre lateral resolution and subatomic height resolution. It is uniquely capable of directly imaging nano-scale dynamic processes under aqueous conditions at sub-second temporal resolution (typically 2–10 fps, fastest 1000 fps [Picco et al., 2006]) [Ando, 2013; Ando et al., 2012; Eghiaian et al., 2014; Katan and Dekker, 2011; Liao et al., 2013; Payton et al., 2016]. This has enabled extensive new research in numerous scientific disciplines [Mikheikin et al., 2017; Pyne et al., 2009] and classifies HS-AFM as a direct technique, distinct from conventional AFM by allowing for corrosion studies. The faster imaging speeds, seconds as opposed to tens of minutes [Laferrere et al., 2017] translate directly into larger scan areas with automated wide area data collection and stitching routines [Klapetek et al., 2015; Payton et al., 2016]. Currently, 250 × 250 μm square areas are stitched routinely, with a resolution of 5 x 5 nm. This higher throughput allows for the collection of statistically-significant data sets.

Measurement modes other than simple topography have been demonstrated, allowed for by the contact mode cantilever arrangement and high-bandwidth data handling of such a system. These have included contact resonance for micro-stiffness [Hurley et al., 2007], electrical conductivity and thermal conductivity [Minne et al., 1998; Payton et al., 2016]. Further capabilities are being developed, allowing for measurements to be performed within an environmental chamber either at a specified humidity or under different gaseous environments.

**Figure 19:**
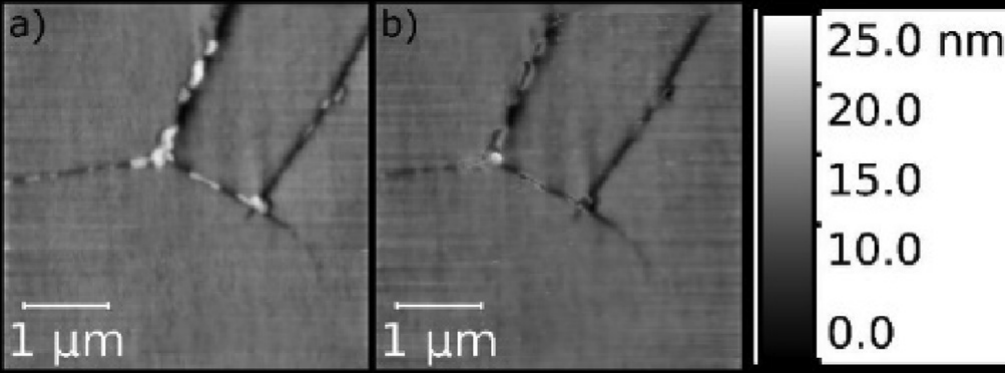
*In situ* HS-AFM images of sensitised 20Cr–25Ni–Nb stainless steel grain boundaries: (a) before; (b) after +500 mV (Vs. Pt) polarisation in 5 mg.L^−1^ [Cl^−^]. The images show dissolution of grain boundary precipitates, thought to be chromium carbides. The scale bar shows the height in units of nm. From [Laferrere et al., 2017].^18^

Laferrere et al. [2017] reported experiments using contact mode HS-AFM (Figure 19) to image nano-scale corrosion in thermally-sensitised 20Cr–25Ni–Nb stabilised stainless steel. The work was conducted within a micro-electrochemical cell, with the aim of better understanding the role of microstructural segregations in corrosion. Further examples of the use of SPM in electrochemistry can be found in Fu and Rudnev [2017] and Moore et al. [2018]. A review of the use of HS-AFM for materials science applications can be found in Payton et al. [2016].

##### 3.2.4.3 Electrochemical scanning tunnelling microscopy

Scanning tunnelling microscopy (STM) images a surface by ‘quantum tunnelling’ when a sharp probe, often made of W or Pt–Ir alloy, is deployed close to the surface (0.5–1 nm) such that the electron wavefunctions of the probe tip and specimen overlap [Flewitt and Wild, 2017]. A tunnelling current is generated by applying a bias voltage between the probe tip and the specimen [Chen, 2007; Yagati et al., 2014] as a result of electrons tunnelling through the electrically-insulating vacuum gap. Since the tunnelling current is exponentially dependent upon the distance between the probe tip and the specimen surface, it provides an accurate measure of the current and, as such, high-resolution, real-space visualisation of the surface topology (typical vertical resolution of 0.01 nm). Similarly, EC-STM images the surface of a specimen by measuring changes in the tunnelling current between the tip and the surface. However, in contrast to standard STM, EC-STM requires inclusion of an electrochemical cell and a suitable probe [Chen, 2007; Kissinger and Heineman, 1983; Yagati et al., 2014]. This technique is related to conventional AFM since both are forms of SPM, and suffer from long measurement time (relative to the corrosion processes). As such, both STM and EC-STM are designated as indirect techniques.

**Figure 20:**
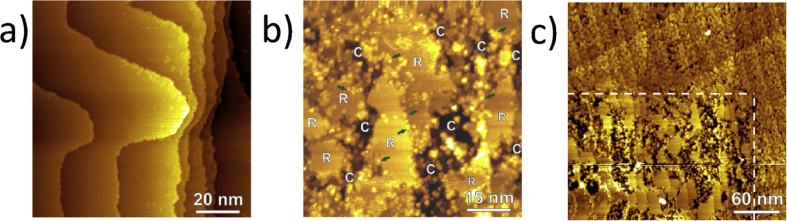
Topographic STM images of a nickel (111) surface undergoing dissolution in 0.1 mol dm^−3^ (aq): (a) -550 mV (Vs. SHE) after 180 s; (b) after 7320 s [Seyeux et al., 2008].^19^ Areas resistant to corrosion are marked “R”, and areas of localised corrosion are marked “C”. (c) shows the area continuously scanned (white dashed line) and a larger area after a period of 8320 s.

EC-STM is well suited for the monitoring of EAC initiation mechanisms, such as pitting and surface dissolution, as well as inhibition processes, like the formation of passive films and the effects of inhibitors, in a number of material/environment combinations. The mechanisms by which passive films break down, and the ways in which they self-repair, are of particular interest in the context of environmental degradation. Figure 20 shows application by Seyeux et al. [2008] to nickel with measurements highlighting alterations in structure induced by prolonged polarisation occurring in at a (111) single-crystal surface in 0.1 M aqueous sodium hydroxide during active/passive transition.

Since STM and EC-STM provide high spatial resolution (typically ~0.1 nm) for processes occurring on the very-small-scale, they can be applied to understanding mechanisms such as passive film formation or pre-initiation of pits and/or cracks.

#### 3.2.5 Surface specific techniques

**Figure 21:**
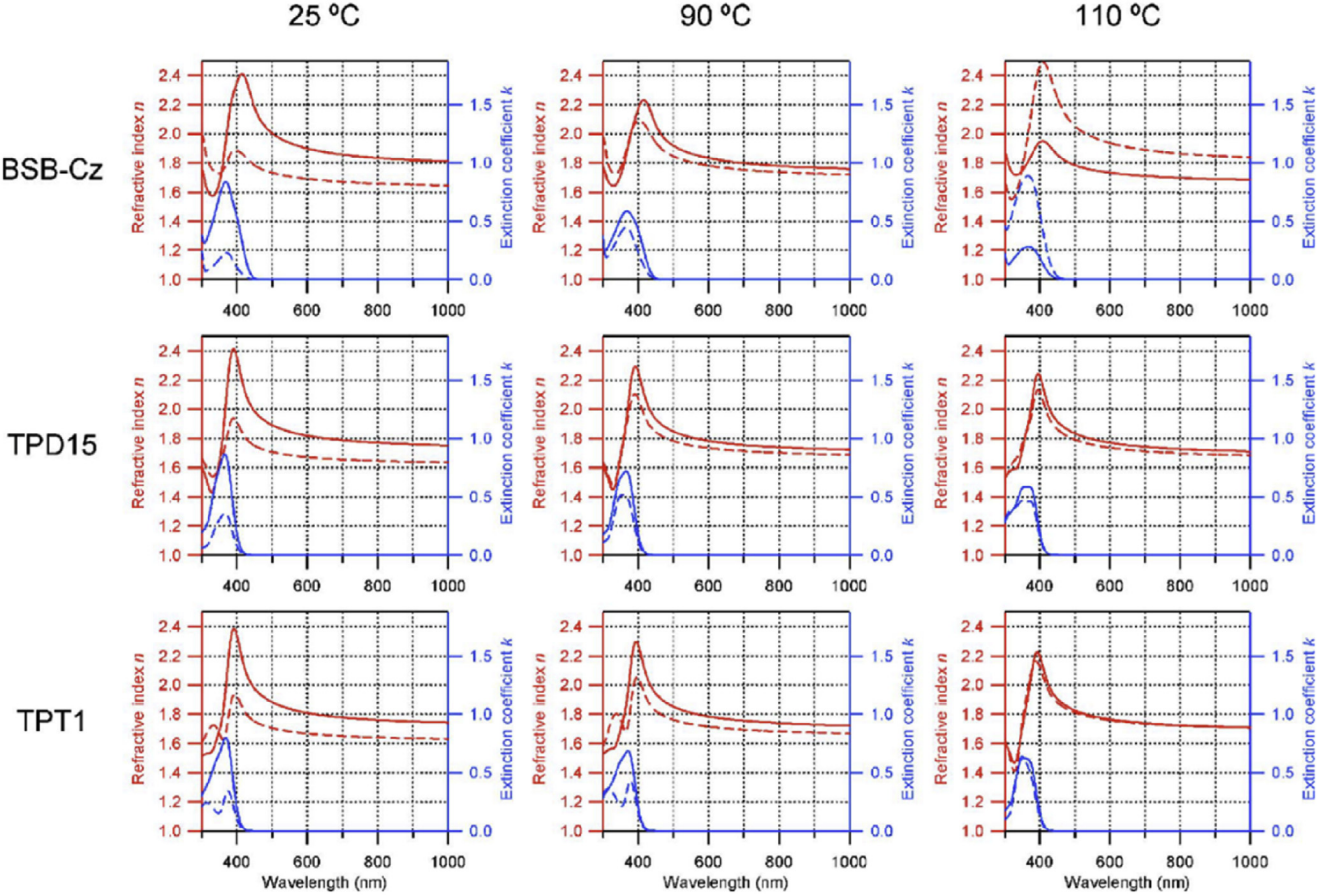
Ellipsometry on Si (100) coated with various materials: BSB-Cz (4,4′-Bis [(N-carbazole)styryl]biphenyl); TPD15 (N,N,N′,N′-tetrakis (biphenyl-4-yl) benzidine); TPT1 (N,N′-Diphenyl-N,N′-bis [4’-(N,N-diphenyl-amino)biphenyl-4-yl] benzidine) [Yokoyama and Adachi, 2010]. The plots show the variation in refractive index and extinction coefficient for different temperatures as a function of wavelength.

A wide variety of further techniques exist for probing or analysing the surfaces of metals or corrosion products. These variously allow point analysis and mapping together with imaging, which can provide information about local chemical composition and phases arising from a corrosion process. Examples of these are given below. Optical light spectroscopy measurements bring the advantage that light is generally well-transmitted through aqueous electrolytes, allowing corrosion processes to be evaluated directly *in situ*. Typically, these methods use a monochromatic laser light source [Flewitt and Wild, 2017]. Raman spectroscopy measures the shift in wavelength caused by inelastic scattering of light due to interacting with a molecule moving between two different rotational-vibrational energy states [Flewitt and Wild, 2017]. Rather than direct fitting to theoretical spectra, it is normal to use characteristic patterns for qualitative identification to allow the *in situ* identification of corrosion product phases [Li and Hihara, 2012; Maslar et al., 2001; Zhang et al., 2011]. By comparison, ellipsometry measures changes in polarisation of light reflected from a surface which can be related to the dielectric properties of very thin films on the surface. This can be used to make *in situ* measurements of oxide growth, for multi-layered passive film structures, potentially with atomic layer resolution. This method requires a near-ideal surface and is not suitable for specimens with thick or inhomogeneous surface layers. Typical length resolution is in the region of 100 μm, although measurement times can be well under a second, with nanosecond [Jellison Jr. and Lowndes, 1985] and real-time [Yokoyama and Adachi, 2010] measurements reported in the literature. Figure 21 shows how ellipsometry has been used to elucidate properties such as the refractive index, and how this changes as a function of temperature.

**Figure 22:**
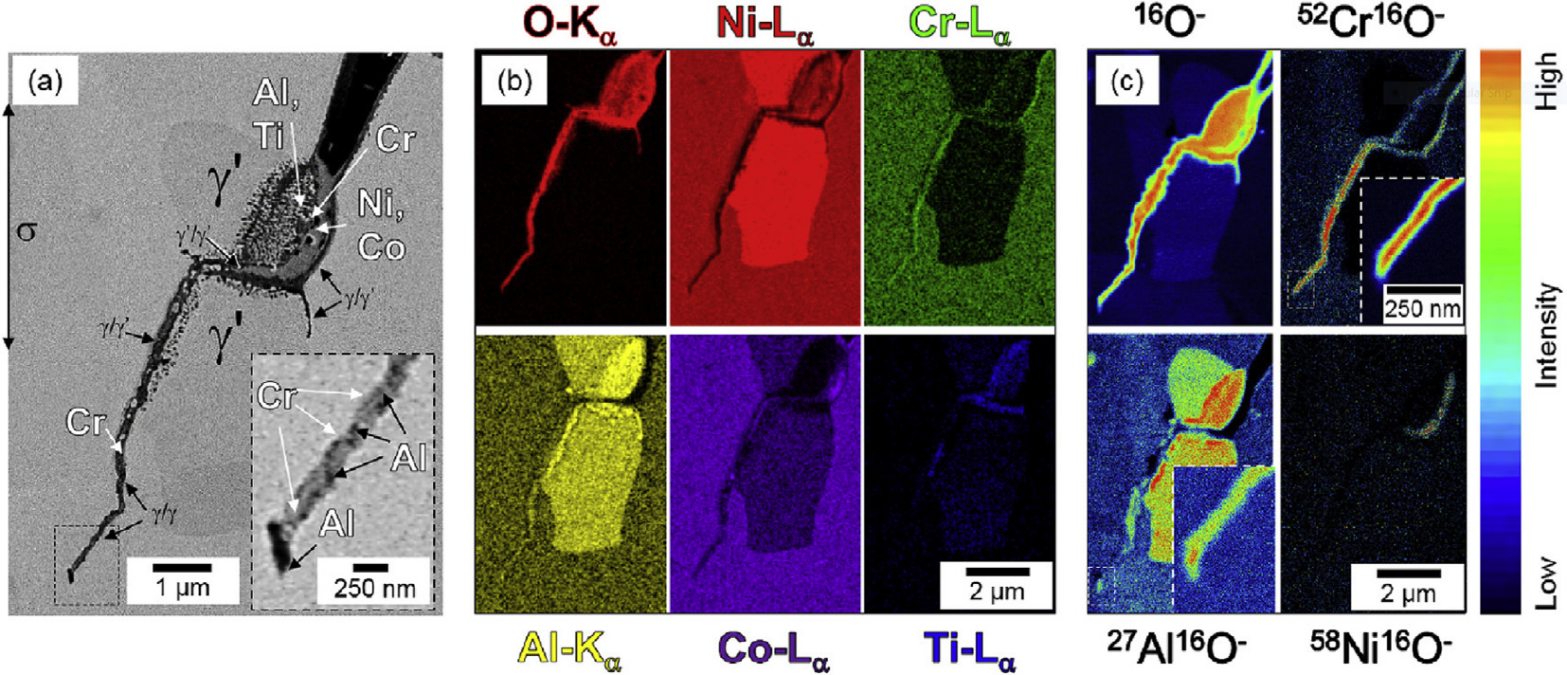
(a) Electron backscatter image of a crack tip following experiment; (b) EDS false colour mapping; (c) NanoSIMS false colour maps. The scale bar shows relative intensity for the different compositions highlighted in (c). From Nemeth et al. [2017].^20^

**Figure 23:**
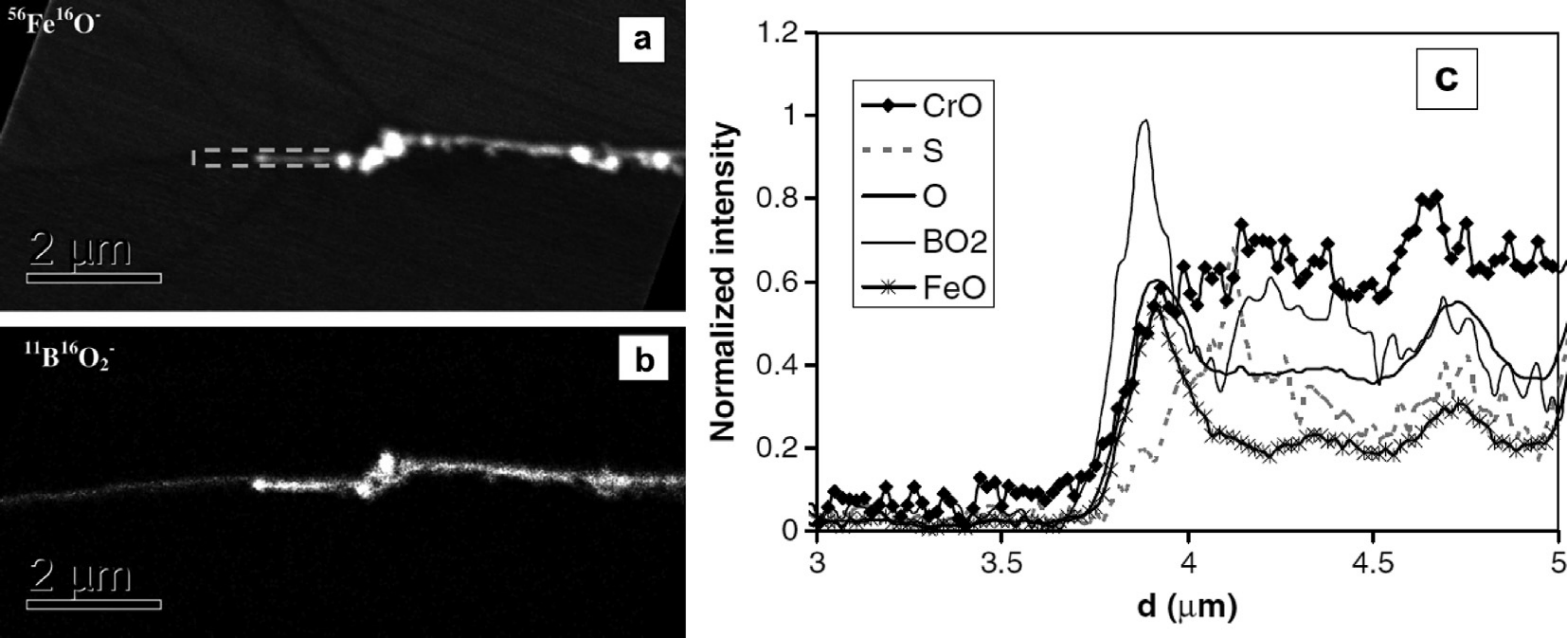
NanoSIMS analysis of an oxidised 304 stainless steel specimen following exposure to primary water environment: (a) FeO map; (b) BO_2_ map; (c) line profile along the boundary, toward the crack tip, and along the crack. From Lozano-Perez et al. [2008a].^21^

In summary, optical spectroscopy techniques can be used to examine corroding surfaces *in situ* and help evaluate EAC*.* Ellipsometry can be used to measure oxide growth on surfaces with atomic layer resolution, but its lateral resolution is limited. Raman spectroscopy allows measurement of composition and phases of materials and corrosion products. Secondary ion mass spectrometry (SIMS) is a FIB technique in which a high-energy ion beam is scanned across a specimen surface [Woodruff and Delchar, 1988]. Incident ions cause local ejection of substrate ions (and ion clusters) which are then analysed by time-of-flight mass spectrometry (ToF-MS), with isotopic resolution. This allows mapping of elemental composition with sufficient resolution to identify isotopic variations, with good coverage of light elements compared to EDS. The ability to resolve isotopes is important as this is a means of measuring the distribution of radioactive tracer species introduced into a corroding system and followed to identify active sites and species mobility [Laghoutaris et al., 2016; Woodruff and Delchar, 1988]. In addition, SIMS can be used for profiling the composition as a function of depth. NanoSIMS, a development of SIMS, allows both ppm sensitivity detection and <100 nm spatial resolution) [Lozano-Perez et al., 2014]. Nemeth et al. [2017] utilised NanoSIMS as part of an EAC investigation on the nickel-based superalloy 720Li. Since EDS was unable to differentiate the O K_α_ and Cr L_α_ spectral lines the capability of NanoSIMS provided complementary information on chemistry around an EAC crack tips (Figure 22). In addition Lozano-Perez et al. [2008a] utilised the high resolution offered by NanoSIMS to characterise SCC cracks in 304 stainless steel exposed to primary water chemistry (320 °C, 666 h). Lozano-Perez et al. [2008a and 2008b] highlighted the very high sensitivity of NanoSIMS, *i.e.* its ability to detect trace elements (S, B) around cracks, allowing detailed composition to be obtained. There is no information on the structure or crystallography. Selected area diffraction (SAD) TEM can provide crystallographic information but is both time-consuming and restricted in terms of analysis area (Lozano-Perez et al., 2008a). One example of the high resolution and ability to detect trace elements available by NanoSIMS is shown in Figure 23, where B is segregated [Lozano-Perez et al., 2008a].

X-ray photoelectron spectroscopy (XPS) measures the kinetic energy of electrons ejected from a specimen surface under X-ray irradiation [Woodruff and Delchar, 1988]. This allows point quantification of elemental composition and, in addition, electronic structure, which gives chemical information. The method is highly surface sensitive (top 1–10 nm analysed) and, hence, is a very powerful surface technique for investigating protective thin films, such as those found on the surface of stainless steels [Asami et al., 1978; Brooks et al., 1986; Clayton and Lu, 1986]. The technique uses a broad X-ray beam, which means a relatively low spatial resolution (tens of micrometres), as it is limited by the size of the aperture used, although a monochromator can increase resolution. Auger electron spectroscopy (AES) has some similarities to XPS, although the probe is an electron beam. The incident electrons cause core electron loss, which indirectly causes photon emission due to electronic rearrangement. The electron beam may be scanned across the surface by magnetic or electrostatic fields, allowing elemental composition information to be obtained [Woodruff and Delchar, 1988]. In addition, AES can be used in conjunction with SEM to attain surface specific compositional imaging through Auger electron emission and, if combined with ion sputtering, can be used to produce a profile of the composition as a function of depth. These techniques require high vacuum and so are considered indirect methods. The resolutions range down well into the nanometre-scale, although the analysis area size is normally linked to the resolution, so high-resolution limits the extent of characterisation. A degree of specimen preparation is required, although for most materials this is not highly specialised or time-consuming.

### 3.3 Volume methods

#### 3.3.1 Structural

##### 3.3.1.1 Computed X-ray tomography

**Figure 24:**
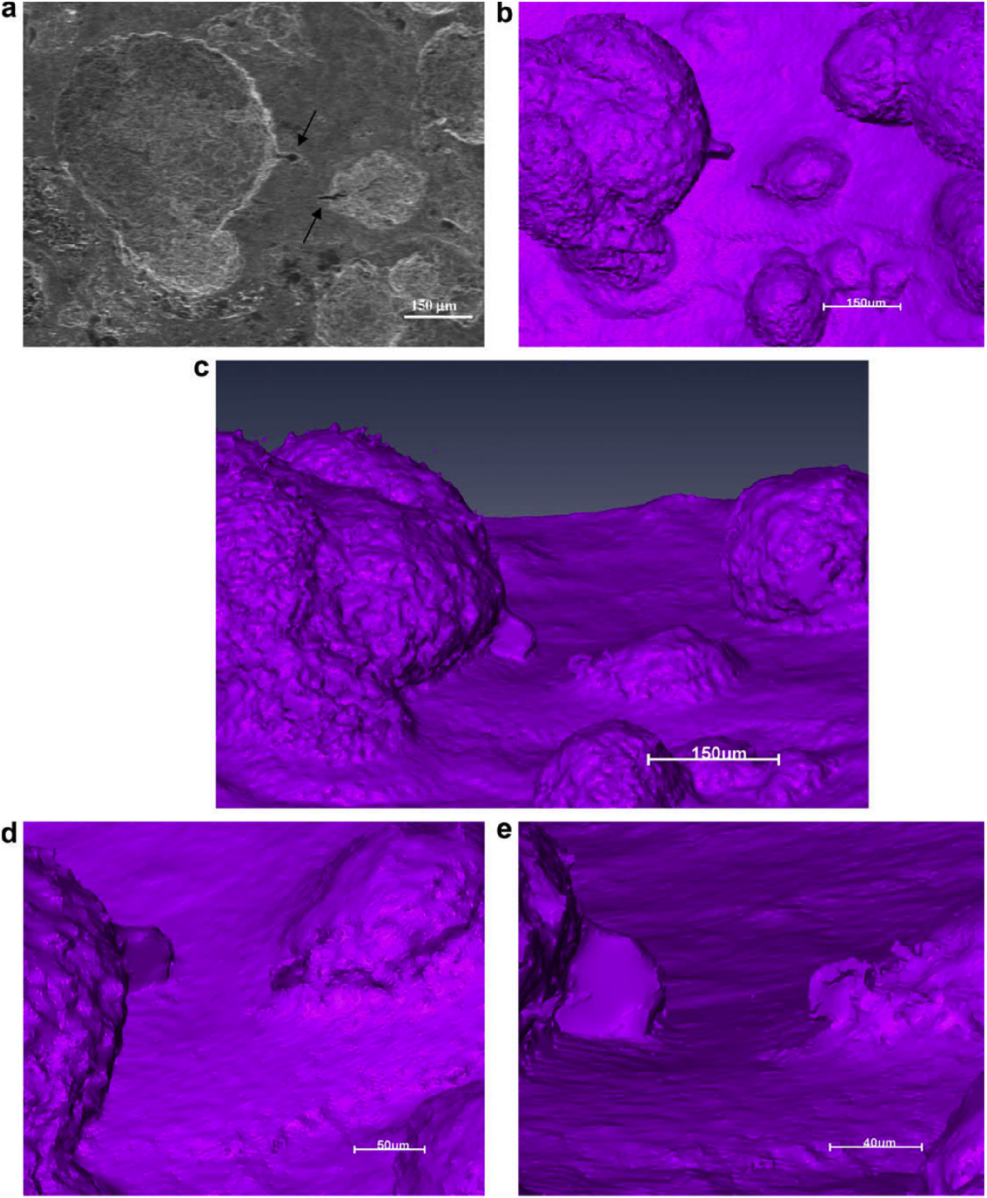
Images of a steam turbine disc following exposure to chlorinated water vapour at 90°C by: (a) SEM top down view of pit to crack transition (arrows show location of cracks): (b–e) inverted X-ray tomography of same area at various viewpoints and through from lower magnification (b, c) through to higher magnifications where detail of the cracks are revealed (d, e). From Turnbull et al. [2009].^22^

**Figure 25:**
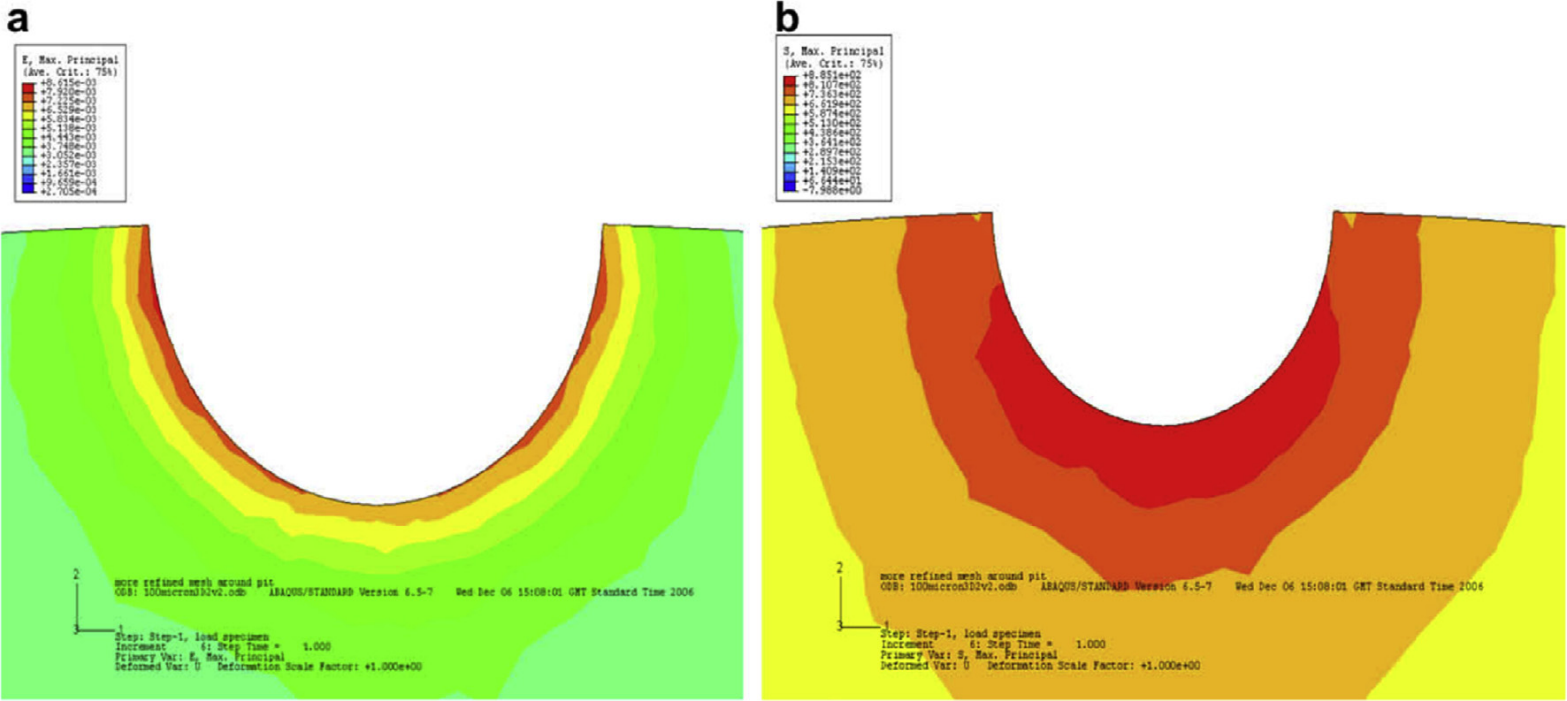
FE model showing distribution of (a) maximum principle strain; (b) maximum principle stress for a 100 μm diameter pit in plan view. Red regions denote areas of high strain and stress respectively. Green regions denote areas of low strain and stress respectively. From Turnbull et al. [2009].^23^

The majority of techniques used to characterise SCC are 2D. However, as EAC and, specifically, SCC are 3D phenomena [Lozano-Perez et al., 2011; Midgely and Dunin-Borkowski, 2009] a number of tomographic techniques have been employed. Some of these may be applied *in situ* and so are considered direct methods. High spatial resolution tomography is achieved with serial section FIB milling with gallium ions (Section 3.3.2.1). XCT allows computer reconstruction of images of internal structure based on X-ray opacity from 2D images (tomograms) observed for a sequentially incrementally-rotated specimen. Synchrotron XCT allows very high-resolution, high-speed imaging due to the intensity of X-ray radiation. Although modern laboratory sources can have comparable resolution, these typically have measurement times two orders of magnitude slower [Maire and Withers, 2014]. Analysis may be undertaken *in situ* and has allowed observation of various corrosion processes [Davenport et al., 2014; Maire and Withers, 2014; Stitt et al., 2015], including SCC initiation and propagation [Horner et al., 2011; King et al., 2008; Marrow et al., 2006; Turnbull et al., 2009]. Figure 24 shows images obtained by both SEM and XCT for a steam turbine disc exposed to water vapour containing 1.5 ppm [Cl^−^] at 90 °C. These reveal the pit-to-crack transition at the pit wall near the pit opening, complemented by FE modelling to understand the locations of maximum principle stress and strain within the pit (Figure 25).

Micro-scale precision is available, although the voxel size (resolution) that can be achieved is limited by the relative densities within the microstructure, specimen size and stability, as well as the instrument resolution. Typically, XCT spatial resolution is limited to ~1/1000th of the size of the specimen [Maire and Withers, 2014] (as of 2014). In order to increase spatial resolution, the volume of the material sampled needs to be reduced. For example, with a 1 cm^3^ specimen the voxel size would be limited to ~10 μm, whereas if the specimen was size-reduced to 1 mm^3^ a voxel size of ~1 μm may be possible.

#### 3.3.2 Composition

##### 3.3.2.1 Focused ion beam tomography

A FIB workstation, as described in Section 3.2.3, can be used to create 3D reconstructions of an area of interest within a specimen. FIB tomography involves serial sectioning of a specimen volume by iterative ion milling, imaging (either by FIB or SEM) and computed image reconstruction, allowing parallel structural imaging and compositional analyses. The latter is achieved using EDS analysis during the serial sectioning sequence so that the reconstructed volume presents an array of images and associated compositional maps. The ion beam provides images that are used for observable contrast. The structural and textural information can be high-resolution, although the precision of EDS characterisation will be constrained by the much larger interaction volume (1–5 μm^3^). Tomography provides an insight into pit-to-crack morphology and the ability to locate early stages of crack initiation, such as by Man et al. [2012], on 316L stainless steel fatigue specimens (cracked in air), and by Lozano-Perez et al. [2011], on 304 stainless steel and inconel 600 alloy exposed to PWR primary water conditions (Figures 15 and 16).

##### 3.3.2.2 Electron tomography

Electron tomography is capable of 3D visualisation of structures within thin foil specimens with nanometre resolution, as well as chemical composition [Lozano-Perez et al., 2011; Weyland and Midgley, 2003; Midgely and Dunin-Borkowski, 2009]. This technique involves the use of a FIB or dualbeam system to mill and extract TEM-sized specimens [Andrzejczuk et al., 2017]. A thin-section specimen is analysed with conventional imaging modes at a range of inclinations and the transmission data reconstructed. This method can be utilised in the analysis of SCC cracks that contain corrosion products to develop understanding of how cracks propagate within susceptible materials, as shown in Figure 27(d-f) [Lozano-Perez et al., 2011].

**Figure 26:**
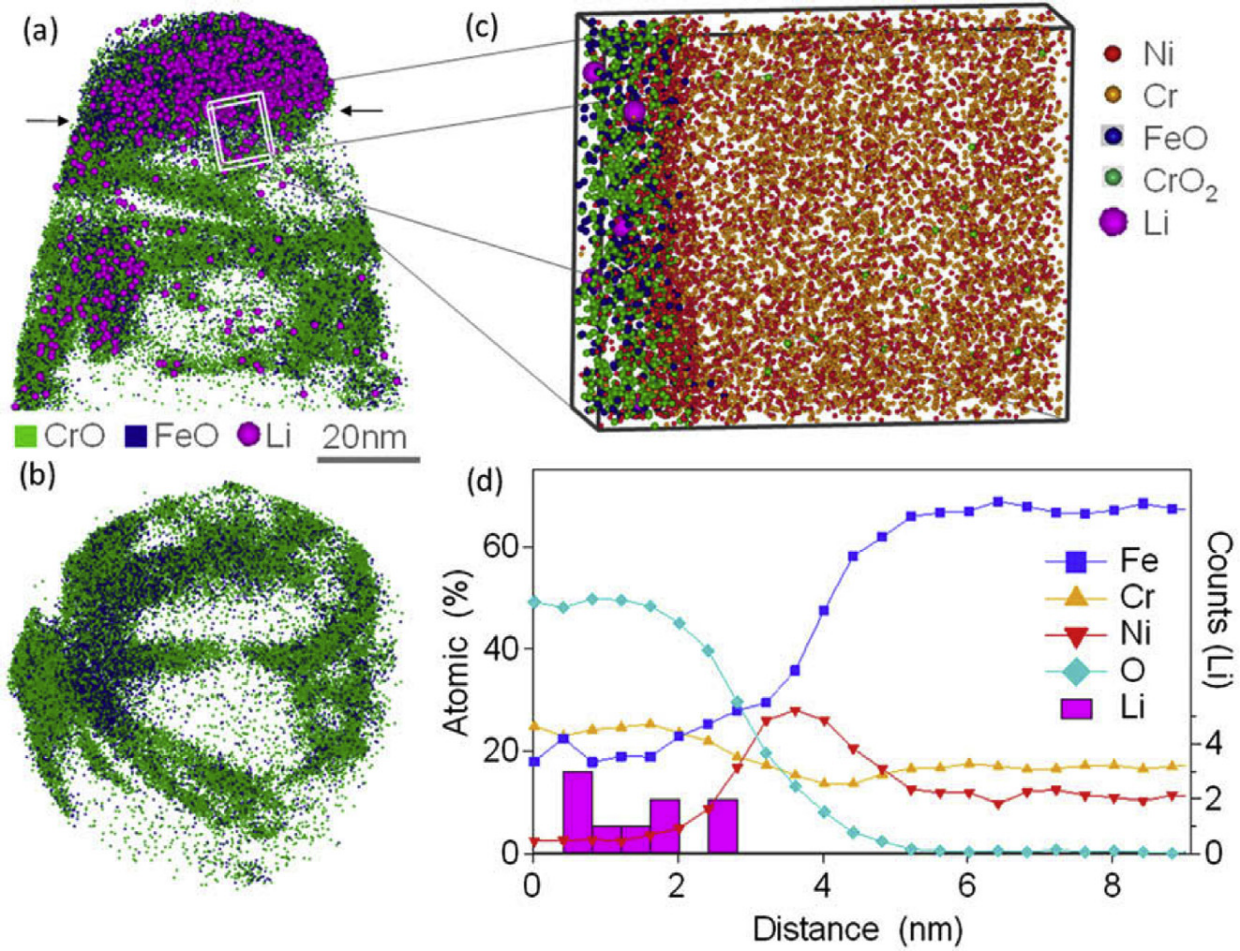
(a) APT reconstruction of oxidised 316 stainless steel needle specimen, exposed to PWR environment; (b) top-down view of the sub-interface once cap was removed; (c) small sub-volume showing the presence of selected elements; (d) line profile across the oxide-metal interface. Li is given as atom count (right axis) due to the low concentration at the interface. From Lozano-Perez et al. [2010].^25^

**Figure 27:**
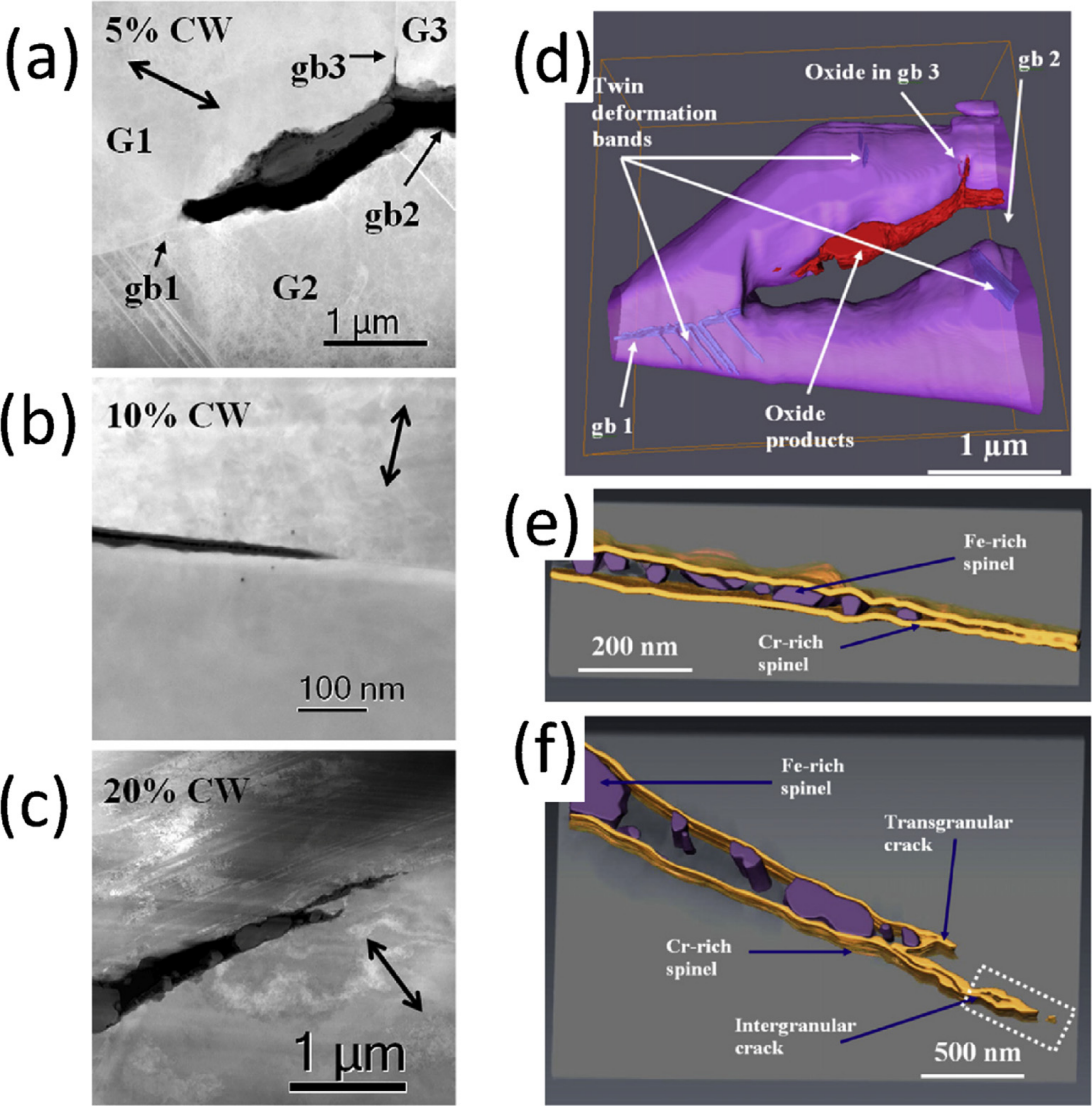
(a–c) Scanning TEM (STEM) images of SCC crack tips in 304 stainless steel exposed to PWR chemistry conditions at 320 °C with varying cold work: (a) 5%; (b) 10%; (c) 20%. (d–f) Corresponding reconstructed electron tomography models: (d) 5%; (e) 10%; (f) 20%. From Lozano-Perez et al. [2011].^24^

##### 3.3.2.3 Atom probe tomography

APT requires the preparation of extremely fine needle specimens; through electropolishing, or for site specific selection (e.g. grain boundaries, precipitates, or interfaces) milled using FIB. Key parameters for the needles are that they need to have a low shank angle and have end radii of ~50 nm. Typical needle dimensions used for analysis are ~80 × 80 × 200 nm [Meisnar et al., 2015b]. Needles are ionised to allow atomic resolution ToF-MS elemental analysis, followed by volume reconstruction. An example of the application of this technique has involved exposure of prepared SUS304 stainless steel specimens to an EAC environment (320 °C PWR primary water; 666 h), allowing characterisation of extremely small-scale reaction volumes [Cerezo et al., 2007]. APT provides very high spatial resolution for chemical analysis [Cerezo et al., 2007]. Example APT maps are shown in Figure 26 for 20% cold worked 316 stainless steel exposed to a PWR environment for a period of 1500 h [Lozano-Perez et al., 2010].

Lozano-Perez et al. simplified the maps to show the oxides of Fe and Cr, with Li atoms superimposed. The figure reveals the inner surface oxide rich in Cr (shown in green) as the Fe-rich oxide (shown in blue) is only weakly adherent to the surface, and prone to fracture during the APT analysis. The sub-volume map and cross-section (Figures 26c and 26d) show the incorporation of Li, present in PWR primary water, within the Cr oxide spinel. These workers note that the presence of B, another important additive in PWR primary water, could not be resolved in this experiment. The authors did, however, utilise NanoSIMS characterisation on the same oxide specimen [Lozano-Perez et al., 2008b], which indicated that B may be enriched only in the outer part of the outer Cr-rich spinel layer, intentionally milled away for APT during the FIB sample preparation procedure. APT allows site specific analyses; such as locations susceptible to EAC (e.g. regions ahead to the crack tip), which assists in characterisation of the EAC mechanism. The main constraints are the very small analysis volume (typically ~100 × 50 × 50 nm) and complex specimen preparation. Accordingly, the main practical uncertainty with this technique is how representative the sampling is of the bulk material.

FIB, electron, and APT provide *ex situ,* indirect measurement which is akin to XCT, as outlined earlier. Each of these techniques allows the investigation of precipitate, solute and impurity distribution within the material. FIB tomography can identify and characterise crack tips with high spatial resolution compared to XCT voxel sizes. Thus, a combination of complementary techniques can be used, such as described by Lozano-Perez et al. [2011], where FIB tomography, electron tomography and high-angle annular dark field TEM were used to examine crack tips in 304 stainless steel exposed to PWR chemistry and conditions (Figure 27).

Burnett et al. [2014] combined techniques to study intergranular corrosion on sensitised 316H stainless steel as part of a correlative workflow. These included the different length-scale techniques of *in situ* XCT, *ex situ* higher resolution XCT, FIB tomography, EBSD and scanning TEM (STEM) EDS. Electron tomography is even more spatially-resolved than FIB tomography, with resolutions of <1 nm. Resolution is dependent on the number of individual tomograms collected as these ultimately define the voxel size. APT can give detail on the elemental composition of materials with the highest spatial resolution. Each of these composition-based techniques has a higher spatial resolution than XCT but requires destruction of the specimen.

### 3.4 Reaction sensing

#### 3.4.1 Electrochemical techniques

In general, measured macroscopic electrochemical behaviour is an average of heterogeneous reactivity over an electrode surface. As such, it can include contributions from: (i) different active local sites; (ii) crystal orientation; and (iii) surface defects. There is a great diversity of methods employing electrochemical methods [Du et al., 2011], and these have a general advantage that they are direct, making possible *in situ* measurements. However, there is a need when addressing EAC to map surface reactivity at length-scales down to the nano-scale. To accommodate this high spatial resolution challenge for local electrochemical information, probes with dimensions smaller than the required spatial resolution have to be adopted, together with stable and precise control of the position [Zoski, 2016].

Corrosion processes can be monitored relatively simply by employing either open circuit or potentiodynamic experiments to provide insight into the electrochemical behaviour of the system [Rossi et al., 2008]. To probe more complex behaviours, alternating current techniques (electrochemical impedance spectroscopy (EIS); electrochemical noise (EN)) can be employed, and have been used to detect and interpret the processes occurring during corrosion (Section 3.4.1.3). Unfortunately, these methods are severely limited by their spatial resolution, although this may be addressed with consideration and/or control of geometry, notably of the crack tip [Crane et al., 2016; Turnbull et al., 2004]. As a result, considerable effort and research over the past few decades have produced improved techniques with increased spatial resolution [Rossi et al., 2008]. These include localised EIS and SVET [Asmussen et al., 2015; Oltra et al., 2007; Rossi et al., 2008; Zhang and Cheng, 2009]. A further approach is to confine the area under study, by using micro-electrodes, possessing very small surface areas [Bonzom and Oltra, 2016; Cook et al., 2012a; Laycock and Newman, 1997; Speckert and Burstein, 2011; Tang and Davenport, 2007], or droplet cells, in which a very small proportion of a larger surface is exposed to solution (Section 3.4.1.2).

##### 3.4.1.1 Scanning vibrating electrode technique

**Figure 28:**
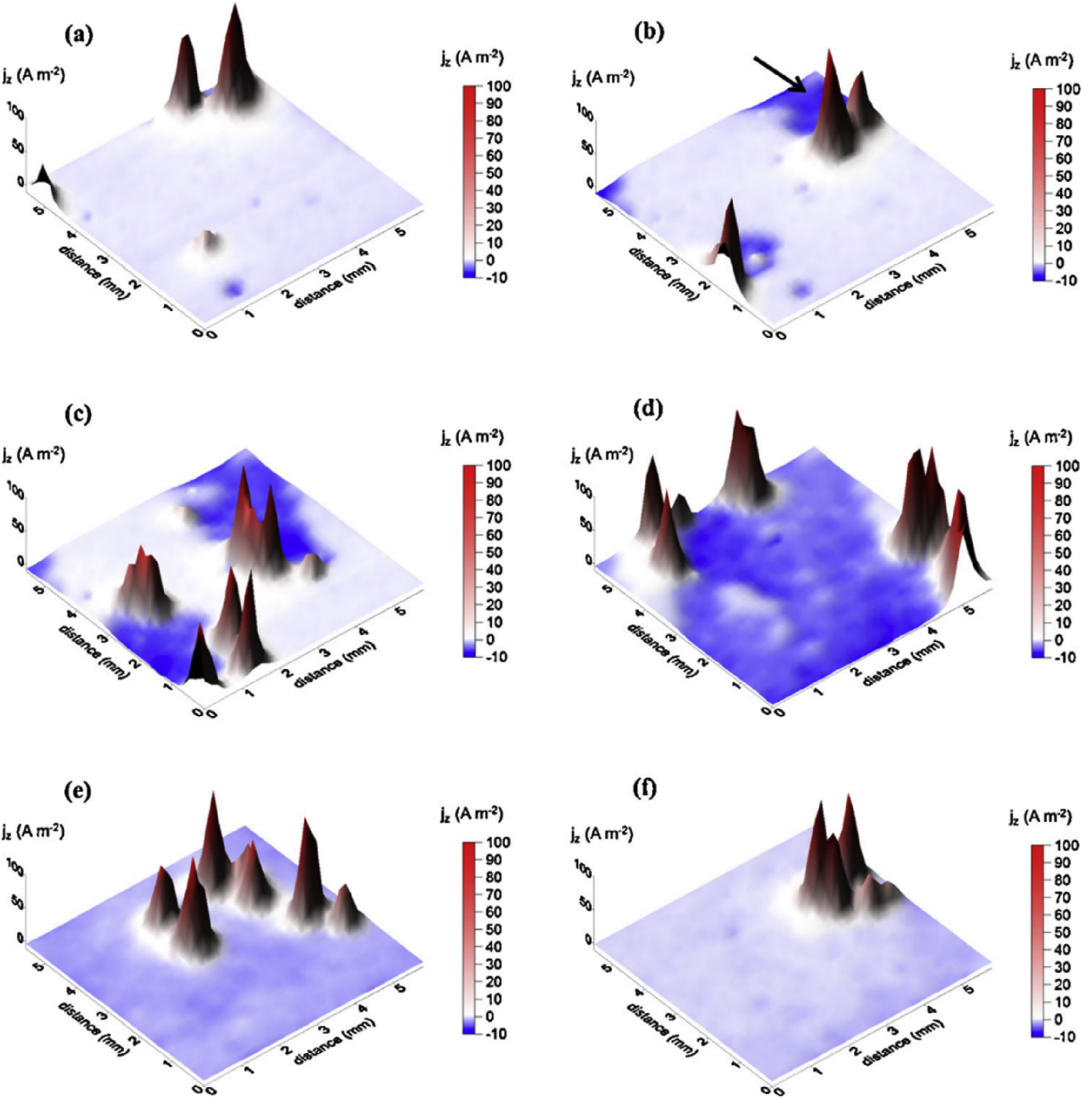
Examples of SVET surface current density maps (*j*_*z*_) obtained from AZ31 magnesium alloy exposed to 5% NaCl w/v (aq) following (a) 12 min; (b) 30 min; (c) 60 min; (d) 2h; (e) 4h; (f) 12h exposure. The scale bar shows current density in units of Am^−2^. From Williams et al. [2013].^26^

**Figure 29:**
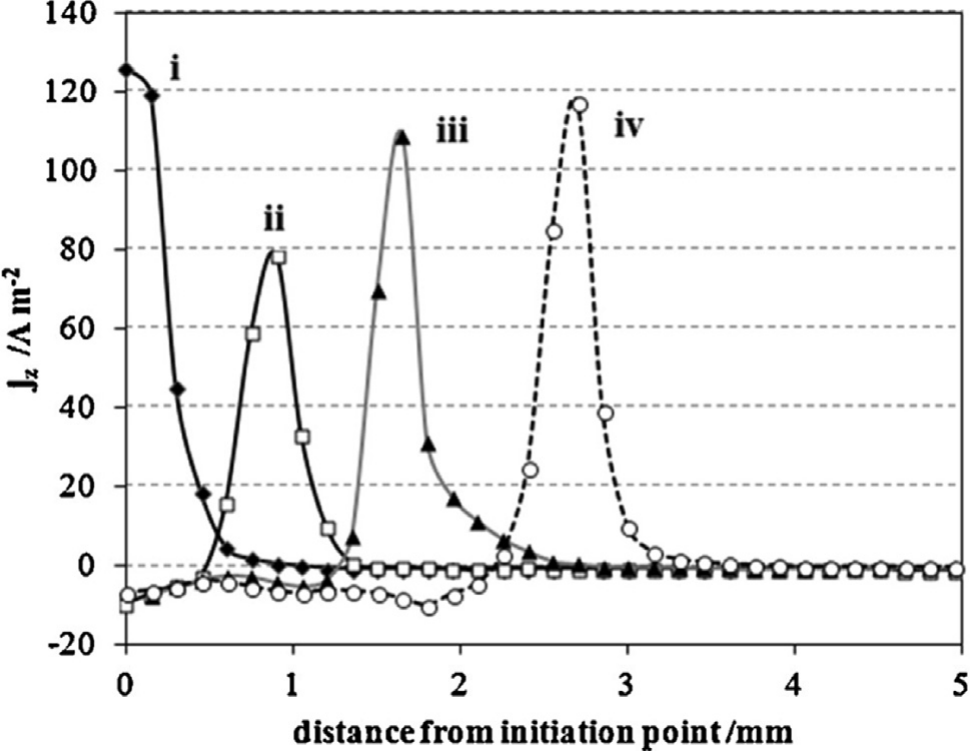
Plots of surface current density (*jz*) as a function of distance showing the rate of corrosion propagation with time: (i) 12 min; (ii) 24 min; (iii) 32 min; (iv) 50 min. The location which was used as the initiation point is indicated by the black arrows in Figure 26(b). From Williams et al. [2013].^27^

SVET is an electrochemical technique that can be implemented to measure corrosion initiation [Laferrere et al., 2017; Pyun and Lee, 2012; Williams and McMurray, 2006; Williams and McMurray, 2008; Williams et al., 2013; Williams et al., 2015; Worsley et al., 1997] and subsequent propagation *in situ* as a direct technique. Figure 28 shows how SVET can be used to identify localised corrosion initiation sites, and the distribution of anodic (red in Figure 28) and cathodic (blue in Figure 28) current density (*j*_*z*_) across the surface of a magnesium alloy immersed in aqueous NaCl. Surface current density maps can be obtained, together with line scans to reveal the rates of corrosion propagation across a corroding surface, as shown in Figure 29. This method provides an insight into kinetics and mechanisms of corrosion within aqueous environments [Mehrazi et al., 2016; Worsley et al., 1997].

**Figure 30:**
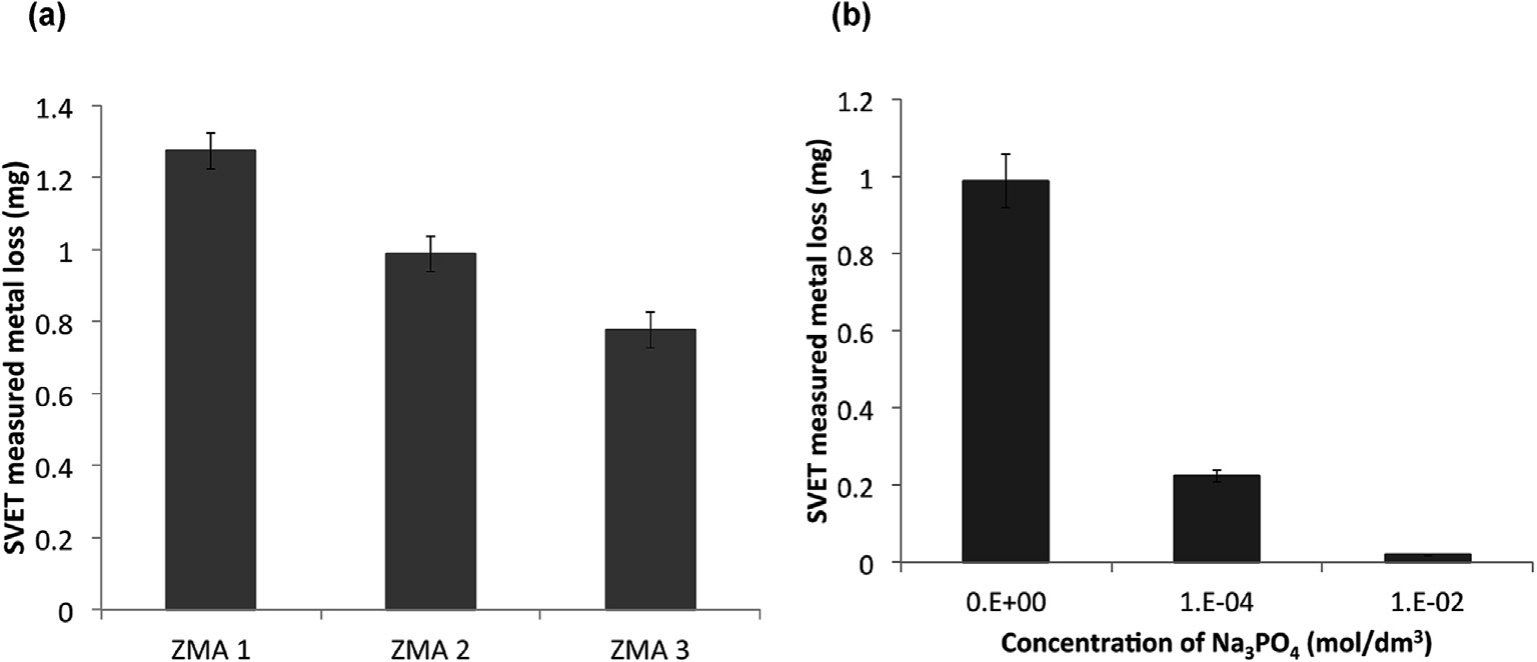
(a) SVET measured mass loss for different zinc-magnesium-aluminium alloys over a period of 24 h following immersion in 1% NaCl; (b) SVET measured mass loss for ZMA 2 with the use of varying concentrations of corrosion inhibitor. From Sullivan et al. [2015].^28^

Local potential gradients within a solution are measured by a vibrating micro-electrode scanned at a nominal height above the surface of a corroding specimen [Cheng and Sharma, 2011; Fayyad et al., 2014 Rossi et al., 2008; Worsley et al., 1997]. These potential gradients are proportional to the current density. Once the micro-electrode has performed a raster scan across the surface, a map of current density can be obtained. These maps, repeated over specific time intervals, allow the monitoring of anodic and cathodic regions with ~100 μm spatial resolution. Although activity is still measured for smaller couples, the sensitivity is reduced. Measurement times are typically 20 min for one scan of 10 mm^2^ area. SVET has been used for investigations into corrosion mechanisms and control [Fayyad et al., 2014; Geary et al., 2013; Laferrere et al., 2017; Rossi et al., 2008; Williams et al., 2010; Williams et al., 2015]. Subject to a set of assumptions, a simple calculation using Faraday's law can then allow corrosion rates to be estimated (Figure 30) [Sullivan et al., 2015]. There are opportunities for the technique to be used in co-ordination with stress applied to a test specimen, such as adopted for three-point bend or slow strain rate tests. In such an experiment, at the site where pit-to-crack transition is present anodic activity would be detected by SVET. The values of current density obtained through this method could then be input into predictive SCC models to both inform and verify the modelling.

##### 3.4.1.2 Scanning Kelvin probe

SKP is a high-sensitivity, electrochemical scanning technique which is capable of measuring work functions of metals and alloys. SKP is an indirect technique and does not require the electrode (Au wire probe) to be immersed and, therefore, is suitable for monitoring of atmospheric corrosion. This is possible as, once calibrated, the technique is able to measure the electrode potential of surfaces even when covered by very thin electrolyte layers, which may be resistive, or thin organic and inorganic coatings [McMurray and Williams, 2010]. Since the SKP technique does not measure current, the rate of reactions cannot be determined. As with SKPFM (Section 3.2.4.1), SKP measures the Volta potential difference, or contact potential, between the two metals (probe and specimen) when separated and when an external connection is made. This arises because when the metals are separated Fermi levels are independent of one another. However, when connected the Fermi levels equalise, leading to the Volta potential difference. The probe is then set to oscillate normal to the sample surface, thus establishing an alternating current between the two metals. As the two metals are separated by a dielectric medium, they act as two plates of a parallel plate capacitor storing an electrical charge. An external bias potential is then applied to null the current, which is inversely proportional to the change in work function between the reference probe and the sample. Both the probe-to-sample distance and the probe diameter are important variables which constrain the spatial resolution of the SKP technique [McMurray and Williams, 2002]. For a 125 μm tip diameter and 100 μm probe-to-specimen distance, the lateral resolution is ~140 μm; with the lateral resolution controlled by whichever of these two parameters is largest.

**Figure 31:**
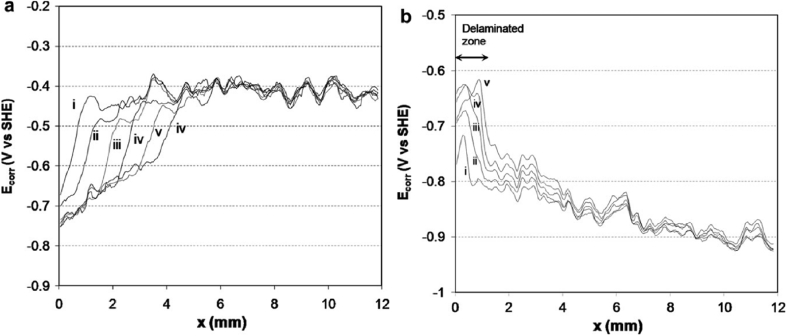
Profiles of E_corr_ with distance as a function of time for a hot-dip-galvanised zinc substrate with a polyvinyl butyral (PVB) coating containing: (a) 0.05; (b) 0.3 Zn^2+^ cross-linked sulphonated polystyrene pigment volume fractions. The curves show the rate of coating delamination away from a coating defect. From Williams et al. [2012].^29^

**Figure 32:**
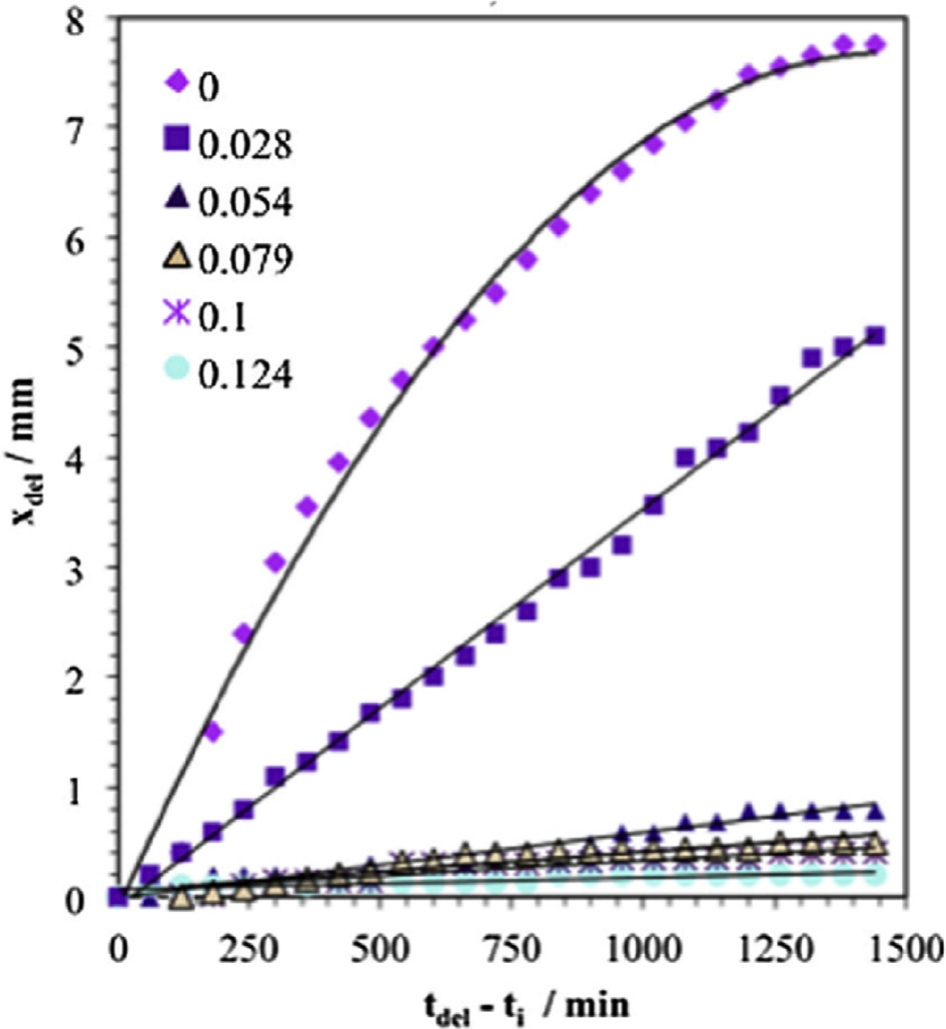
Plots of delamination distance with time for PVB coatings containing different pigment volume fractions of GNP (shown in legend). From Glover et al. [2017].^30^

The technique has been used to study atmospheric corrosion, including filiform corrosion [Williams and McMurray, 2003] and under-film corrosion through cathodic disbondment processes [Glover et al., 2017; Williams and Grace, 2011; Williams et al., 2012]. Figure 31 shows how SKP can be used to study the change in free corrosion potential (E_corr_) away from a coating defect on a coated hot-dip-galvanised zinc substrate. Figure 32 shows how this information can be used to calculate the time required for coating delamination to occur for a PVB coating combined with graphene nanoplatelets (GNP) on an iron substrate [Glover et al. 2017].

At present this technique is suited for investigating different corrosion mechanisms, such as those listed above. However, the SKPFM technique can complement macro-scale SKP by offering more amicable nano-scale resolution.

##### 3.4.1.3 Electrochemical micro-capillary technique

Silicon-coated glass capillaries may be employed as individual electrochemical cells. By positioning a capillary at specific points on a specimen surface, features such as grain boundaries can be individually targeted using an optical microscope and analysed at the micro-scale, without exposing the entire surface to the electrolyte. This technique allows localisation of test methods at the micrometre range (smallest useful tip diameter is 1–5 μm [Suter and Bohni, 2001]), with a time resolution that is dictated by the type of testing undertaken, but which can be in the micro-second range. The micro-capillary can be formed by pulling a heated glass tube to give a conical shape at one end. The end of the tube is then dipped into a silicone lacquer; the inner is then removed using a solvent. The quality of the silicone and procedure determine the quality of the experiment as they affect the possibility of crevice corrosion under the tip [Suter and Bohni, 2001]; silicone is hydrophobic and so electrolyte permeation under the tip is discouraged. Furthermore, the elastic properties of the silicone allow it to conform to curved or rough surfaces, reducing the possibility of crevice corrosion. The quality of the experiment is determined by tip diameter, limited by two factors: (i) potentiostat input resistance, which is required to be two orders of magnitude greater than the specimen resistance; and (ii) potentiostat current sensitivity, to be a minimum of one order of magnitude lower than the passive current [Suter and Bohni, 2001].

**Figure 33:**
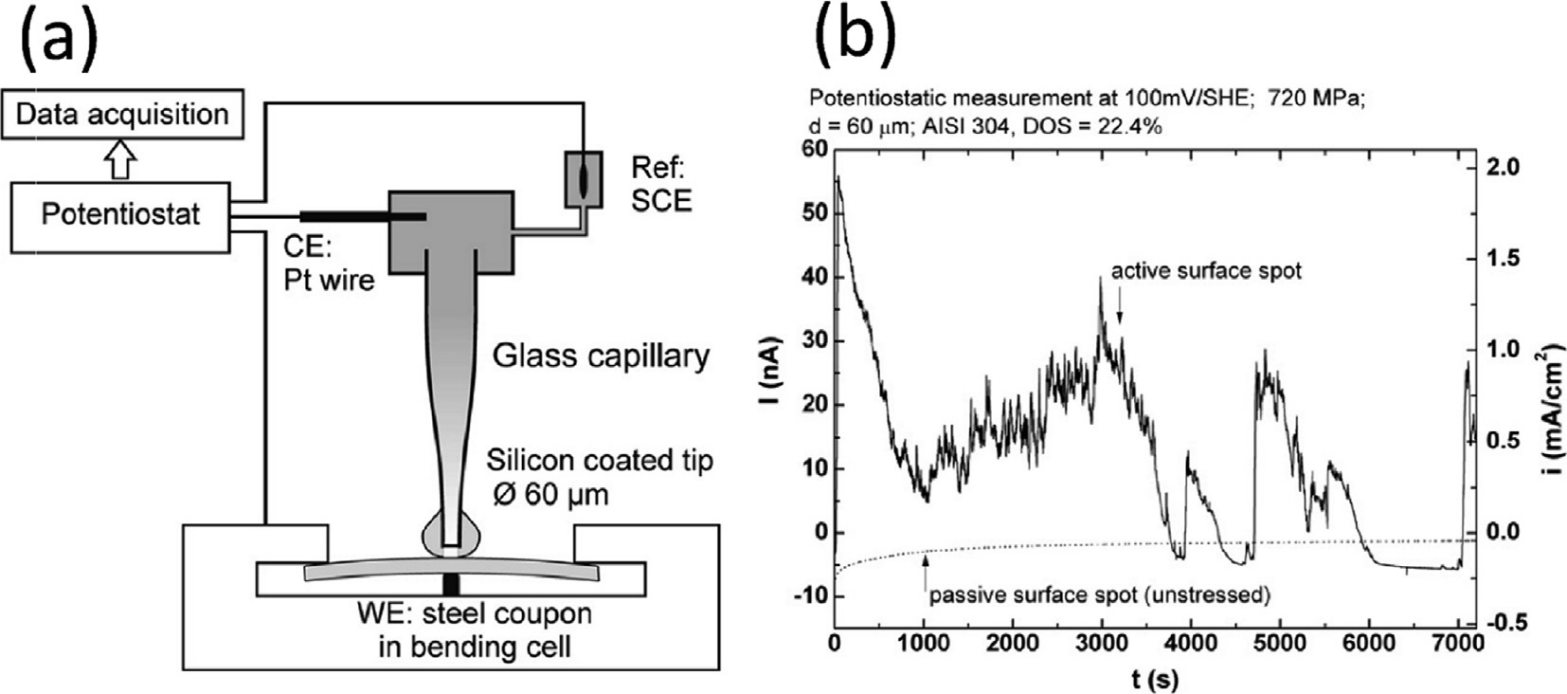
(a) Schematic of the electrochemical micro-capillary technique used by Breimesser et al. [2012]^31^; (b) results from potentiostatic experiment using the micro-capillary technique with a stressed sensitised 304 stainless steel specimen.

Breimesser et al. [2012] applied electrochemical micro-capillaries to measure characteristic potential signals produced by crack initiation and propagation due to IGSCC in stainless steel (Figure 33(a)). These showed a series of stepwise peaks (Figure 33(b)), representing sharp current increases (rapid metal dissolution) and subsequent slow exponential current decay (repassivation). These results indicated that IGSCC is discontinuous and best considered as a series of active and passive steps, as described by the film rupture model.

##### 3.4.1.4 Electrochemical noise (EN)

Since SCC growth processes can occur relatively rapidly, efforts have been directed at transient techniques to determine the mechanisms involved [Kovac et al., 2010]. The measurement of EN is a non-intrusive technique that is able to detect individual electrochemical events during SCC in real-time for *in situ* conditions [Calabrese et al., 2015a, Calabrese et al., 2015b; Du et al., 2011; Klapper et al., 2010; Kovac et al., 2010; Pyun and Lee, 2012]. As a result, this technique is particularly suited for the detection and following of the early stages of localised corrosion [Klapper et al., 2010]. It involves the monitoring of electrode potential and current fluctuations that occur spontaneously as a result of corrosion reactions [Calabrese et al., 2015a, Calabrese et al., 2015b; Du et al., 2011; Fayyad et al., 2014; Kovac et al., 2010; Pyun and Lee, 2012].

**Figure 34:**
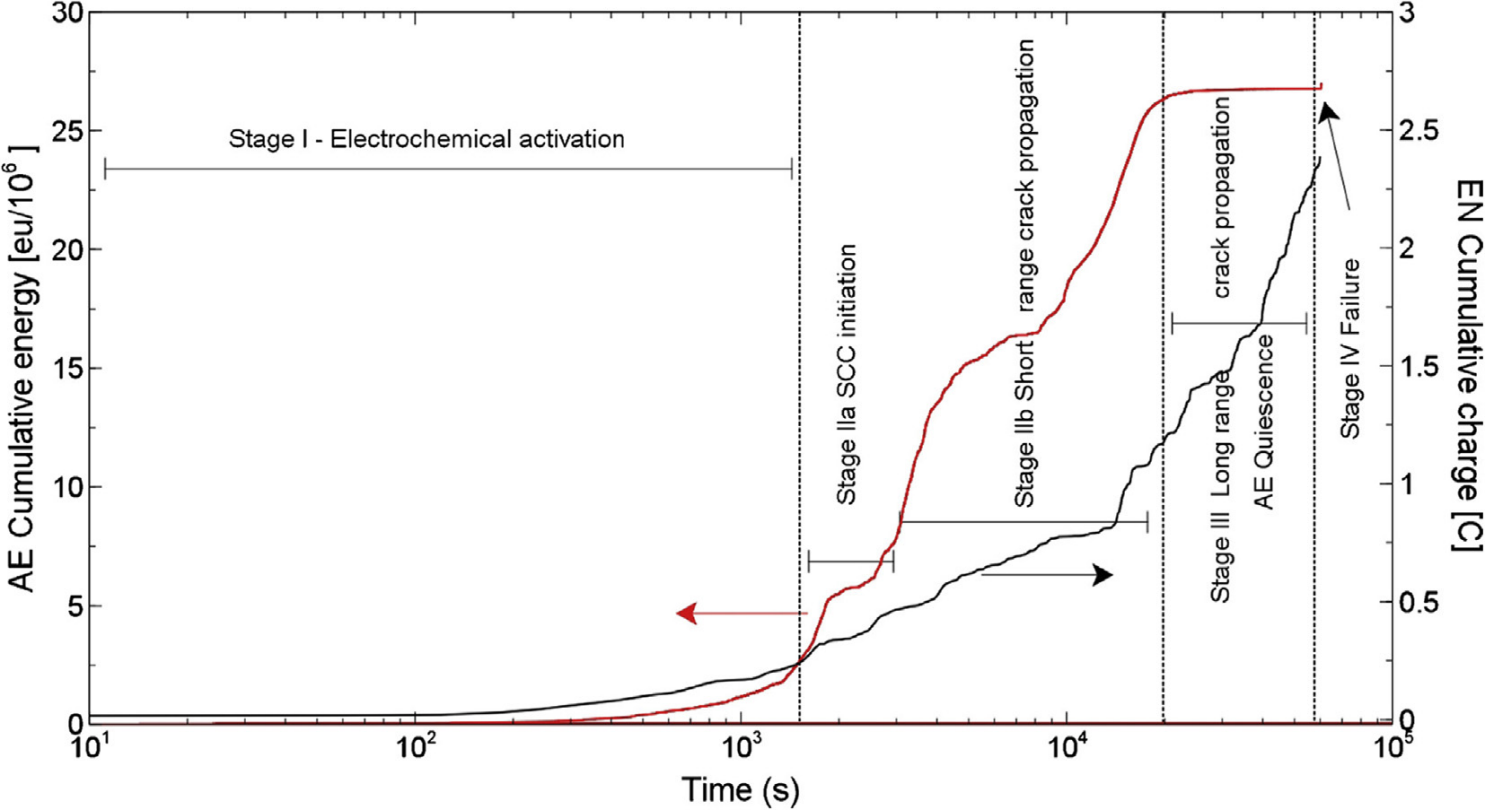
Cumulative acoustic emission (AE; red) and electrochemical noise (EN; black) as functions of time during SCC of 17-4 PH stainless steel, showing the four distinct damage stages. From Calabrese et al., 2015b, Calabrese et al., 2015a.^32^

EN measurements are not normally conducive to identifying the locality of reactions, although they can be used effectively with micro-electrode approaches to achieve this with micrometre accuracy. However, it would be challenging to deploy on a stressed test specimen. Data can be collected in the millisecond regime. There have been various interpretations of EN signals [Breimesser et al., 2012]. Calabrese et al. measured EN signals of SCC events for 17-4 PH stainless steel in a solution of hot magnesium chloride (Figure 34) [Calabrese et al., 2015a, Calabrese et al., 2015b]. Using these data, they were able to label the damage phases: stage I, electrochemical activation; stage II, propagation; stage III, acoustic emission (AE) quiescence; and stage IV, failure [Calabrese et al., 2015a, Calabrese et al., 2015b; Kovac et al., 2010]. The electrochemical activation stage involves localised thinning of the passive film, until depassivation has occurred. At this stage, metastable pit nucleation takes place in these locations. In stage II, successful metastable pits grow and stable pitting occurs. This is the time in which pit-to-crack initiation can occur, defined as short-range crack propagation, and the point where there is competition between pitting and SCC propagation. Stage III can be defined by long-range crack propagation, where electrochemical dissolution of the crack tip takes place. Once the crack tip reaches a critical geometry and is subject to the required stress intensity factor the final stage (IV) is reached, where the crack propagates instantaneously and the specimen fails. In contrast to some other methods, EN is capable of distinguishing between different mechanisms for corrosion: crevice corrosion; stable and metastable pitting; uniform corrosion; and SCC [Kovac et al., 2010; Du et al., 2011; Fayyad et al., 2014; Calabrese et al., 2015a, Calabrese et al., 2015b]. Studies into the corrosion of austenitic stainless steels in various environments have demonstrated that by monitoring EN fluctuations it is possible to detect the initiation and periodic propagation of cracks in SCC tests [Calabrese et al., 2015a, Calabrese et al., 2015b; Kovac et al., 2010]. Research has already been performed using EN measurements to study SCC mechanisms, and this ability could allow insight into the evolution of corrosion over time. Loto and Cottis [1987] used EN to study SCC of α-brass and SCC on 7000 series aluminium alloy [Loto and Cottis, 1989].

**Figure 35:**
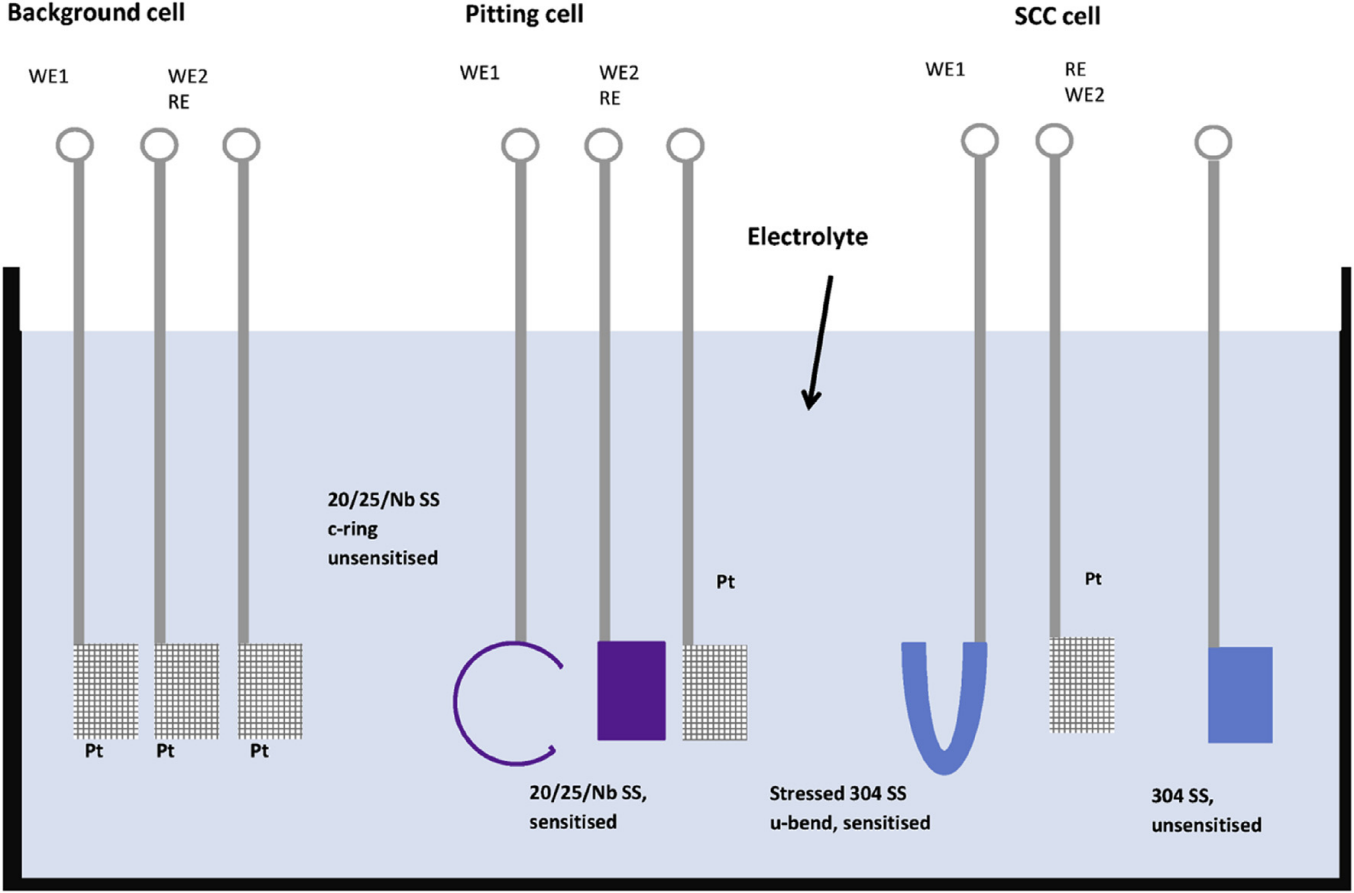
Schematic showing the multi EN cell (background, pitting and SCC cell) experimental set up used by Clark et al. [2018]^33^ to differentiate between different types of corrosion processes using the EN technique.

Clark et al. [2018] demonstrated a novel *in situ* approach to monitoring corrosion processes using EN on stainless steels, using three separate EN cells with asymmetric electrodes. EN measurement was made simultaneously with the three cells located in the same electrolyte. The first cell consisted of three Pt electrodes, and filtered background noise as no corrosion was expected to occur. The second consisted of two stainless steel working electrodes and a Pt pseudo reference electrode to allow monitoring of background noise, general corrosion and pitting corrosion (Figure 35). Finally, a third cell using a stressed, sensitised stainless steel electrode and a second, unstressed, unsensitised stainless steel electrode, alongside a Pt pseudo reference electrode, monitored general corrosion, pitting corrosion and SCC. By considering the signals obtained from each of the individual cells, the authors were able to identify SCC EN signals from background noise, pitting corrosion noise and general corrosion noise with changes in chemistry. Clark et al. [2018] showed that EN was able to monitor changes in potential and current at relatively low chloride concentrations (1–20 ppm chloride).

The experimental set-up used by Clark et al. [2018] was specially used for the experiments on 20/25/Nb stainless steel, which can be affected by IGSCC in spent fuel storage due to a combination of neutron flux and prolonged use at temperature when in service [Clark et al., 2020]. The material can be thermally sensitised to induce IGC. The current understanding however is that the material does not exhibit IGSCC in a heat-treated state, therefore sensitised 304 stainless steel was used as an analogus material during the investigation. It is noteworthy that the authors recommend development of the multi-cell technique for future industrial applications. In order to fully understand the data generated (different stainless steels were used in the experiment) the same composition stainless steel, in the same condition, may be utilised for the working electrodes (e.g. thermally sensitised, or unsensitised), which would then give a clearer indication of the processes occurring.

##### 3.4.1.5 Scanning electrochemical microscopy

**Figure 36:**
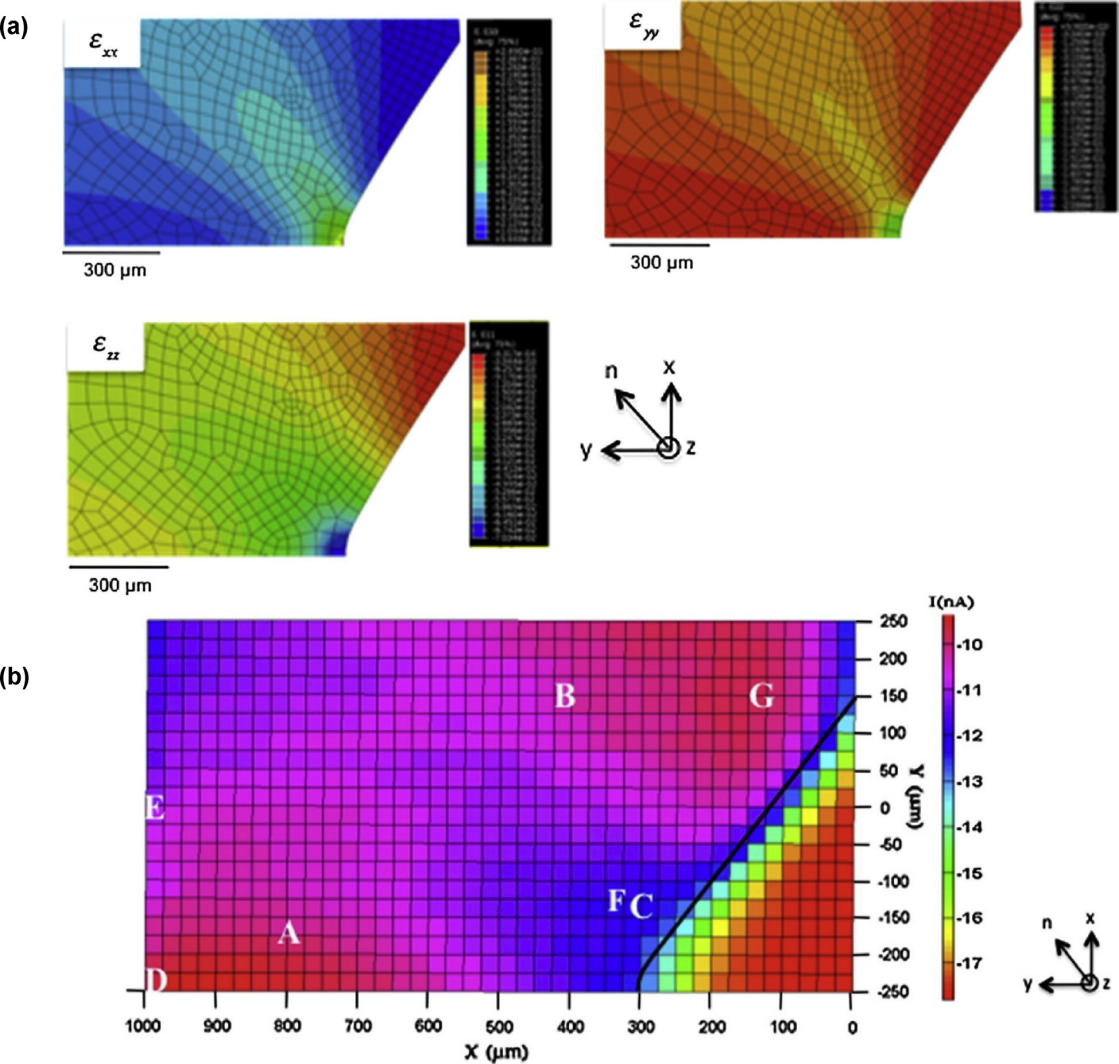
(a) FE modelling showing normal plastic strain at a notch along the x axis, y axis and z axis of a notched 304L stainless steel tensile test specimen; (b) SECM current density map of the notch whilst under tensile stress (470 MPa) in 10 mM K_3_ [Fe(CN_6_)]·3H_2_O + 0.5 M K_2_SO_4_. From Sidane et al. [2014].^34^

Scanning electrochemical microscopy (SECM) is an *in situ* technique capable of direct EAC process measurement. It employs a small polished Pt wire probe encased within an insulator (an ultramicro-electrode). The wire can range in diameter from 10 μm [Bard et al., 1989] down to as small as ~10 nm through the use of modern preparation techniques [Amemiya et al., 2008]. For corrosion experiments, the specimen and tip are both immersed alongside the reference counter electrodes. The SECM probe can be used in two ways. The first, known as collection mode, is suitable for monitoring corrosion processes from sample generation of ionic species, either at a fixed potential or through cyclic voltammetry. The second, feedback mode, in which the tip moves towards the specimen at a rate of the order of 1–5 μm s^−1^ while the current is monitored, allows diffusion processes to be studied [Pust et al., 2008]. To date, SECM has had limited use within the EAC field. Whilst research is limited, 304L austenitic stainless steel has been examined while immersed under static stress conditions [Sidane et al., 2011, 2014]. Electron transfer kinetics of the passive stainless steel oxide layer were determined using feedback mode under elastic stress, and in the unstressed condition [Sidane et al., 2011]. The same material (notched tensile specimen) with stresses applied led to plastic deformation (Figure 36) [Sidane et al., 2014]. A raster scan can be performed at a constant height for imaging purposes using both modes. Spatial resolution is dependent on scan height and tip diameter, with greater resolution attained when nanometre-size tips are used. SECM, therefore, uses stepper motors capable of step sizes of <1 nm [Bard et al., 1989], and is sometimes coupled with AFM to use piezoelectric motor positioning and perform high spatially-resolved topographical maps of the specimen surface [Amemiya et al., 2008].

SECM provides high spatial resolution within the order of nm, if nm-sized tips are used and the specimen-to-tip distance is comparatively small. The feedback mode capability presents an interesting possibility to measure diffusion processes for SCC propagation once cracks initiate but may be limited by crack growth geometry.

#### 3.4.2 Acoustic emission

AE is a non-destructive, *in situ*, direct technique [Du et al., 2011; Kovac et al., 2010] capable of identifying and characterising various EAC processes, *e.g.* uniform, pitting, crevice and erosion corrosion [Kovac et al., 2015]. AE can be used to perform both global and local measurements on a system undergoing corrosion [Calabrese et al., 2015a,b]. Similar to electrochemical techniques, AE signals are reasonably difficult to localise, although the events can be positioned to within a few mm with conventional methods, or more precisely with the use of micro-assemblies. Time series data can be analysed with microsecond resolution. AE has been widely applied to the detection of SCC from acoustic waves emitted by fast energy relaxation events [Calabrese et al., 2015a,b; Kovac et al., 2010; Kovac et al., 2015]. AE has previously been implemented for SCC and cracking mechanism studies in a number of different metals and alloys, such as α-brass [Laptiz et al., 2007], single crystal Ag–10Au alloy [Alvarez et al., 2012], and type 316L and type 304 stainless steels [Alvarez et al., 2008; Cakir et al., 1999].

**Figure 37:**
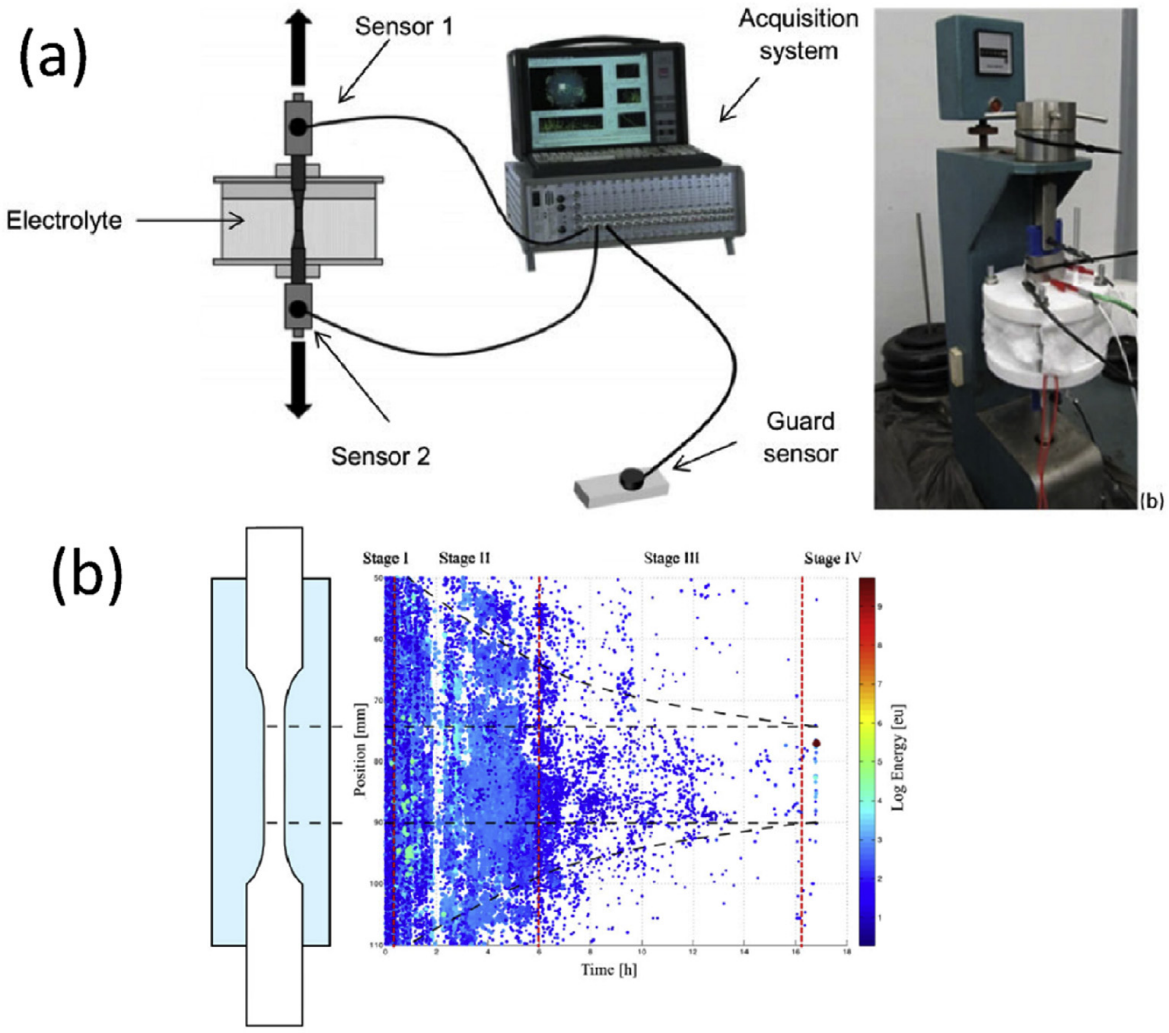
(a) Experimental system used by Calabrese et al. [2015b]^35^ to study SCC using AE; (b) AE event distribution as a function of time; the four distinct damage stages are highlighted.

Calabrese et al., 2015a, Calabrese et al., 2015b were able to identify four distinct damage stages from an AE cumulative energy plot (Figure 37), compared with simultaneous electrochemical measurement (Figure 34): (i) electrochemical activation; (ii) crack initiation and short-range propagation; (iii) long-range crack propagation; and (iv) failure. Figure 37 shows the location of AE sensors at either end of the specimen which was immersed in electrolyte and also results from an AE experiment as a function of distance and energy [Calabrese et al., 2015a, Calabrese et al., 2015b]. Kovač et al. [2015] found considerable differences between AE signals measured from SCC and those from non-crack-related events. Some researchers have used AE in combination with EN to investigate SCC in austenitic and martensitic stainless steels [Calabrese et al., 2017; Du et al., 2011].

AE is capable of identifying single SCC events [Du et al., 2011; Kovac et al., 2010], such as crack initiation and propagation, and it can be used to monitor the evolution of SCC and the development of defects within a material [Calabrese et al., 2015a, Calabrese et al., 2015b; Du et al., 2011; Kovac et al., 2015].

### 3.5 Diffraction and reflectivity techniques

#### 3.5.1 X-ray diffraction

**Figure 38:**
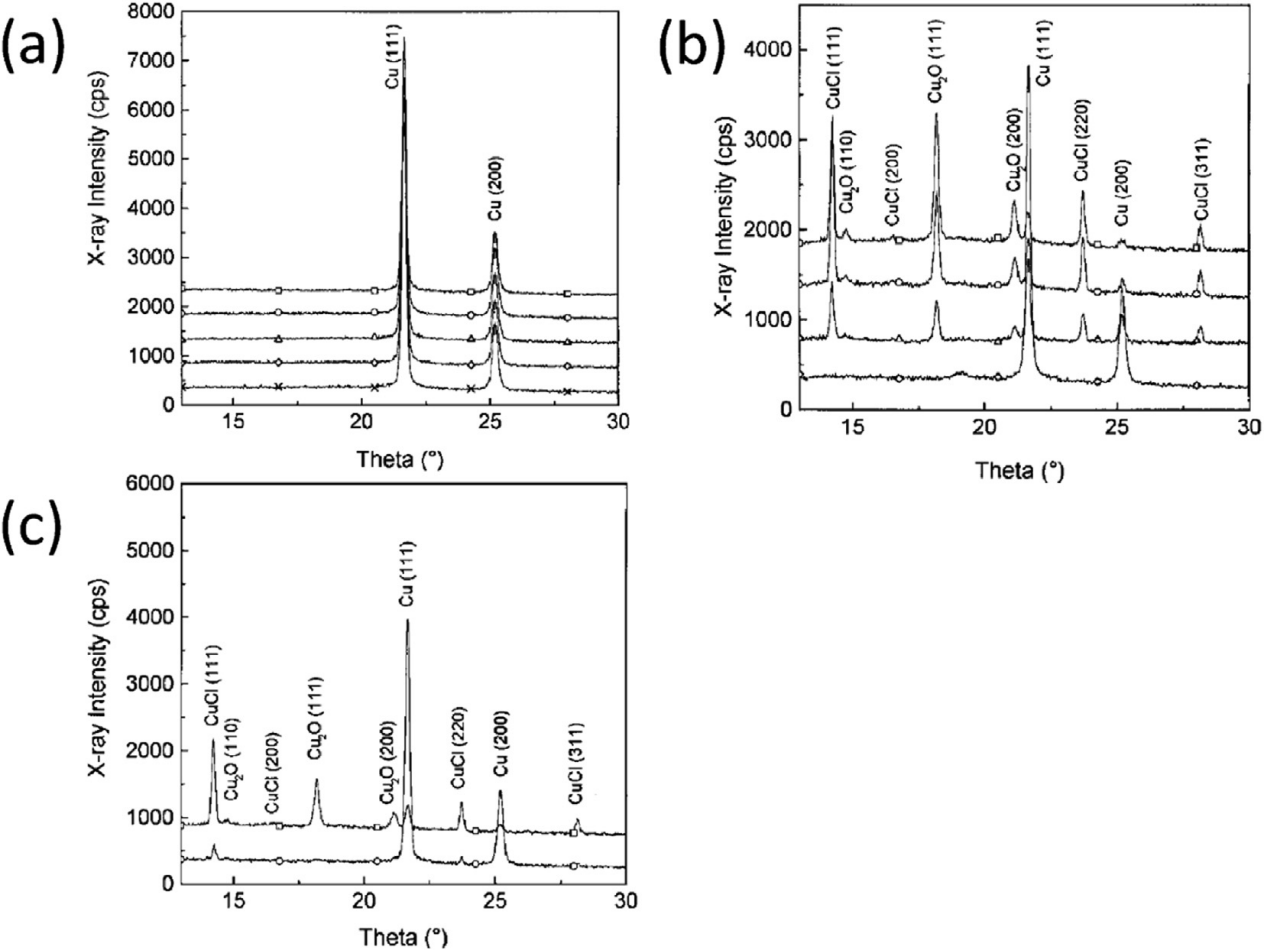
Glancing incidence XRD spectra obtained from copper in 0.1 N NaCl as a function of immersion time for: (a) 0 mV vs SCE; (b) 100 mV vs SCE; (c) 200 mV vs SCE. From Sathiyanarayanan et al. [1999].^36^

X-ray diffraction (XRD) involves interpretation of diffraction patterns observed from the incidence of an X-ray beam onto a crystalline solid specimen [Flewett and Wild, 2017]. The technique allows identification of crystal structure, which is valuable for detailed analysis of corrosion products. XRD can also be used to measure oxide thicknesses, such as the growth of protective Cr_2_O_3_ on austenitic stainless steel in a high-temperature CO_2_ environment [Tempest and Wild, 1982]. The technique also allows crystallographic texture to be deduced. Residual stress fields at the specimen surface may be measured by resolving macroscopic strain from variations in the crystal lattice parameters and micro-strain from peak broadening, although this is limited to elastic strain [Withers and Bhadeshia, 2001]. The technique is generally used *ex situ*, although laboratory X-ray sources can be used for *in situ* purposes. For example, Sathiyanarayanan et al. [1999] used glancing incidence XRD to characterise the structure and growth of oxides on copper exposed to chloride, as shown in Figure 38. Given the limited source brightness in comparison to synchrotron sources, this technique has been classed as an indirect measurement.

#### 3.5.2 Synchrotron X-ray diffraction

High-intensity beam lines have revolutionised materials science due to the capability to make extremely fine, spatially- and temporally-resolved measurements. A number of techniques are relevant to corrosion science, including: X-ray reflectivity; high-angle XRD; X-ray absorption spectroscopy; and X-ray absorption near edge structure (XANES). Kendig et al. [1993] used XANES to study composition and oxide film formation on coated aluminium as part of an industry-wide effort to remove hexavalent chromium (Cr(VI)) from chromate conversion coatings due to its toxicological and environmental impacts. Kendig et al. [1993] noted that, although XPS is a suitable technique to investigate film formation, XANES was potentially better suited to extract the Cr(VI) to total Cr ratio. In ultra-high vacuum, photodecomposition of Cr(VI) occurs, leading to errors in the ratio and inability to evaluate the surface composition of even a few monolayers in air. XANES was used to study film formation as a function of time in a chromating bath, allowing the Cr(VI) to Cr(III) ratio within the coating to be obtained to establish the mechanism by which corrosion protection occurs. Many of these techniques may be applied *in situ*, to provide a direct measure, due in part to the high-intensity, or high penetrating power, of the X-ray beams Weichen et al., 2015a, b. The dual use of a high-intensity X-ray beam for simultaneous irradiation and probing of uranium dioxide in an aqueous environment was demonstrated by Springell et al., 2015.

**Figure 39:**
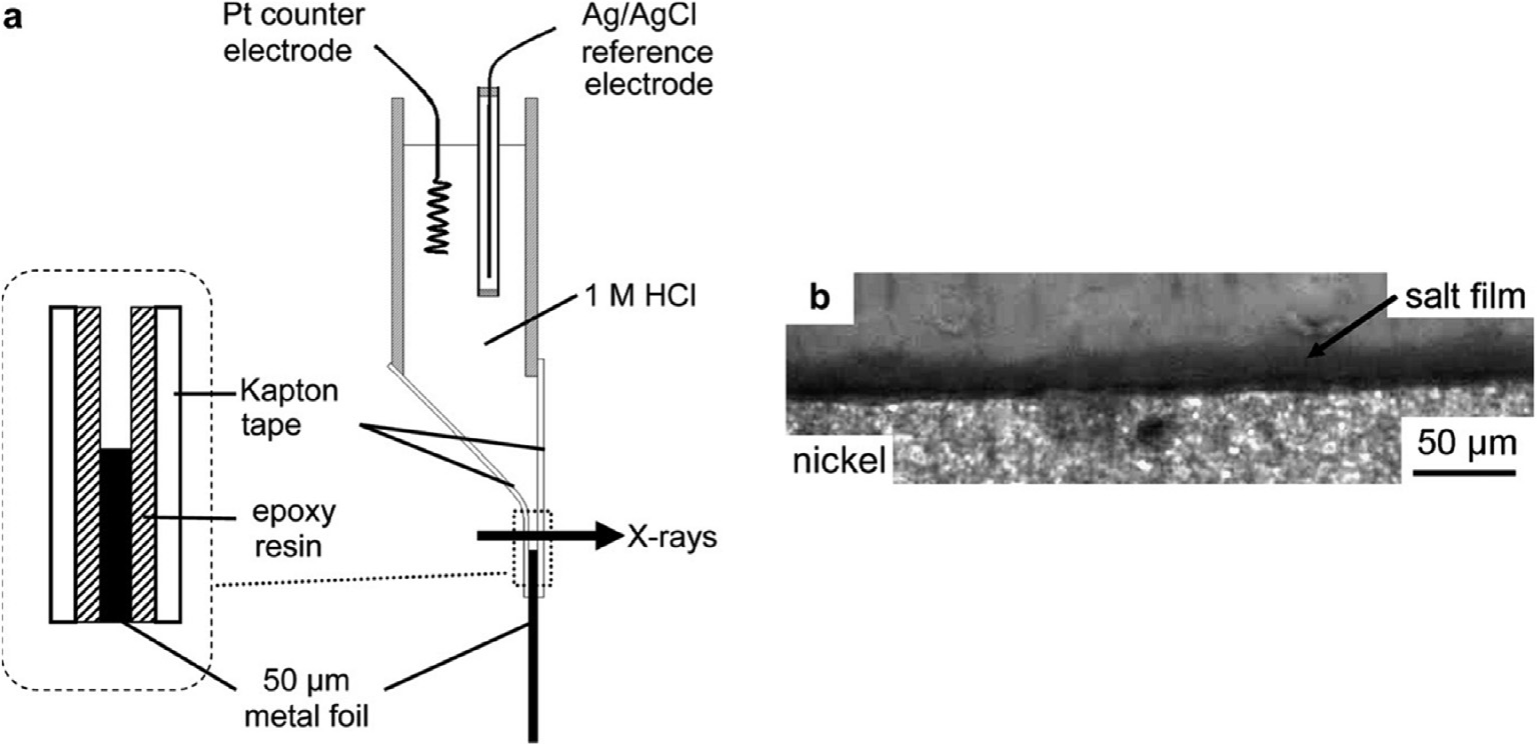
(a) Schematic for the characterisation of salt films on a corroding metal surface using synchrotron XRD applied external polarisation; (b) optical micrograph of a salt film on a nickel surface exposed to HCl. From Rayment et al. [2008].^37^

The formation of salt films on iron, nickel and stainless steels was studied using *in situ* synchrotron XRD (Figure 39). This approach allows the chemistry and crystal structure to be determined at an artificial pit [Rayment et al., 2008]. The authors were able to isolate the main salt phases present on each of the metal systems studied when exposed to HCl. For stainless steel (316L) and iron, FeCl_2_·4H_2_O was found to be the main phase, compared to NiCl_2_·6H_2_O on nickel; the results were found to be consistent with those from the literature.

#### 3.5.3 Neutron diffraction

Neutron diffraction can yield similar structural and crystallographic information as XRD. However, it is much less surface-constrained due to the greater penetration of neutrons [Fitzpatrick and Lodini, 2003]. *In situ* neutron diffraction was used by Chen et al. [2014] to study weld residual stresses and subsequent relaxation following heat treatment on butt-welded ferritic steel pipe, which would not be possible using XRD due to its limited penetration. There is also a greater sensitivity for light elements as the main interaction is nuclear rather than electronic, and isotopic resolution is often available. Small angle neutron scattering is a prominent example which provides information on the distribution of phases within a specimen, with nanometre resolution. These techniques may also be used for residual stress analysis. Neutron diffraction would typically be classed as an *ex situ*, indirect technique, as neutron sources are usually either from a fission reactor or from spallation sources, and experiments are undertaken following slow strain rate testing (SSRT).

### 3.6 Mechanical testing

#### 3.6.1 Small-scale tensile testing

There are a variety of very-well-developed macro-scale mechanical testing techniques involving specimen types including compact tension and single edge notched bending/tensile [Anderson, 2005]. These can generally translate into smaller scales, as micro-compact tension type specimens. Small tensile beams and three- or four-point bend tests are also widely used for the more specialised testing for measuring SCC resistance, for applying well-known stress and strain states to a specimen and allowing dynamic measurements. SSRT and constant extension rate tensile tests as *in situ*, direct techniques are well-developed for studying the effect of tensile stress on a specimen within an aqueous environment [Lopez et al., 1999; Najjar et al., 1997; Puiggali et al., 1998], and can be miniaturised by, for example, the use of ‘dog-bone’ type specimens ASTM, 2009.

#### 3.6.2 Small punch/disc testing

**Figure 40:**
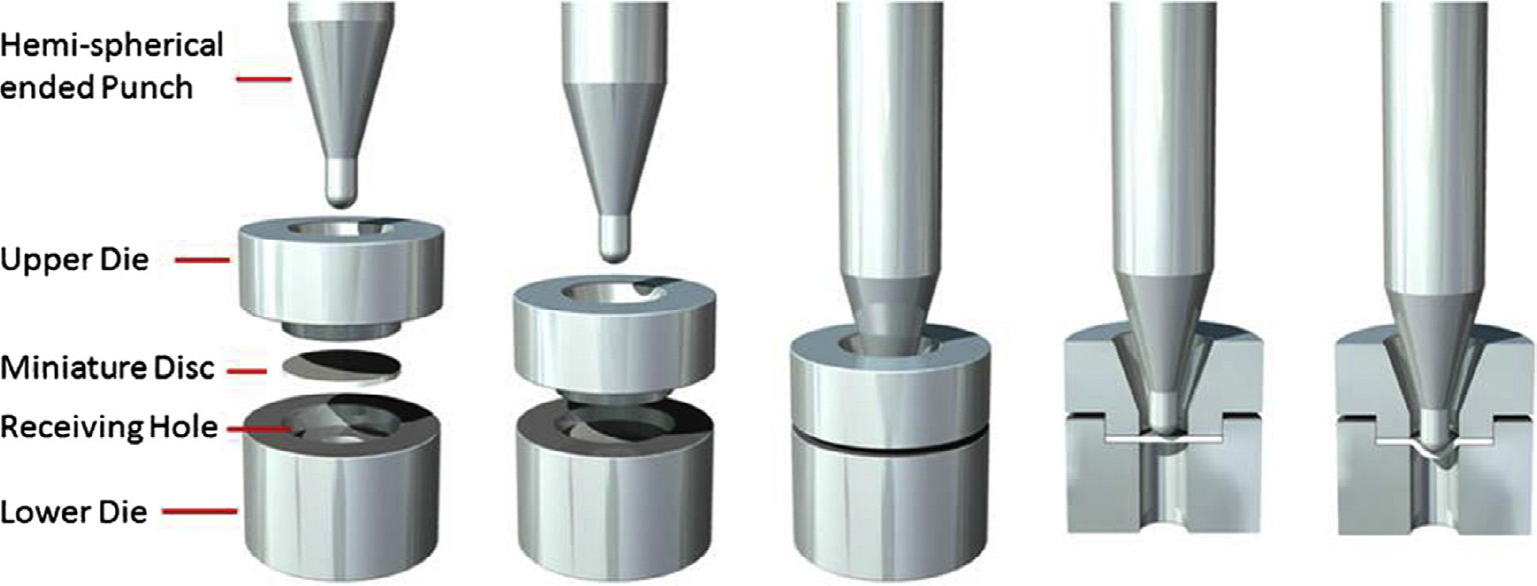
Schematic of the small punch test showing the location of the small punch specimen and upper/lower dies. From Hurst et al. [2016].^38^

**Figure 41:**
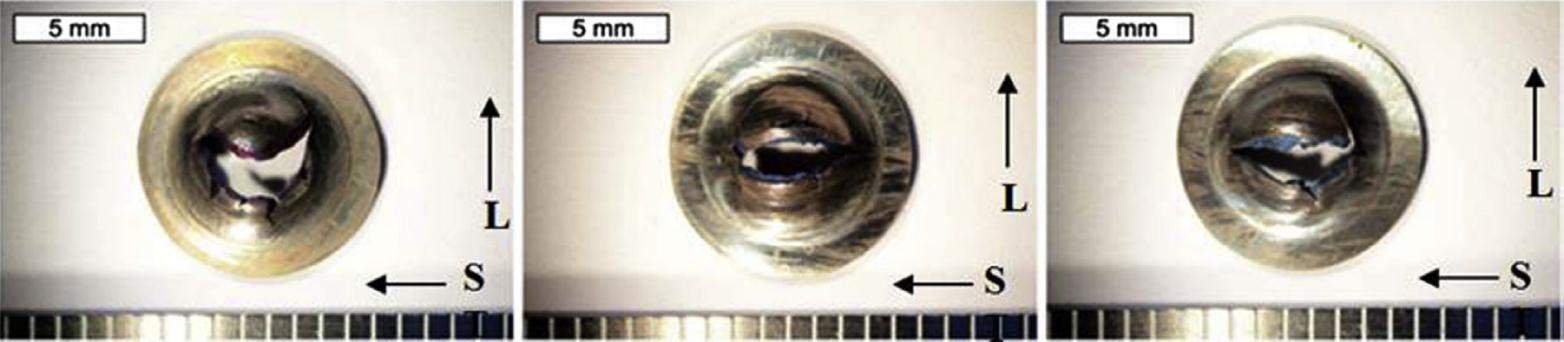
Examples of ALM small punch test specimens following the small punch test. From Hurst et al. [2016].^39^

These small-scale, destructive mechanical tests use equi-biaxial loading (Figure 40) on disc specimens several mm in diameter and normally ~0.5 mm thick. A die contacts the specimen surface and is loaded, ultimately causing deformation which can be measured and related to bulk mechanical properties. Examples of failed specimens are shown in Figure 41. Extensive work programmes have validated the technique for various creep, brittle and ductile fracture [Flewitt, 1995], and fatigue regimes, and notably allowed the use of size-reduced irradiated steel specimens or the examination of additive layer manufactured (ALM) materials. Specimens are typically tested at high temperature, with the loading regime chosen according to the type of degradation process under test [Hurst et al., 2016].

**Figure 42:**
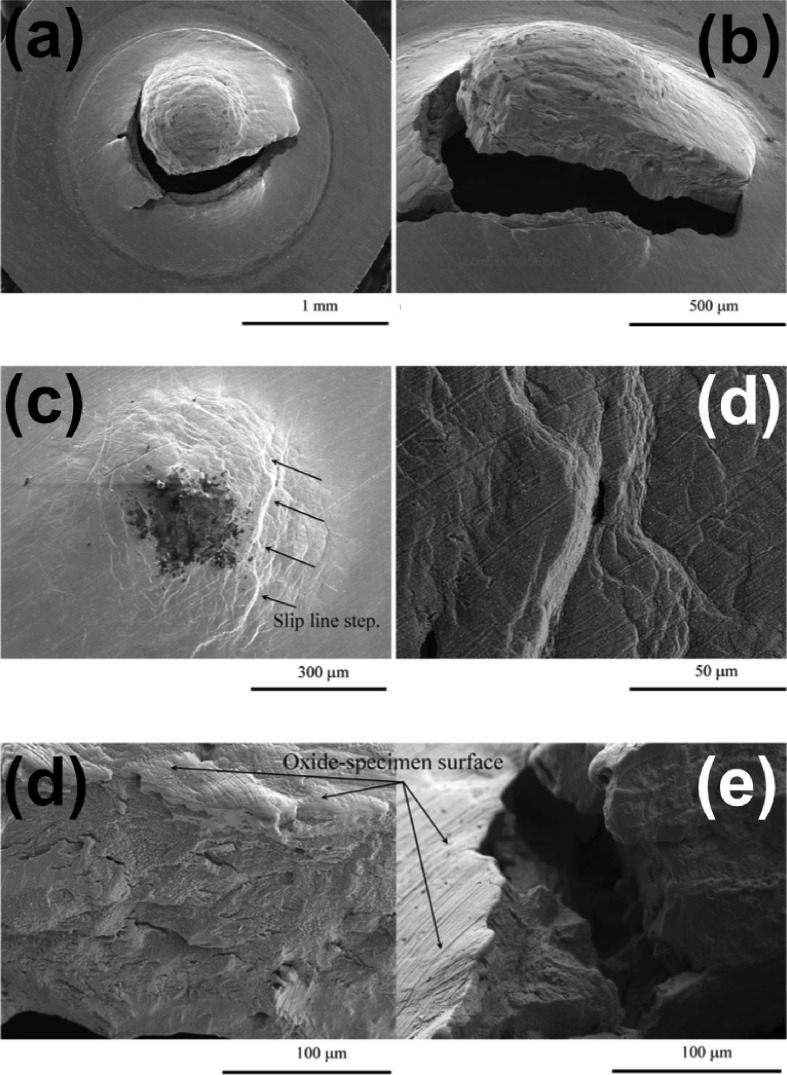
Cold rolled 316L austenitic stainless steel specimens following small punch test in a BWR environment. (a, b) top and side view of the failed specimen, (c) top view slip line step, (d) view of crack initiation point along slip line step, (d) view of the fracture face, (e) crack initiation site. From Isselin et al. [2008].^40^

This method has been developed to allow *in situ* EAC experimentation with chemistry, temperature and pressure conditions representative of a BWR (288 °C; 9 MPa) [Isselin et al., 2008]. This setup is capable of simulating both BWR and PWR conditions and, through the use of a zirconia viewing window, *in situ* observation of type 304L sensitised stainless steel specimens (1.5 mm diameter, 250 μm thick) [Isselin et al., 2005]. Isselin et al., [2008] cold rolled specimens to varying target yield strengths (500, 750, 1000 MPa), prepared, and then subjected to examination in a BWR environment with a gradual load applied. Figure 42 shows the results of this experiment for the 1000 MPa cold rolled austenitic stainless steel specimens [Isselin et al., 2008].

#### 3.6.3 Micro-mechanical methods

**Figure 43:**
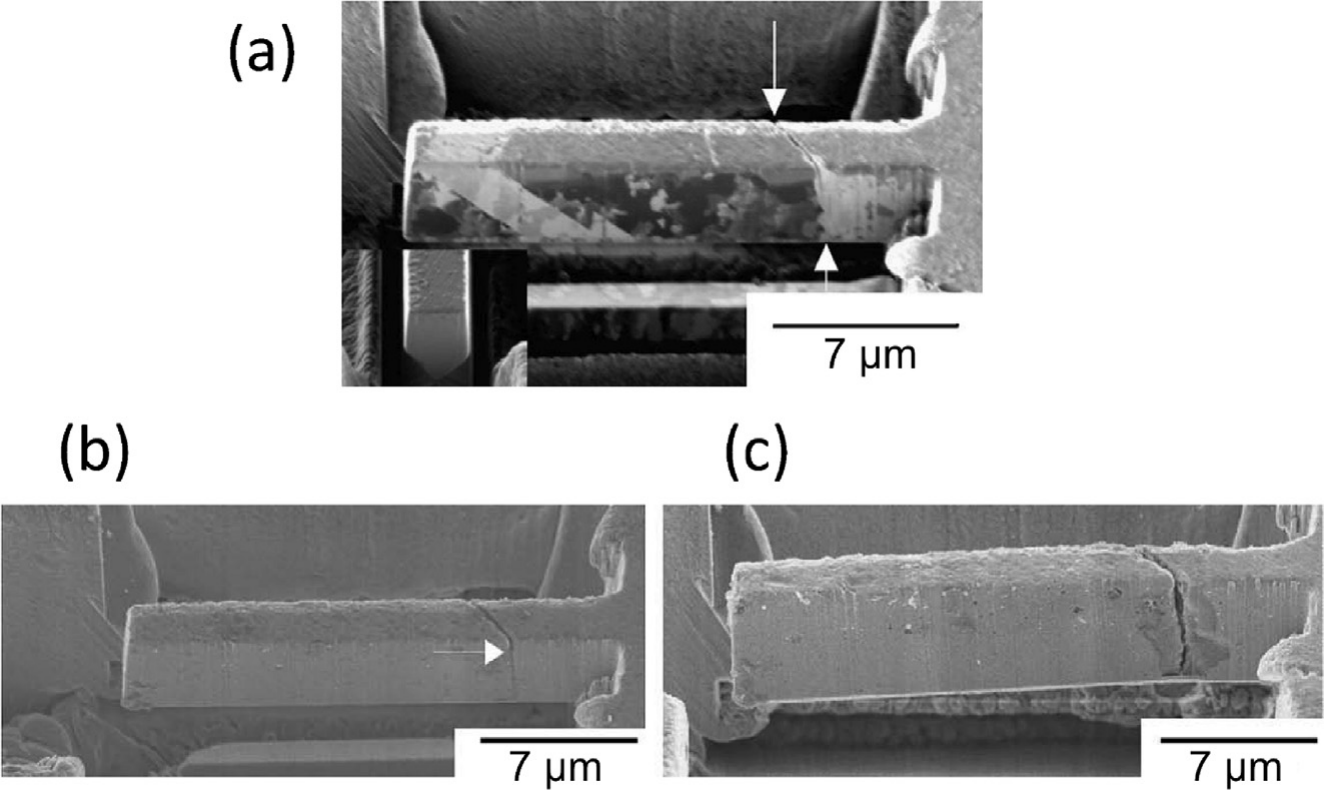
(a) FIB SE image of fabricated cantilever before experiment. The cantilever was then exposed to 0.1 mol potassium tetrathionate; (b) cantilever following 750 μN load, showing a partial crack; (c) cantilever following 775 μN load, which caused failure, as the crack propagated through the beam. From Armstrong et al. [2009].^41^

Relatively recently, with advances in micro-scale techniques, studies in SCC have turned to micro-mechanical methods [Armstrong et al., 2012; Yan et al., 2012]. These take a number of similar forms and can be used to measure mechanical properties on the nano-scale, such as critical stress intensity factor [Stratulat et al., 2016], fracture toughness [Armstrong et al., 2012; Di Maio and Roberts, 2005; Wurster et al., 2012; Darnbrough et al. 2013*.*] or fatigue strength [Yan et al., 2012]. To test for these properties, a micro-cantilever of the material under investigation can be fabricated by means of FIB machining or lithography, and stressed by a nano-indenter until fracture. The experimental system adopted by Armstrong et al. [2009] allows measurements to be conducted by immersion in a corrosive electrolyte (Figure 43).

Errors within this technique may arise from the very small sample size (*i.e.* individual grains) compared to larger specimens, so that it is necessary to establish implications for the micro length-scale. However, micro-mechanical methods for measuring stress intensity factor have been validated on a number of materials, *e.g.* silicon [Di Maio and Roberts, 2005], tungsten [Wurster et al., 2012], bismuth-embrittled copper [Armstrong et al., 2011], tungsten carbide coatings [Di Maio and Roberts, 2005] and electrodeposited nano-crystalline nickel-tungsten films [Armstrong et al., 2012]. Another source of error may arise from the cantilever fabrication, where thin amorphous films of implanted gallium of the order of 10s of nm may be introduced from the ion milling process [Kiener et al., 2007]. New evidence from Hofmann et al. [2017] indicates that in all cases lattice distortions will be caused when using FIB, and if a protective layer is used, such as Pt, damage and substantial strain should be expected. This contamination from the ion beam may affect EAC experiments, which can be minimised to some extent using:

• deposition of a protective layer, such as Pt, W or C.
• reduced ion beam current.
• reduced ion beam current for the final milling stage.

**Figure 44:**
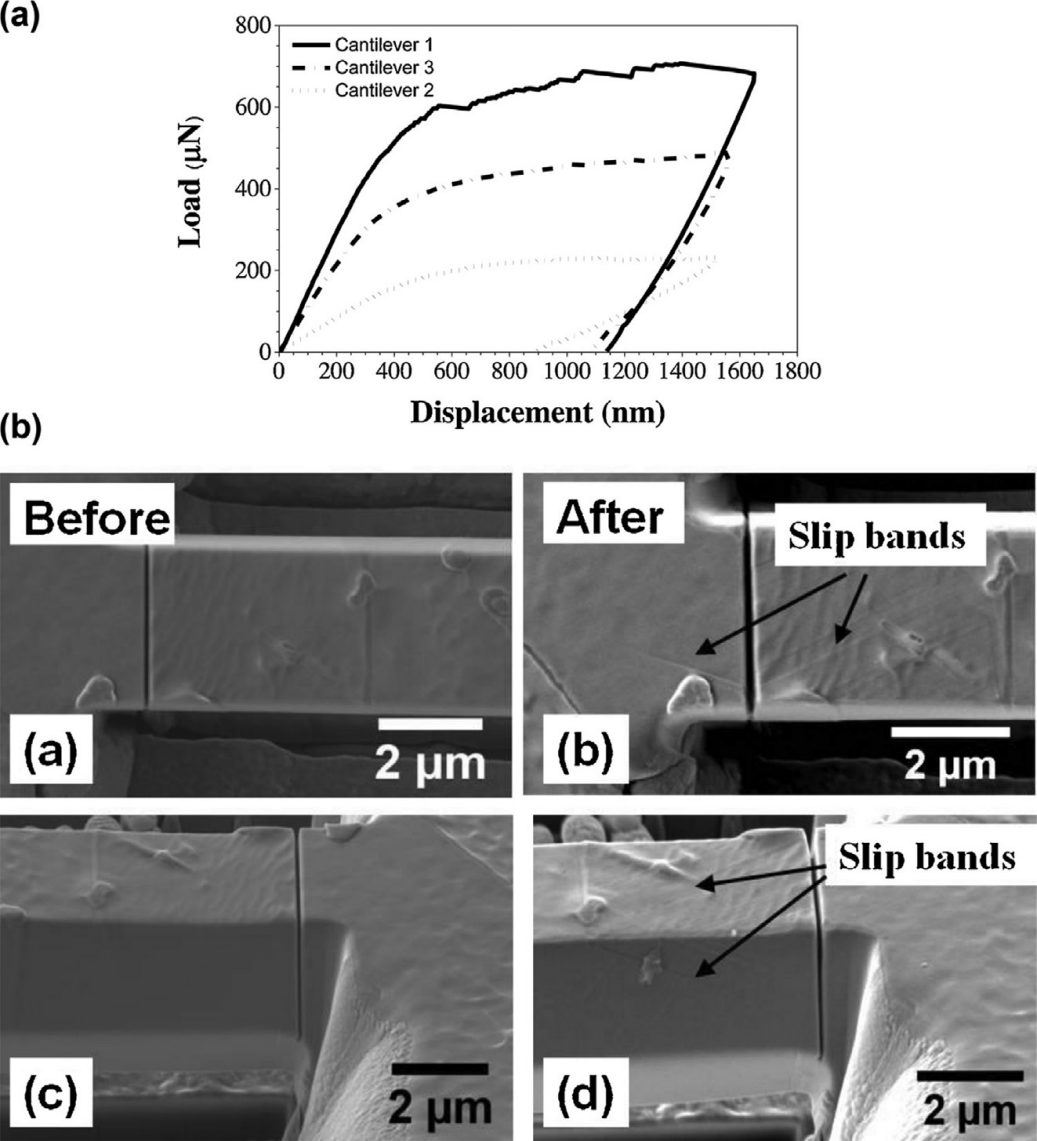
Alloy 600 pentagonal profile micro-cantilevers (25 μm long, 5 μm wide) exposed to a hydrogenated PWR environment at 325°C for 1500 h: (a) stress-strain curves; (b) the specimen before the load was applied; (c) after the load was applied. From Stratulat et al. [2016].^42^

Stratulat et al. [2016] implemented micro-mechanical testing to determine the critical stress intensity factor for fracture along oxidised grain boundaries of Alloy 600 (Figure 44) with varying orientations exposed to PWR primary water chemistry conditions (325°C; hydrogen partial pressure 30 kPa). The micro-cantilevers were gallium ion milled and notched along the grain boundary under investigation. Following exposure to the test environment, the final cantilevers were then stressed by loading the free end using a nano-indenter. It was found that oxidation resulted in a significant weakening of the grain boundaries, providing further insight into the factors and environments that can result in IGSCC susceptibility for nickel-based alloys.

**Figure 45:**
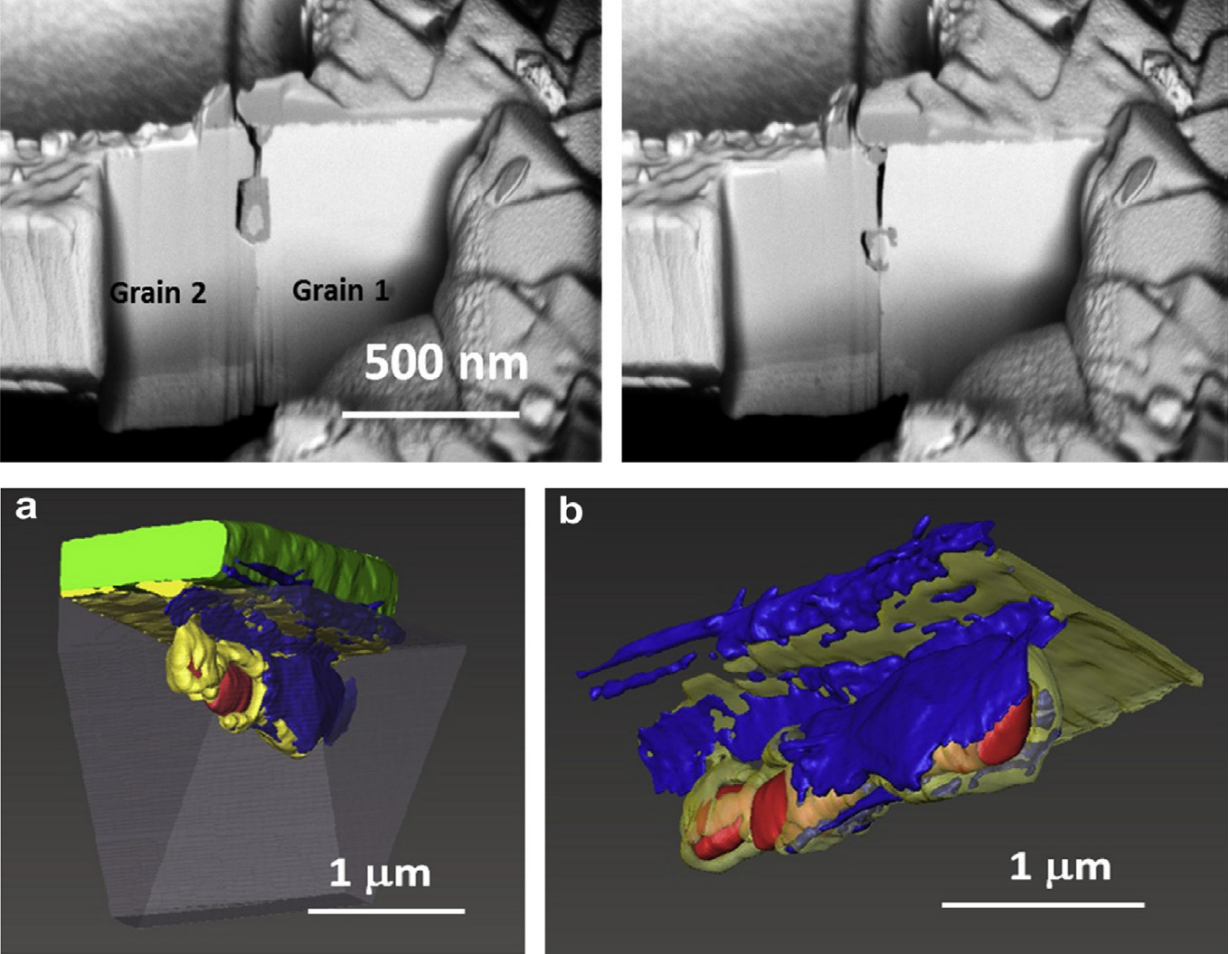
Secondary electron images obtained during FIB serial sectioning of a triangular profile micro-cantilever following exposure to PWR conditions and loading. From Dugdale et al. [2013].^43^

Similarly, Dugdale et al. [2013] exposed triangular profile Alloy 600 micro-cantilevers to PWR chemistry (500 ppm B; 2 ppm Li; 2.75 ppm DH_2_) at 360°C for a period of 2700 h. Following exposure, the micro-cantilevers were loaded to failure. Dugdale et al. [2013] also performed FIB serial sectioning and TEM following exposure and loading. This resulted evaluating how oxidised grain boundaries fail, for instance the path cracks may propagate in the presence of chromium carbides located at grain boundaries. The images in Figure 45 shows crack propagation along the left of a chromium carbide precipitate located on a grain boundary. The upper-left and upper-right images show the cross-section at different sections. The lower-left and lower-right images are 3D models of the micro-cantilever, generated from FIB serial sectioning. Red denotes chromium carbides, yellow Cr-rich oxide, green Fe-rich external oxide and blue the open crack.

**Figure 46:**
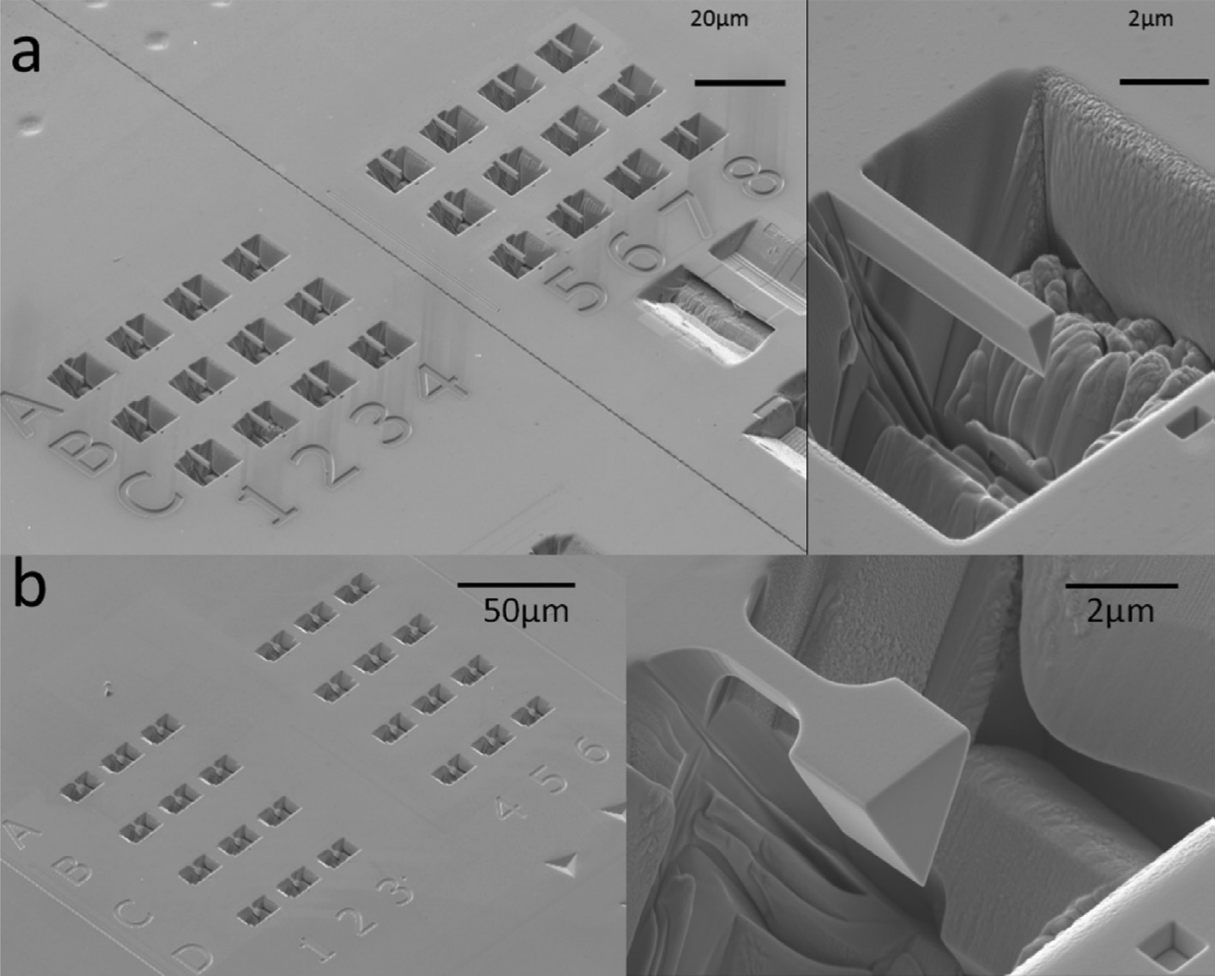
Example SEM images of micro-cantilever arrays fabricated by FIB: (a) uniform cross-section; (b) waisted cross-section. From Armstrong et al. [2015].^44^

**Figure 47:**
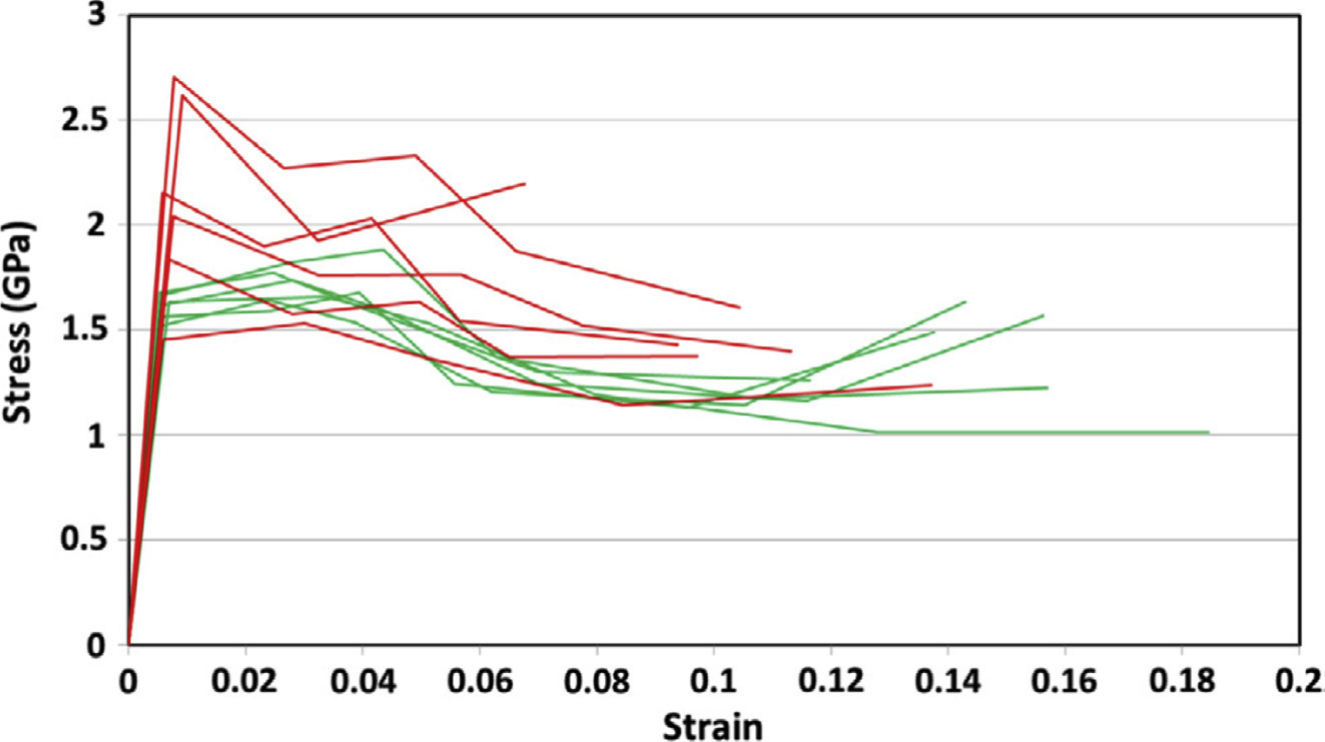
Stress-strain curves for ion-irradiated micro-cantilevers (red) and unirradiated micro-cantilevers (green). From Armstrong et al. [2015].^45^

The work by Armstrong et al. [2015] exemplifies one particular benefit of using such micro-mechanical methodologies, work on irradiated materials. Ion implantation was performed on Fe- 12% Wt. Cr steel. Following this, an array of 12 micro-cantilevers was fabricated on the ion-irradiated and non-irradiated areas of the same grain (example shown in Figure 46). These were then loaded using a nano-indenter and stress-strain curves recorded (Figure 47). The work is an example of how micro-mechanical methods could be used to qualitatively assist with high-throughput EAC testing of novel materials, for future material applications to Gen IV or fusion.

**Figure 48:**
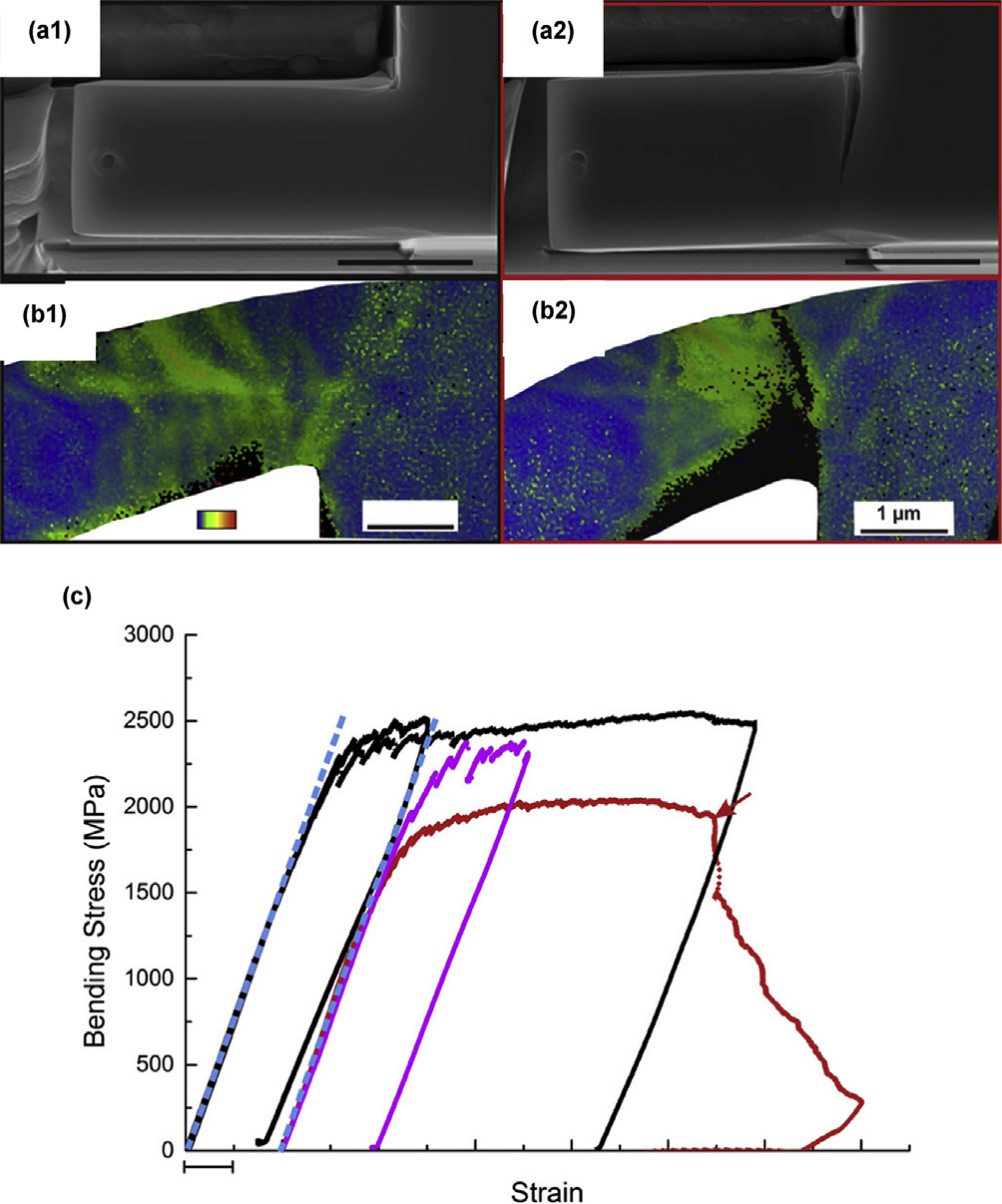
The use of micro-cantilevers to study hydrogen embrittlement within an ESEM in vacuum: (a1) micro-cantilever in top-down view before exposure to water vapour and applied stress; (a2) following exposure and stress; (b1) EBSD KAM map of micro-cantilever, side view, before exposure to water vapour and appied stress; (b2) after exposure and applied stress; (c) stress-strain curve for micro-cantilevers exposed to the hydrogen charging environment. From Deng et al. [2017].^46^

The micro-cantilever technique has been combined with electrochemical environments to explore hydrogen-assisted cracking of ferritic steels by cathodic charging of hydrogen whilst bending *in situ* (Barnoush and Vehoff, 2010; Deng et al., 2017; Hajilou et al., 2017]. Deng et al. [2017] fabricated micro-cantilevers by FIB (dimensions ~2 × 4 × 8 μm) and then moved the specimens into an ESEM to conduct an EAC experiment. Through the use of ESEM, alongside a pico-indenter system, both loading and hydrogen-charging could be conducted, with images taken iteratively. Figure 48 shows how the three main variables for EAC (stress; susceptible microstructure; environment) can be studied using a micro-mechanical approach, alongside advanced EBSD. Figure 48(a1, a2) shows secondary electron images of the micro-cantilever, in top-down view, before and after exposure to water vapour (and applied stress); corresponding EBSD kernel averaged misorientation (KAM) maps are shown in Figure 48(b1, b2), side-view in vacuum. The KAM maps show the average MO for each pixel (represented by the false-colour-scale) compared to neighbouring pixels with respect to a reference point within each grain at a minimum, or zero, strain. Figure 48(c) shows the stress-strain curves for the material in vacuum, and in the presence of water vapour. In the first instance, orientation was established using EBSD and then the micro-cantilevers were stressed *in situ* by the picoindenter system whilst also being exposed to the hydrogen-charging environment (water vapour) within the ESEM. EBSD and TEM were then conducted following the experiments.

Such tests can be applied *in situ*, to provide direct measurement of displacement for a given applied force. This is simple for a system comprised of a material and stress, but without the influence of solution environment chemistry (*i.e.* SCC). For example, whilst there have been inroads to the use of micro- and nano-mechanical testing, such as the use of micro-electromechanical systems, nanowires, nanostructured thin films and more, these do not take into account the environment. Such procedures were reviewed by Gianola and Eberl [2009]. Furthermore, whilst experiments have been conducted on micro-cantilevers *in situ* [Barnoush and Vehoff, 2010; Hajilou et al., 2017), these have not been conducted at temperatures exceeding the boiling point of water. Likewise, experiments have been conducted on micro-cantilevers exposed to PWR conditions by Stratulat et al. [2016], but examined using a micromanipulator following oxidation.

**Figure 49:**
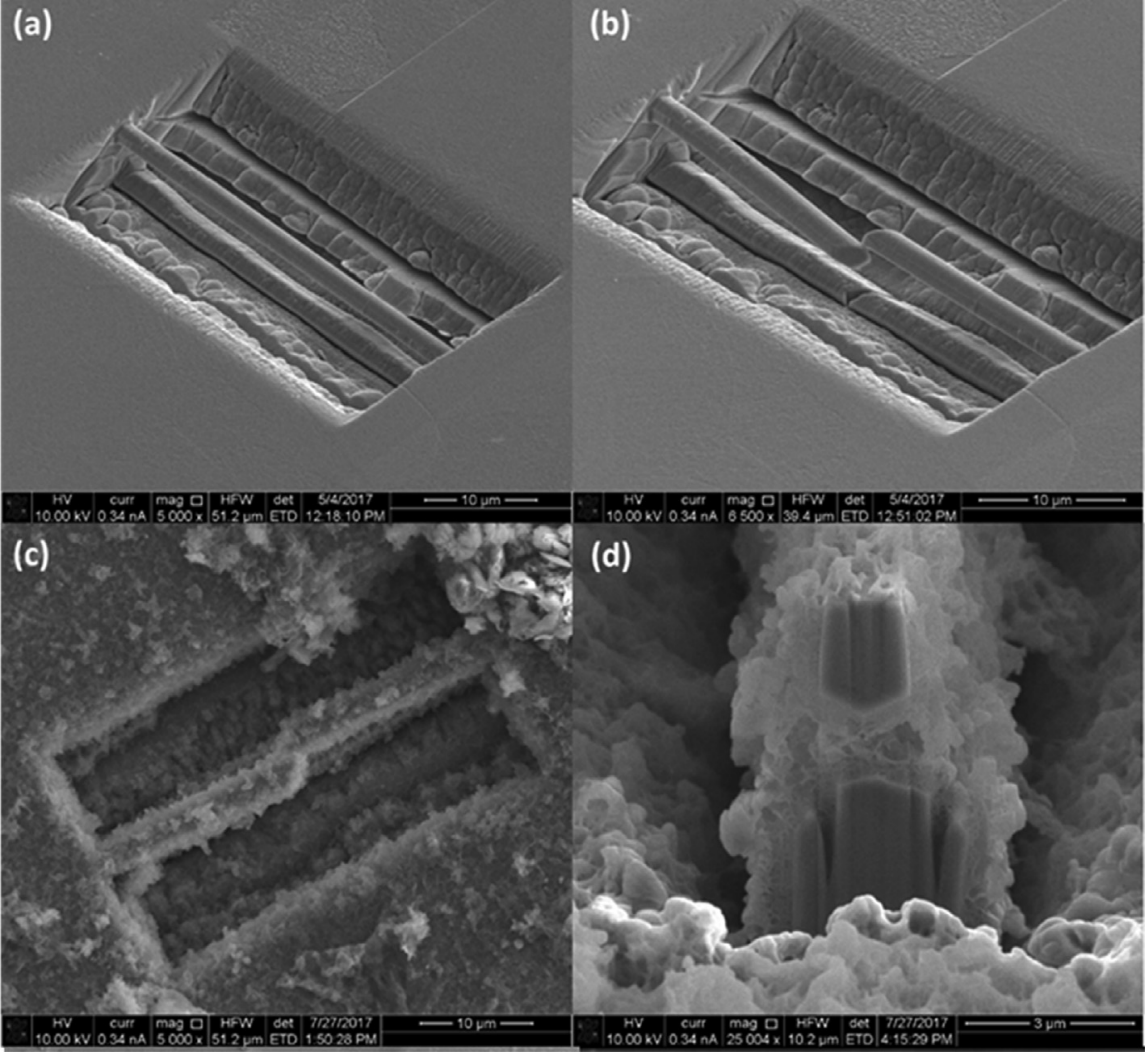
Example of a setup for EAC study on the micro length-scale in high-temperature, high-pressure water conditions: (a) hexagonal profile micro 40 μm length beam; (b) self-stressed micro-cantilevers; (c) top-down view of micro-cantilevers following exposure to how water environment; (d) cross-section of the corroded hexagonal beam following exposure to hot water environment. From Holmes et al. [2018].^47^

One such example of where the EAC variables (high-temperature water; stress; susceptible microstructure) have been combined alongside the micro-mechanical approach is in the work by Holmes et al. [2018]. Figure 49 shows how a single beam was fabricated using FIB, cut in the centre and then displaced through the use of a micromanipulator such that one micro-cantilever underwent plastic deformation and the other still elastic. This resulted in a pair of stressed micro-cantilevers which were then exposed to a high-temperature, high-pressure water environment for EAC studies. Figure 49(a) shows the hexagonal profile beam prepared using FIB, and Figure 49(b) the beam cut in half by FIB. Using a micromanipulator, the authors were able to induce plastic deformation in one cantilever, and checked the other cantilever, which was still within the elastic regime. Figure 49(c) shows the stressed micro-cantilevers following immersion within a 200 ml hot water autoclave (ultrapure water; 200 °C; 230 bar; 15 h). Following exposure, the micro-cantilevers appeared to be fused together by corrosion product, with no cracking evident. A FIB cross-section of the corroded beam revealed that the cantilever was no longer free-standing due to growth of the corrosion product (Figure 49(d)). The work by Holmes et al. [2018] is a possible avenue for further research, but optimisation needs to take place to ensure that the micro-cantilevers are of the right size and geometry for use in high-temperature corrosive environments. Furthermore, questions remain. What is the impact of EAC testing on this length-scale and how can this impact be quantified for comparison to larger length-scales? We have already discussed the work of Hofmann et al. [2017] and Kiener et al. [2007], which need to be considered for future work using the FIB technique for micro-scale EAC cantilever experiments. How representative are the very small sample volumes used (~100 s μm^2^), compared to a larger sample of the material used in conventional EAC testing (10 s cm^2^). Given the smaller volume, there is both the potential for a lower dislocation density as the volume has reduced and also a greater probability that the small volume selected for measurement will not be representative due to the highly-local measurement. For instance, in these micro-mechanical experiments only one or two grains may be examined, but in a standard EAC experiment tens of thousands of grains may be present.

What is required, therefore, is a selection of techniques across multiple length-scales. Only then will this allow the material scientist to understand the pre-initiation, initiation, short crack and long crack stages so vital in underpinning the EAC mechanism at play.

## 4 Discussion

### 4.1 Understanding processes

Application of a range of techniques has significantly enhanced the understanding of EAC; helping develop the mechanisms identified in Figure 4. Due to the complexity of EAC (outlined in Figure 1) the research has also shown the importance of using techniques to focus on the relevant length and time scales (which may not be known *a priori*). The phenomena occurring at crack tips are extremely important, and no single technique is capable of providing all the information required – but used in combination development of a detailed mechanistic understanding is possible.

This section reflects on opportunities for further research and development in EAC testing to gain a better mechanistic understanding of corrosion and materials, and, ultimately, to accurately predict the performance of components and structures. The techniques listed here, in general, span the nano-to-micro length-scale, important for linking to the nanometre length-scale research in EAC. To achieve this it is important to identify and measure the correct parameters associated with the local mechanism that is taking place at the crack tip (Figure 4). The different parameters of interest, dependant on the local mechanism, can be identified and measured by a range of direct and indirect techniques, to gather relevant data to monitor and/or analyse EAC, and also for input parameters in predictive EAC models. Figure 5 shows these techniques grouped together as direct and indirect in accordance to length-scale measured and time-scale for measurement. Due to the relatively large body of work, it is clear from this appraisal that, from the many local mechanisms that have been proposed to describe EAC and the large number of techniques available, there are opportunities in ensuring that:

• the most relevant parameters are identified and measured for that particular corrosion mechanism.
• the techniques have sufficient capability to measure and monitor the relevant parameters:
  o techniques could be improved.
  o new techniques may need to be developed.
• the data generated are used effectively in:
  o analysing SCC.
  o monitoring SCC.
  o SCC models.
• development and improvement of computer models is undertaken to predict SCC.
• new revolutionary methods and approaches to predicting SCC are developed.

By considering correspondences between the direct and indirect techniques and EAC processes, promising areas of focus for development may be identified:

• improvements or advancements of existing techniques to greater time- or length-scale resolution, which expand capability sufficiently to apply to a different set of key processes.
• translating indirect techniques into direct techniques, to achieve the key requirement of *in situ* dynamic observation.
• finding novel or developing techniques, including those which could be transferred from other fields, not previously applied to material or corrosion science.
• combining techniques for use in tandem, to provide correlated data.
• expanding the applicability of techniques which have previously been used to study EAC to investigate specific mechanisms that may have practical constraints, such as involving specimens with radioactive inventories.

Only by consulting the EAC toolbox of techniques available to the material scientist is one able to find a selection of techniques most suitable for measurement, taking into account time- and length-scales. These approaches are not sophisticated and have driven much of the development of observation and characterisation methods which now abound, although it is considered helpful that they should be clearly proposed and their relevance to EAC outlined, given the current diversity and rapidity of technique development. Consequently, from the relatively broad consideration undertaken within the course of this appraisal, some initial specific opportunities are evident, which are outlined below.

### 4.2 Length-scale challenges

It is within the pre-initiation and initiation EAC processes that the greatest potential for advances is perceived to lie, due in part to the great difficulty in making meaningful observations at the time- and length-scales involved. With few exceptions, well-established characterisation techniques are under continuous development, notably for improved spatial resolution and reduced imaging time, offering incremental increases in capability for the study of EAC. One example, better described as a step-change improvement, is HS-AFM, which offers significantly enhanced imaging speed and area coverage. The extent of these developments is such that this technique can now be considered fast enough to image initiation events, but also with sufficiently good automation and post-processing to be able to realistically identify the occurrence of these events over a representative surface area, and to examine aspects of short crack growth.

More generally, a number of approaches have been reported for investigating the local distribution of reaction kinetics (EC-STM; HS-AFM; micro-capillary; SVET; micro-electrode EN), and these have already demonstrated key mechanistic aspects. Further refinement of these methods and their application, particularly to allow more general observations, is recommended.

The use of micro-mechanical testing is very relevant to evaluating mechanisms, such as nuclear industry EAC challenges, RIS and irradiation-assisted EAC, due to the applicability to size-reduced specimens. One specific example for this is within the nuclear industry, where radioactive inventory is a practical constraint. Dose rate is not the only driver towards smaller test-scales for irradiated specimens as, for material development, production of these specimens, for example by exposure in test reactors, is very costly and time-consuming. Although micro-mechanical testing can be used to look at the *ex situ* properties of phases produced by environmental exposure (*i.e.* oxidised grain boundaries), there is the opportunity to identify means of mechanically-loading these features *in situ*, either with an external manipulator or by introducing a self-loading mechanism into the cantilever design.

There are now various very high-resolution tools for location-sensitive compositional analysis, including *in situ* methods and quite advanced *ex situ* techniques, capable of providing dynamic information. NanoSIMS provides high resolution compositional information (with high spatial resolution) and is able to provide information on trace elements. In order to successfully characterise EAC mechanisms, a combination of methods should be used, for example NanoSIMS to provide compositional information with high sensitivity, TEM to provide structural and crystallographic information, and post-exposure APT for very high resolution elemental profiling. Only by using a combination of techniques can the material scientist obtain the level of detail required to understand the mechanisms for EAC.

Considering the above points together would suggest that a combination of direct and indirect methods, such as *in situ* dynamic measurements on self-loaded micro-mechanical features alongside indirect compositional characterisation, could bring a breakthrough in the understanding of SCC pre-initiation/initiation.

Additionally, the challenges of characterisation at the nano-scale do not apply solely to materials and corrosion science. Indeed, a large number of technique developments have been translations from other disciplines, notably biological sciences. It is suggested that this trend will continue and that there will be benefit in maintaining a watching brief and, specifically, seeking methods being developed in diverse fields which could be transferred key issues such as short to long crack transition.

It is increasingly important to understand the processes from short crack to long crack. Similar to the multi-legged approach outlined for the study of pre-initiation/initiation, it is suggested that a comparable combination of direct and indirect characterisation, linked with well-controlled mechanically-stressed specimen staging at an appropriate scale, should facilitate rewarding research. The use of small disc (equi-biaxial) and small tensile test specimens extends the observation to multiple phase boundaries, rather than the binary systems normally accessible to micro-mechanical methods. A scaled-up *in situ* approach, for example employing microtomography, will be ably suited for integration with more conventional techniques, notably those probing local electrochemistry (*e.g.* SVET) and chemical composition. The long crack regime represents a stable system, in which the interaction of environment and stress produce conditions for crack tip propagation. It is suggested that a worthwhile focus would be on establishment of this stable system using a set-up which would allow for discreet study of crack tip regions and crack sides. Parameters relating local processes to crack extension mechanisms could then be investigated. The short-term processes should be informed by short crack growth mechanisms. Some of these can be applied directly but the processes are much less spatially constrained, so it is significantly more difficult to undertake analyses on the actual active area. The considerable body of work which exists for long crack growth presents a great advantage if study in this area can be linked with short crack techniques, as it provides a pre-existing data set for validation.

## Declarations

### Author contribution statement

All authors listed have significantly contributed to the investigation, development and writing of this article.

### Funding statement

This work was supported by the UK National Nuclear Laboratory Limited.

### Competing interest statement

The authors declare no conflict of interest.

### Additional information

No additional information is available for this paper.

## Footnotes

1 An important additional variable, time-dependence, is often neglected, or under-recognised in SCC investigations, and should be considered. Several processes such as stresses (swelling and relaxation), local water chemistries (radiolysis) and neutron dose accumulation are not static and vary with time. Additionally, laboratory test durations are much shorter than component lifetimes [Andresen and Was, 2019].

2 Reprinted from Journal of Nuclear Materials, 498, Whillock G. O. H., Hands B. J., Majchrowski T. P., Hamley D. I., Investigation of thermally sensitised stainless steels as analogues for spent AGR fuel cladding to test a corrosion inhibitor for intergranular stress corrosion cracking, pp. 187–198, Copyright (2018), with permission from Elsevier.

3 Reprinted by permission from Springer Nature: Springer eBook, Environmentally Assisted Cracking of Irradiated Metals and Alloys, Was G. S. Copyright (2007).

4 The indicative figures quoted typically relate to theoretical resolution – which can be extremely difficult to meet in practice e.g. although APT identifies the location of individual atoms, in real systems the resolution limit is ~0.5 nm not 10^−11^ m.

5 HS-AFM is capable of nanometre scale lateral spatial resolution and subatomic height resolution.

6 HS-AFM is capable of nanometre scale lateral spatial resolution and subatomic height resolution.

7 Reprinted from Corrosion Science, 85, Stratulat A., Duff J. A., Marrow T. J., Grain Boundary Structure and Intergranular Stress Corrosion Crack Initiation in High Temperature Water of a Thermally Sensitised Austenitic Stainless Steel, Observed In Situ, pp. 428–435, Copyright (2014), with permission from Elsevier.

8 Reprinted from Springer Nature: Experimental Mechanics, Scanning Electron Microscopy for Quantitative Small and Large Deformation Measurements Part I: SEM Imaging at Magnifications from 200 to 10,000, Sutton M. A., Li N., Joy D. C., Reynolds A. P., Li X., Copyright (2007).

9 Reprinted from Corroion Science, 52(6), Leiva-Garcia, R., Garcia-Anton, J., Munoz-Portero, M. J., Contribution to the Elucidation of Corrosion Initiation through Confocal Laser Scanning Microscopy, pp. 2133–2142, Copyright (2010), with permission from Elsevier.

10 Previously unpublished figures courtesy of Professor James Marrow, Department of Materials, University of Oxford, UK.

11 Reprinted from Micron, 75, Meisnar M., Vilalta-Clemente A., Gholinia A., Moody M., Wilkinson A., Huin N., Lozano-Perez S., Using Transmission Kikuchi Diffraction to Study Intergranular Stress Corrosion Cracking in Type 316 Stainless Steels, pp. 1–10, Copyright (2015), with permission from Elsevier.

12 Obtained through the Attribution 3.0 Unported (CC BY 3.0) licence. The full details of the license are available at: https://creativecommons.org/licenses/by/3.0/.

13 Reprinted from Corrosion Science, 50(8), Chen J., Wang J., Han E., Ke W., In Situ Observation of Crack Initiation and Propagation of the Charged Magnesium Alloy under Cyclic Wet-Dry Conditions, pp. 2338–2341, Copyright (2008) with permission from Elsevier.

14 Reprinted from Journal of Nuclear Materials, 408(3), Lozano-Perez S., Rodrigo P., Gontard L., Three-Dimensional Characterization of Stress Corrosion Cracks, pp. 289–295, Copyright (2011), with permission from Elsevier.

15 Reprinted from Journal of Nuclear Materials, 408(3), Lozano-Perez S., Rodrigo P., Gontard L., Three-Dimensional Characterization of Stress Corrosion Cracks, pp. 289–295, Copyright (2011), with permission from Elsevier.

16 Reprinted from Corrosion Science, 165, Clark R. N., Searle J., Martin T. L., Walters W. S., Williams G., The Role of Niobium Carbides in the Localised Corrosion Initiation of 20Cr–25Ni–Nb Advanced Gas-cooled Reactor Fuel Cladding, Copyright (2020), with permission from Elsevier.

17 Reprinted from Corrosion Science, 165, Clark R. N., Searle J., Martin T. L., Walters W. S., Williams G., The Role of Niobium Carbides in the Localised Corrosion Initiation of 20Cr–25Ni–Nb Advanced Gas-cooled Reactor Fuel Cladding, Copyright (2020), with permission from Elsevier.

18 In Situ Imaging of Corrosion Processes in Nuclear Fuel Cladding, Laferrere A., Burrows R., Glover C., Clark R. N., Payton O., Picco L., Morre S., Williams G., Corrosion Engineering, Science and Technology (The International Journal of Corrosion Processes and Corrosion Control), 52(8), Copyright © 2017 Institute of Materials, Minerals and Mining, reprinted by permission of Informa UK Limited, trading as Taylor & Francis Group, www.tandfonline.com on behalf of Institute of Materials, Minerals and Mining.

19 Reprinted from Electrochimica Acta, 54(2), Seyeux A., Maurice V., Klein L. H., Marcus P., Initiation of Localized Corrosion at the Nanoscale by Competitive Dissolution and Passivation of Nickel Surfaces, pp. 540–544, Copyright (2008), with permission from Elsevier.

20 Obtained through the Attribution 4.0 International (CC BY 4.0) licence. The full details of the license are available at: http://creativecommons.org/licenses/by/4.0.

21 Reprinted from Journal of Nuclear Materials, 374, Lozano-Perez S., Kilburn M. R., Yamada T., Terachi T., English C. A., Grovenor C. R. M., High-Resolution Imaging of Complex Crack Chemistry in Reactor Steels by NanoSIMS, pp. 61–68, Copyright (2008), with permission from Elsevier.

22 Reprinted from Engineering Fracture Mechanics, 76(5), Turnbull A., Horner D. A., Connolly B. J., Challenges in Modelling the Evolution of Stress Corrosion Cracks from Pits, pp. 633–640, Copyright (2009), with permission from Elsevier.

23 Reprinted from Engineering Fracture Mechanics, 76(5), Turnbull A., Horner D. A., Connolly B. J., Challenges in Modelling the Evolution of Stress Corrosion Cracks from Pits, pp. 633–640, Copyright (2009), with permission from Elsevier.

25 Reprinted from Scripta Materialia, 62, Lozano-Perez S., Saxley D. W., Yamada T., Terachi T., Atom-Probe Tomography Characterization of the Oxidation of Stainless Steel, pp. 855–858, Copyright (2010), with permission from Elsevier.

24 Reprinted from Journal of Nuclear Materials, 408(3), Lozano-Perez S., Rodrigo P., Gontard L., Three-Dimensional Characterization of Stress Corrosion Cracks, pp. 289–295, Copyright (2011), with permission from Elsevier.

26 Reprinted from Electrochimica Acta, 109, Williams G., Dafydd H. ap L., Grace, R., The Localised Corrosion of Mg Alloy AZ31 in Chloride Containing Electrolyte Studied by a Scanning Vibrating Electrode Technique, pp. 489–501, Copyright (2013), with permission from Elsevier.

27 Reprinted from Electrochimica Acta, 109, Williams G., Dafydd H. ap L., Grace, R., The Localised Corrosion of Mg Alloy AZ31 in Chloride Containing Electrolyte Studied by a Scanning Vibrating Electrode Technique, pp. 489–501, Copyright (2013), with permission from Elsevier.

28 Republished with permission of Faraday Division, Royal Society of Chemistry (Great Britain), from In Situ Monitoring of Corrosion Mechanisms and Phosphate Inhibitor Surface Deposition during Corrosion of Zinc–Magnesium-Aluminium (ZMA) Alloys using Novel Time-Lapse Microscopy, Sullivan J., Cooze N., Gallagher C., Lewis T., Prosek T., Thierry D., Faraday Discussions, 180, pp. 361–379, 2015; permission conveyed through Copyright Clearance Center, Inc.

29 Reprinted from Corrosion Science, 57, Williams G., Geary, S. McMurray, H. N., Smart Release Corrosion Inhibitor Pigments based on Organic Ion-Exchange Resins, pp. 139–147, Copyright (2012), with permission from Elsevier.

30 Reprinted from Corrosion Science, 114, Glover C. F., Richards C., Baker J., Williams G., McMurray H. M., In-Coating Graphene Nano-Platelets for Environmentally-Friendly Corrosion Protection of Iron, pp. 169–172, Copyright (2017), with permission from Elsevier.

31 Reprinted from Corrosion Science, 55, Breimesser M., Ritter S., Seifert H.-P., Virtanen S., Suter T., Application of the Electrochemical Microcapillary Technique to Study Intergranular Stress Corrosion Cracking of Austenitic Stainless Steel on the Micrometre Scale, pp. 126–132, Copyright (2012), with permission from Elsevier.

32 Reprinted from Corrosion Science, 98, Calabrese L., Bonaccorsi L., Galeano M., Proverbio E., Pietro D. D., Cappuccini F., Identification of Damage Evolution during SCC on 17-4 PH Stainless Steel by Combining Electrochemical Noise and Acoustic Emission Techniques, pp. 573–584, Copyright (2015), with permission from Elsevier.

33 Reprinted from Nace Corrosion 2018 conference, Paper C2018-11196, Clark, R. N., Knapp J., Laferrere A., Moore S., Payton O., Picco L., Walters S., Burrows R., 2018, Development of an Adapted Electrochemical Noise Technique for In-Situ Corrosion Monitoring of Spent Nuclear Fuel Aqueous Storage Environments, Copyright (2018), with permission from the National Association of Corrosion Engineers.

34 Reprinted from Corrosion Science, 87, Sidane D., Touzet M., Devos O., Puiggali M., Lariviere J. P., Guitard J., Investigation of the Surface Reactivity on a 304L Tensile Notched Specimen Using Electrochemical Microscopy, pp. 312–320, Copyright (2014), with permission from Elsevier.

35 SCC damage evolution on martensitic stainless steel by using acoustic emission technique, Calabrese L., Bonaccorsi L., Proverbio E., Pietro D. D., Cappuccini F., Corrosion Engineering, Science and Technology (The International Journal of Corrosion Processes and Corrosion Control), 50, 5, pp. 364–371, Copyright © 2015 Institute of Materials, Minerals and Mining, reprinted by permission of Informa UK Limited, trading as Taylor & Francis Group, www.tandfonline.com on behalf of Institute of Materials, Minerals and Mining.

36 Reprinted from Corrosion Science, 41(10), Sathiyanarayanan, S., Sahre, M., Kautek, W., 1999, In-Situ Grazing Incidence X-Ray Diffractometry Observation of Pitting Corrosion of Copper in Chloride Solutions, pp. 1899–1909, Copyright (1999), with permission from Elsevier.

37 Reprinted from Electrochemistry Communications, 10(6), Rayment T., Davenport A. J., Dent A. J., Tinnes J-P, Wiltshire R. J. K., Martin C., Clark G., Quinn P., Mosselmans J. F. W. Characterisation of Salt Films on Dissolving Metal Surfaces in Artificial Corrosion Pits via In Situ Synchrotron X-Ray Diffraction, pp. 855–858, Copyright (2008), with permission from Elsevier.

38 Obtained through the Attribution 4.0 International (CC BY 4.0) licence. The full details of the license are available at: http://creativecommons.org/licenses/by/4.0.

39 Obtained through the Attribution 4.0 International (CC BY 4.0) licence. The full details of the license are available at: http://creativecommons.org/licenses/by/4.0.

40 Reprinted by permission from Springer Nature: Metallurgical and Materials Transactions A, Assessment of the Effects of Cold Work on Crack Initiation in a Light Water Environment Using the Small-Punch Test, Isselin J., Kai A., Sakaguchi K., Shoji T., Copyright (2008).

42 Obtained through the Attribution 4.0 International (CC BY 4.0) licence. The full details of the license are available at: http://creativecommons.org/licenses/by/4.0.

43 Reprinted from Acta Materialia, 61(13), Dugdale H., Armstrong D. E. J., Tarleton E., Roberts S. G., Lozano-Perez S., 2013, How Oxidised Grain Boundaries Fail, pp. 4707–4713, Copyright (2013), with permission from Elsevier.

44 Reprinted from Journal of Nuclear Materials, 462, Armstrong D. E. J., Hardie C. D., Gibson J. S. K. L., Bushby A. J., Edmondson P. D., Roberts S. G., Small-Scale Characterisation of Irradiated Nuclear Materials: Part II Nanoindentation and Micro-Cantilever Testing of Ion-Irradiated Nuclear Materials, pp. 374–381, Copyright (2015), with permission from Elsevier

45 Reprinted from Journal of Nuclear Materials, 462, Armstrong D. E. J., Hardie C. D., Gibson J. S. K. L., Bushby A. J., Edmondson P. D., Roberts S. G., Small-Scale Characterisation of Irradiated Nuclear Materials: Part II Nanoindentation and Micro-Cantilever Testing of Ion-Irradiated Nuclear Materials, pp. 374–381, Copyright (2015), with permission from Elsevier.

46 Reprinted from Scripta Materialia, 127, Deng Y., Hajilou T., Wan D., Kheradmand N., Barnoush A., In-situ micro-cantilever bending test in environmental scanning electron microscope: Real time observation of hydrogen enhanced cracking, pp. 19–23, Copyright (2017), with permission from Elsevier.

47 Reprinted from 21st International Conference on Water Chemistry in Nuclear Reactor Systems Conference, San Francisco, 2018, Holmes R., Burrows R., Dickinson S., Walters W. S., Platts L., Clark R. N., Harrington C., Baron-Wiechec A., Surrey E., Martin T. L., Springell R. S., Warren A., Siberry A., Bertaux B., Chemistry and Corrosion in the Irradiated Cooling Circuits of a Prototype Fusion Power Station, Copyright (2018).

## Footnotes

41 Reprinted from Scripta Materialia, 61(7), Armstrong D. E. J., Rogers M. E., Roberts S. G., Micromechanical Testing of Stress Corrosion Cracking of Individual Grain Boundaries, pp. 741–743, Copyright (2009), with permission from Elsevier.
